# Supplementary material for: Achieving a solar-to-chemical efficiency of 3.6% in ambient conditions by inhibiting interlayer charges transport
Source: Nat Commun. 2024 Jun 26;15:5406. doi: 10.1038/s41467-024-49373-z (PMC11208529; doi:10.1038/s41467-024-49373-z)
Supplement: Supplementary file 1 — Supplementary Information [file 41467_2024_49373_MOESM1_ESM.pdf]

# Supplementary Materials for

# Achieving a solar-to-chemical efficiency of 3.6% in ambient conditions by inhibiting interlayer charges transport

Yuyan Huang[1], Minhui Shen[1], Huijie Yan[2], Yingge He[3], Jianqiao Xu[1], Fang Zhu[1], Xin Yang[3], Yu-Xin Ye[2,4]*, Gangfeng Ouyang[1,2,4]*

[1]Key Laboratory of Bioinorganic and Synthetic Chemistry of Ministry of Education, LIFM, School of Chemistry, IGCME, Sun Yat-Sen University, Guangzhou 510275, China.

[2]School of Chemical Engineering and Technology, IGCME, Sun Yat-sen University, Zhuhai 519082, China.

[3]School of Environmental Science and Engineering, Guangdong Provincial Key Laboratory of Environmental Pollution Control and Remediation Technology, Sun Yat-sen University, Guangzhou 510275, China.

[4]Southern Marine Science and Engineering Guangdong Laboratory (Zhuhai), Zhuhai, Guangdong, 519082, China.

*Corresponding author. E-mail: yeyuxin5@sysu.edu.cn; cesoygf@mail.sysu.edu.cn

# Supplementary Methods

## Materials

2,6,14-Tribromotripterene (97%), 1,3,6,8-tetrabromopyrene (97%), 2,3,6,7,10,11-hexabromotriphenylene (97%), 2,6-diaminoanthraquinone and potassium iodide were purchased from Tensus. Sodium nitrite (99.8%), trimethylamine (≥99.5%), bis(triphenylphosphine)palladium(II) dichloride and tetrabutylammonium fluoride solution (70% aqueous solution) were purchased from Mackin. Trimethylsilylacetylene was purchased from Meryer. Chloroform was purchased from XIHUA. Copper(I) iodide (98%) was obtained from Aladdin. Acetonitrile (99.8%), N, N-dimethylacetamide (99.8%), toluene (99.8%), 5,5-dimethyl-1-pyrroline N-oxide (DMPO, 98%), 2,2,6,6-tetramethyl-4-piperidinol (TEMP, 98%) were obtained from Sigma-Aldrich. Silver nitrate ($AgNO_3$) and ethylenediaminetetraacetic acid disodium salt (EDTA-2Na) were purchased from the Guangzhou Chemical Reagent Factory. The basic characteristics of the actual water were collected in Supplementary Table 2. All materials were used as received without further purification or treatment. Ultrapure water (>18 MΩ·cm) was used for all the experiments.

## Synthesis of 2,6-diethynyl-9,10-anthracenedione (AQ-A)

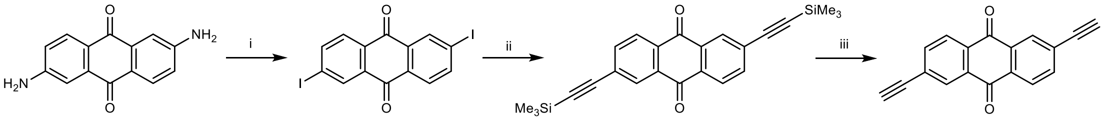


**Supplementary Fig. 1 | Synthetic route to 2,6-diethynyl-9,10-anthracenedione.** The 2,6-diethynyl-9,10-anthracenedione was prepared according to the synthetic route.

**i**. The synthesis of 2,6-diiodoanthracene-9,10-dione (AQ-I) was carried out from a previously reported synthesis[1]. To a 100 mL flask was added 1.005 g 2,6-Diaminoanthraquinone (AQ-$NH_2$, 4.22 mmol), acetonitrile (30 mL) and hydrochloric acid (10 mL 2.4 M), and the suspension was cooled to 0°C. Sodium nitrite (10.1 mmol, 694 mg) was dissolved in 2 mL water, then slowly dropped into

the former suspension. The whole process was taken at 0°C in an ice bath. After 30 min stirring, potassium iodide (21.4 mmol, dissolved in 5 mL water) was added. Then it was allowed to warm up to room temperature, stirring for an hour, and then heat to 60°C for another hour. The crude product was get by filtration, then washed with water and methanol and dried in vacuum to afford a light brown compound of 1.6 g (82% yield). $^1$H-NMR (400 MHz, 298 K) δ (DMSO, ppm): 8.46 (s, 2H), 8.33 (d, 2H), 7.92 (d, 2H).

**ii.** To a stirred solution of diiodoanthraquinone (0.46 g, 1 mmol), CuI (3.8 mg, 20 μmol), and $(PPh_3)_2PdCl_2$ (14 mg, 20 μmol) in triethylamine (4 mL) and toluene (10 mL) under an argon atmosphere trimethylsilylacetylene (300 μL, 2.1 mmol) was added, and the mixture was refluxed for 18.5 h. The cold solution was filtered through a bed of celite, and the solid was washed with an additional amount of toluene (5 mL). The filtrate was then washed with brine and water, and the solvent was evaporated under reduced pressure. The residual solid was washed with hot methanol[2]. The Coarse product was depurated by column chromatography with silica gel (n-Hexane/$CH_2Cl_2$, 10:1, v/v) and gained pure product as yellow crystals (yield 375 mg, 94%), m.p. = 180–182°C. $^1$H NMR (400 MHz, Chloroform-d, 25°C, TMS) δ = 8.36 (d, 2H), 8.25 (d, 2H), 7.83 (dd, 2H), 0.29 (s, 18H).

**iii**. The bis(trimethylsilylethynyl)-9,10-anthraquinone (AQ-$SiMe_3$, 0.2 g, 0.5 mmol) was dissolved in chloroform (10 mL) and heated at reflux. To this mixture, a solution of tetrabutylammoniumfluoride (0.42 g, 1.5 mmol) in $CHCl_3$ (5 mL) was added dropwise during 1 h. After stirring at room temperature for more than 2 h, water (10 mL) was added and the organic layer was washed with brine and with water. After the removal of the solvent, the solid residue was thoroughly washed with methanol and vacuum dried[2]. Yield: (105 mg, 82%), light-brown solid. M. p. >250°C. $^1$H NMR (400 MHz, DMSO, 25°C, TMS), δ = 8.19 (d, 2H), 8.16 (d, 2H), 7.99 (dd, 2H), 4.68 (s, 2H).

**Synthesis of photocatalyst**

The conjugated polymeric photocatalysts were synthesized according to

Sonogashira-Hagihara cross-coupling polycondensation.

Synthesis of TPC-3D: For the synthesis of TPC-3D, a dry 50 mL round-bottom flask was charged with 2,6,14-tribromotripterene (49.1 mg, 0.1 mmol), 2,6-diethynyl-9,10-anthracenedione (51.6 mg, 0.2 mmol), $Pd(PPh_3)Cl_2$ (7.02 mg, 0.01mmol), CuI (1.91 mg, 0.01mmol), and a mixed solvent of 5 mL dimethyl formamide (DMF) and 5 mL triethylamine (TEA). The mixture was degassed by bubbling with Ar for 30 min, and then the resulting mixture was stirred at 80°C for 24 h under Ar condition. After that, the precipitate was collected by filtration, and the solid was washed with methanol and $CH_2Cl_2$ (1:1) in the Soxhlet for 48 h. The final product was dried at 60◦C overnight.

Synthesis of PYR-2D: For the synthesis of PYR-2D, a dry 50 mL round-bottom flask was charged with 1,3,6,8-tetrabromopyrene (51.78 mg, 0.1 mmol), 2,6-diethynyl-9,10-anthracenedione (64.5 mg, 0.25 mmol), $Pd(PPh_3)Cl_2$ (7.02 mg, 0.01mmol), CuI (1.91 mg, 0.01mmol), and a mixed solvent of DMF/TEA (5 mL/5 mL). The mixture was degassed by bubbling with Ar for 30 min, and then the resulting mixture was stirred at 80°C for 24 h under Ar condition. After that, the precipitate was collected by filtration, and the solid was washed with methanol and $CH_2Cl_2$ (1:1) in the Soxhlet for 48 h. The final product was dried at 60◦C overnight.

Synthesis of TPL-2D: For TPL-2D, 2,3,6,7,10,11-Hexabromotriphenylene (70.17 mg, 0.1 mmol), 2,6-diethynyl-9,10-anthracenedione (90.3 mg, 0.35 mmol), $Pd(PPh_3)Cl_2$ (7.02 mg, 0.01mmol), CuI (1.91 mg, 0.01mmol), DMF (5 mL), and TEA (5 mL) were added in a dry 50 mL round-bottom flask. Then, TPL-2D was synthesized via the same procedure and the same conditions as TPC-3D.

## Characterization

$^1$H nuclear magnetic resonance (NMR) spectra were carried out on a 400 MHz spectrometer (Bruker ascend 400 MHz NMR spectrometer). Solid-state $^{13}$C NMR spectra were performed on a CyroProbe TCT 600 MHz NMR spectrometer. The infrared spectra were recorded using a PerkinElmer Fourier transform-infrared (FT-IR) spectrometer between 4000–500 cm$^{-1}$. Raman spectra were collected using a Laser

Microscopic Confocal Raman Spectrometer (inVia Qontor, Renishaw) at 325 nm. UV-visible spectroscopy (UV-Vis) was measured on a Shimadzu UV-3600 spectrometer. The crystalline phases were analyzed via a D-MAX 2200 VPC powder X-ray diffraction (PXRD) instrument. Steady-state photoluminescence (PL) spectra were collected by a FLS980 spectrometer. Brunauer-Emmett-Teller (BET) measurements were performed on an iPore400 instrument (PhysiChem Instruments Ltd.). Physical adsorption of $O_2$ was reported in an ASAP2020C instrument. Kelvin probe force microscopy (KPFM) images were obtained from an AFM instrument (Bruker Dimension Icon). X-ray photoelectron spectroscopy (XPS) measurements were characterized by using an ESCALab 250 spectrometer. Transmission electron microscopy (TEM, JEM-ARM200P, Japan) and scanning electron microscope (SEM, SU8010 emission scanning electron microscope, Japan) that operated at 10 kV were used to analyze the morphologies of polymers. The thermal stability was evaluated by thermal gravity analysis (TGA, TG209F1 libra) with a ramping rate of 10 $^{\circ}C \cdot min^{-1}$ under air atmosphere.

**Apparent quantum yield measurements**

The apparent quantum yield (AQY) of photocatalysts was carried out in pure deionized water or seawater sample (54 mL) with photocatalysts (10 mg) in a quartz tube. Then, irradiated under a Xe lamp for 1h with magnetic stirring. The number of incident photons (M) was calculated by:

$$\mathrm{M} = \frac{E\lambda}{hv} \tag{1}$$

In the equation, $E, \lambda$, $h$, and $v$ refers to the average intensity of irradiation, the Planck constant, and the speed of light, respectively. The AQY was calculated from the following equation:

$$\mathrm{AQY} = \frac{2 \times number\ of\ evolved\ H_2O_2\ molecules}{number\ of\ incident\ photons} \times 100\% \tag{2}$$

**Temperature-programmed desorption (TPD)**

Samples (50–100 mg) were first pretreated at 200 °C for 1 h under He atmosphere (30–50 $mL \cdot min^{-1}$) and then cooled to 50°C in He. After that, the samples were

exposed to 10% (v/v) $O_2$/He gas mixture (30–50 mL·min$^{-1}$) for 1 h until saturation. Then, the samples were purged by He for 1 h to eliminate the physically adsorbed $O_2$ and then heated in He from room temperature to 250 ◦C/325 ◦C with a ramping rate of 10 ◦C·min$^{-1}$. Gas chromatograph equipped with a thermal conductivity detector (GC-TCD) was used to quantify $O_2$.

**Rotating disk electrode (RDE) measurements**

The electron transfer number for the oxygen reduction reaction was carried on a rotating disk electrode (RDE) in $O_2$-saturated 0.1 M phosphate buffer solution (pH = 7) at room temperature and a scan rate of 0.01 V·s$^{-1}$ with different rotation speeds after $O_2$ bubbling for 30 min. The average of electron transfer number (n) was estimated by linear regression of the plots using the Koutecky-Levich equation as follows:

$$\frac{1}{J} = \frac{1}{J_L} + \frac{1}{J_K} = \frac{1}{B\omega^{1/2}} + \frac{1}{J_K} \tag{3}$$

$$\mathrm{B} = 0.2\mathrm{nF}V^{-\frac{1}{6}}CD^{\frac{2}{3}} \tag{4}$$

Where J refers to the current intensity, $J_L$ and $J_k$ are the kinetic and diffusion-limiting current densities, respectively. ω is the angular velocity, n is the transferred electron number, F is Faraday constant (96485 C·mol$^{-1}$), ν is the kinetic viscosity of water (0.01 cm$^2$·s$^{-1}$), C is the bulk concentration of $O_2$ in water (1.26 × 10$^{-3}$ mol·cm$^{-3}$), and D is the diffusion coefficient of $O_2$ (2.7 × 10$^{-5}$ cm$^2$·s$^{-1}$).

**Rotating ring-disk electrode (RRDE) measurements**

A ring-disk electrode served as the working electrode in a 0.1 M phosphate buffer solution (pH=7) at room temperature under Ar atmosphere. The phosphate buffer solution was purged with Ar for 30 min before the measurements. The potential of the ring electrode was set to -0.23 and 0.6 V (vs. Ag/AgCl) to detect $O_2$ or $H_2O_2$, respectively.

**Electron paramagnetic resonance (EPR) measurements**

The EPR measurement was carried out on a spectrometer (JES-FA200) to detect oxygen-active species by adding 5,5-dimethyl-1-pyrroline N-oxide (DMPO) as a spin-trapping reagent to detect ·OH or $\cdot O_2^-$, and 2,2,6,6-Tetramethylpiperidine (TEMP)

as a spin-trapping reagent to detect $^1O_2$. The dispersion was purged with $O_2$ gas for 3 min before light irradiation. A Xe lamp (λ>400 nm) was used as the light source. The measurements were conducted as follows: catalyst (1 mg) was dispersed in ultrapure water (250 μL) containing DMPO (10 μL) with a Pyrex glass tube to detect ·OH or $\cdot O_2^-$, and catalyst (1 mg) was dispersed in methanol (250 μL) containing TEMP (10 μL) with a Pyrex glass tube to detect $^1O_2$.

To detect the signal of electrons in the anthraquinone groups, 20 mg of catalyst was loaded into a Pyrex glass tube, then placed it in the spectrometer and illuminated in situ with a xenon lamp for 30 minutes. The light source was removed after 30 minutes of illumination, and the catalyst was left in situ for 30min without light.

**Verification of the ORR sites at the AQ moieties**

Photocatalyst (10 mg) was added to 20 mL deionized water in a beaker and dispersed by ultrasonication for 30 min. Firstly, the suspension was irradiated under Ar atmosphere for 1 h and then bubbled with $O_2$ in the dark for 30 min.

**Verification of the ORR sites at the alkynyl moieties**

1 mg photocatalyst and 10 mM sodium oxalate were added to 50 mL deionized water in a beaker. Then, the mixture was dispersed by ultrasonication for 30 min. The suspension was injected with $O_2$ for 30 min so that alkynyl moieties absorb oxygen. Afterward, the catalyst was irradiated by using a Xe lamp (CEL-HXF300) as the light source under Ar atmosphere for 1 h.

**Verification of the WOR**

1 mg photocatalyst and 10 mM silver nitrate were added to 50 mL deionized water in a beaker. The mixture was dispersed by ultrasonication for 30 min. Afterward, the catalyst was irradiated by using a Xe lamp (CEL-HXF300) as the light source under Ar atmosphere for 1 h.

**Isotopic experiment**

5 mg of photocatalyst was ultrasonically dispersed into 1 mL of $H_2{}^{18}O$, which was purged with $O_2$ for 5 minutes. Then, the suspension was illuminated for 2 h by a 300

W Xe lamp (λ >400 nm, 100 mW·cm$^{-2}$) at ambient temperature. The post-reaction photocatalytic hydrogen peroxide solution was transferred to a new reactor, and Ar was injected to eliminate $O_2$. $MnO_2$ was added to catalyze the decomposition of hydrogen peroxide, and the evolved oxygen gas was analyzed through GC-MS (Agilent 8860-5977B).

**In situ DRIFTS measurements**

In situ diffuse reflectance infrared Fourier transform spectroscopy (DRIFTS) was recorded on a Nicolet 5700 FT-IR spectrometer, and the cell was equipped with a KBr window. The samples were filled into an in-situ IR cell and were degassed at 373 K for 4 h. Then the samples were exposed to Ar gas or $O_2$ or $H_2O$ vapor for 60 min to reach adsorption-desorption equilibrium.

**Photoelectrochemical measurements**

All measurements were performed on an electrochemical workstation (CHI 760E Instruments) at room temperature in a typical three-electrode cell system, using an Ag/AgCl (saturated KCl) as reference electrode, platinum wire as the counter electrode, the sample-modified glassy carbon or FTO glass as the working electrode. The illumination source is a 300 W Xe lamp (CEL-HXF300). All materials were prepared by adding 5 mg catalyst into the solution containing 180 μL ethanol and 20 μL 5% Nafion. Then, the mixture was dispersed by ultrasonication and dropped on top of a glassy carbon working electrode or FTO glass with the size of 2 × 1cm (the coating area was 1 cm$^2$). Unless otherwise specified, measurements were performed under an air atmosphere.

Electrochemistry impedance spectroscopy (EIS) were performed in 0.1 M phosphate buffer solution (pH=7) with glassy carbon as the working electrode. The Mott-Schottky measurements were measured in 0.5 M $Na_2SO_4$ solution with different alternating current frequencies while the FTO glass was used as the working electrode. The photocurrent measurements were carried out in 0.1 M $Na_2SO_4$ solution under Ar or $O_2$ atmosphere and the working electrode was FTO glass as well.

## Supplementary Notes

### Supplementary Note 1 | Calculation process of the VB maxima referenced to the normal hydrogen electrode ($E_{NHE}$)

The VB was determined using VB-XPS ($E_{VB\text{-}XPS}$) to measure the potential difference of the VB to Fermi level (Supplementary Fig. 13). Subsequently, KPFM was used to measure the work function (φ) of the material, which represents the potential difference from the Fermi level to the vacuum level (Supplementary Fig. 14). By summing these two values, the potential difference between the material's VB and the vacuum level was obtained. Subtracting 4.5 from this value yields the VB referenced to the normal hydrogen electrode ($E_{NHE}$), i.e., the HOMO values were calculated using the equation: $E_{NHE} = \varphi + E_{VB\text{-}XPS} - 4.5$. This approach is more accurate than directly using the VB determined by VB-XPS since VB-XPS only measures the potential difference between the Fermi level and VB, and the distance between the Fermi level and CB (Conduction Band) is uncertain. To calculate the work function (φ), calibration with a gold standard sample is necessary. Therefore, the formula for calculating the work function is $\varphi = 5.2 - (V_R - V_{Au})$, where $V_R$ is the measured potential of the material obtained from Supplementary Fig. 14, and $V_{Au}$ is the potential of the gold standard sample. In this experiment, the potential of the gold standard sample ($V_{Au}$) was -0.638 V.

### Supplementary Note 2 | Statement on catalyst stability

TPC-3D was dispersed in pure water for 5 photocatalytic cycles, and essentially maintained its high efficiency, morphology, and component. However, there was partial oxidation in the photocatalytic process. The FT-IR spectroscopy of the material after cycling revealed a decrease in the alkynyl peak, along with the appearance of a new peak near 1700 $cm^{-1}$. This indicated that self-oxidation of the alkynyl sites might occur during the photolysis process. There was a slightly downward trend in the yield of $H_2O_2$ with an increasing number of cycles (Supplementary Fig. 15). To further substantiate the occurrence of oxidation at alkynyl, we synthesized a polymer devoid

of alkynyl groups using triptycenes as the electron donor and anthraquinone as the electron acceptor, designated as TPC-AQ (Supplementary Fig. 18a). Remarkably, the infrared spectrum of TPC-AQ without alkynyl group exhibited no significant changes after five cycles (Supplementary Fig. 18b). It is inferred that some strong oxidizing substances were generated on the alkynyl during the photocatalytic process, which led to the self-oxidation of alkynyl.

In order to identify the species causing oxidation, the electron paramagnetic resonance (EPR) spectra measurement was conducted. The results indicated that strong oxidizing nature of $\cdot O_2^-$ likely leads to its attack on the alkynyl, causing the observed changes in the infrared spectrum (More details will discuss in the "Photochemical process" section in the main text).

Nonetheless, this partial oxidation exerts a minor effect on the production of $H_2O_2$, resulting in only a slight reduction in its yield (Supplementary Fig. 15). This is attributed to the relatively small percentage of ORR occurring at the alkynyl group (More details will discuss in the "Photochemical process" section in the main text).

To solve the problem of self-oxidation, we immobilized TPC-3D on a glass slide to construct a device for the cycle experiment (Fig. 2c). TPC-3D exhibited a stable $H_2O_2$ yield over 15 cycles (Fig. R2d). The FT-IR Spectroscopy displayed that its alkyne peaks were still clearly visible after 15 cycles (Supplementary Fig. 19a), and there were no significant changes in other characterizations (Supplementary Fig. 19b–d).

## Supplementary Note 3 | Effect of elements in water on photocatalytic performance

Ion concentration in real water had been listed in Supplementary Table 4, but there does not appear to be a direct correlation between performance and $Cl^-$, $SO_4^{2-}$, $NO_3^-$, and $Br^-$ concentrations. In order to investigate the effects of extra elements, photocatalytic experiments were carried out by adding 5 mM of $Cl^-$, $SO_4^{2-}$, $NO_3^-$, $Br^-$, and $HCO_3^-$ to pure water. As shown in Supplementary Fig. 23, $Cl^-$, $SO_4^{2-}$, $NO_3^-$, and $Br^-$ had no significant effect on the photocatalytic performance, indicating that

TPC-3D demonstrated exceptional resistance to interference. However, the addition of $HCO_3^-$ led to an improvement in photocatalytic performance. This improvement was attributed to $HCO_3^-$ acting as a hole scavenger, suppressing the hole-mediated $H_2O_2$ oxidation ($H_2O_2 + 2h^+_{VB} \rightarrow O_2 + 2H^+$), and facilitate the generation of electrons[3].

**Supplementary Note 4 | Reaction of electrons and holes from light to dark**

As depicted in the Supplementary Fig. 35a, the single Lorentzian line of TPC-3D was detected in $H_2O$ with the center g factor of 2.0038, which derived from the unpaired electrons in the oxygen-centered by the storage of one electron in AQ[4]. This signal was gradually enhanced with the increasing light duration which was due to electrons generated by photoexcitation were stored on AQ to form a single-electron stored AQH through intramolecular transfer, and reached saturation at about 10 min (Supplementary Fig. 35b).

Later, turned off the light and the EPR signal decreased rapidly over a 10-minute period, after which it progressively stabilized (Supplementary Fig. 35a, b). There are three possible mechanisms that could lead to the signal decrease after the light is turned off: the single electrons on AQH recombine, react with oxygen, or react with $H_2O$. To investigate the cause of the signal reduction, the solid was directly exposed to air for EPR measurement (Supplementary Fig. 35c). The results showed that signal intensity had no significant change before and after the lights were turned off for 1 h, indicating that the single-electron stored on AQH neither recombine nor directly react with $O_2$ in air. Therefore, the decline in the signal can be attributed to the reaction of electrons with $H_2O$. Specifically, the single-electron stored AQH gains an additional electron from $H_2O$ to form the two-electron stored $AQH_2$ which subsequently reacted with $O_2$ to release $H_2O_2$, leading to the signal decrease[5]. In order to demonstrate that AQH receives electrons from $H_2O$, EPR measurement was conducted in SafeDry acetonitrile to eliminate the interference of $H_2O$. As shown in Supplementary Fig. 35d, e, there was no significant change in the signal under both light irradiation and light off conditions, confirming that $H_2O$ is the crucial electron donor.

On the other hand, electrons and holes are formed in pairs. The changes of electrons during the reaction have been discussed above. As for holes, under light irradiation, the excited CPs generates photo-induced holes that can participate in either a four-electron water oxidation reaction (WOR) to produce $O_2$ or a two-electron WOR to yield $H_2O_2$. Rotating ring-disk electrode (RRDE) measurement confirmed the existence of both pathways in CPs (Supplementary Fig. 24). Moreover, under bubbling argon to remove $O_2$ from the air, with the incorporation of electron sacrificial agents ($AgNO_3$) to inhibit the generation of $H_2O_2$ from ORR, the formation of $H_2O_2$ could also be observed (Supplementary Fig. 28), along with the $H_2{}^{18}O$ isotope experiments (Supplementary Fig. 29), demonstrating that target product $H_2O_2$ can be generated via WOR pathway. The time-dependent DFT (TD-DFT) calculations indicated that all the active sites were mainly located on the TPC and alkynyl moieties for WOR, as the holes were primarily occupied in these two sites in the excited states (Fig. 3a and Supplementary Fig. 25). After removing the light source, the remaining holes are less susceptible to recombine with electrons which have been stored. Moreover, due to the absence of hole storage sites, it cannot be stabilized and can only be consumed by reacting with $H_2O$ or by engaging in alternative annihilation pathways. Afterwards, new holes are no longer produced due to the lack of external light excitation.

**Supplementary Note 5 | Reason for the different pathways on alkynyl and AQ, and the exclusion of the excited donor from reacting with oxygen**

There are two distinct pathways for ORR: one occurs on the alkynyl where it can spontaneously adsorb $O_2$ (Fig. 3e), and then $\cdot O_2^-$ intermediates are formed through a single-electron transfer, subsequently yielding $H_2O_2$ (Pathway I). The other pathway involves the storage of two electrons on AQ, which then reacts with $O_2$ to produce $H_2O_2$ without involving the $\cdot O_2^-$ intermediates (Pathway II). These two pathways coexist, and which one occurs depends on whether oxygen is pre-adsorbed or not. When oxygen is pre-adsorbed on the alkynyl groups, Pathway I will occur; when

there is no pre-adsorbed oxygen on the alkynyl groups, electrons will be rapidly stored on AQ through intramolecular transfer to form $AQH_2$, and subsequently Pathway II will occur.

Additionally, unlike the pathway where electrons are directly transferred to pre-adsorbed $O_2$ forming superoxide, the ORR pathway on AQ first stores electrons to form $AQH_2$, which then reacts with $O_2$. DFT calculations have revealed that this is due to the difficulty of oxygen pre-adsorbing on AQ (Supplementary Fig. 31b). However, once electrons are stored to form $AQH_2$, the energy barrier for oxygen adsorption is significantly reduced (Supplementary Fig. 31c). Subsequently, $AQH_2$ with two-stored electrons undergoes a one-step two-electron ORR to produce $H_2O_2$, bypassing the $\cdot O_2^-$ intermediates.

Regarding the interaction between the excited donor and $O_2$: first, before excitation, DFT calculations indicate that pre-adsorption on the donor is difficult due to high adsorption energy (Supplementary Figs. 31, 37, 38). Upon illumination, and in the absence of pre-adsorbed oxygen on the donor, the excited photocatalysts led to the localization of the photo-induced holes at electron donor, while the electrons at the carbonyl oxygen and carbonyl carbon (Fig. 3a and Supplementary Fig. 25). This means that electrons are rapidly stored on AQ through intramolecular transfer, leaving the holes on the donor. Since the formation of superoxide requires the acquisition of electrons, the electron-deficient excited electron donor at this point is more inclined to obtain electrons from the water oxidation reaction, rather than losing electrons to undergo an oxygen reduction reaction with $O_2$. Therefore, the possibility of the excited electron donor forming superoxide with $O_2$ is relatively small.

**Supplementary Note 6 | Analysis for photocatalytic active area**

The ORR primarily takes place at two distinct sites on TPC-3D: the alkynyl group (site I) and the AQ (site II). As indicated by DFT calculations (Fig. 3e) and in-situ DRIFTS experiments (Fig. 3f), site I acted as a catalytically active site for ORR upon the adsorption of oxygen, and then produced $H_2O_2$. Both the physical and the $O_2$ temperature programmed desorption ($O_2$-TPD) experiment depicted in

Supplementary Fig. 40 reveals that TPC-3D had a significantly higher chemisorption capacity for oxygen compared to PYR-2D and TPL-2D, implying a greater abundance of active alkynyl sites.

For site II, TPC-3D displayed pronounced EPR peaks at g=2.0038 under light irradiation (Supplementary Fig. 33), attributed to the oxygen-centered radicals resulting from the storage of one electron in AQ[4]. TPC-3D exhibited the strongest radical signals among the three conjugated polymers (CPs), indicating a higher number of active AQ sites. In addition, TPC-3D demonstrated the highest water vapor adsorption capacity (Supplementary Fig. 40), benefiting WOR.

In conclusion, TPC-3D exhibited more active sites for ORR and WOR than the photocatalysts with 2D structure. In addition, BET experiments (Supplementary Fig. 39) showed that TPC-3D had a larger specific surface area due to its three-dimensional structure, allowing better exposure of the active sites.

**Supplementary Note 7 | Contribution of amorphous carbon to photocatalytic performance**

In this work, all the photocatalysts employed are amorphous. Amorphous polymers boast a wealth of advantages due to their rich and tunable structures, along with the mild synthesis conditions. In contrast, highly crystalline covalent organic frameworks (COFs) require the use of reversible covalent bonds, which are easily decomposable, and their stringent synthesis conditions significantly limit their application in practical production[6].

These materials as a whole exhibit amorphous properties, but differences in microenvironments impart polarity to each component. They are bifunctional photocatalysts assembled with alternating electron donors and acceptors composed of carbon atoms, which promote the rapid separation and migration of photogenerated electrons and holes for efficient redox reactions.

Specifically, the strategy of alternately linking triptycene with strong electron-donating capability and AQ with strong electron-accepting capability enabled the spontaneous separation of excitons in the excited TPC-3D (Fig. 9a, b). TDDFT

analysis showed that the holes and electrons fully dissociated and distributed across completely different sites (Fig. 3a). In detail, the holes were located at carbon 3 (on triptycene) and carbon 37 (on the acetylene unit), as shown in (Fig. 3b), indicating that the water oxidation reaction occurred at these two sites. $OH^*$ intermediates were adsorbed on the active sites to form the target product $H_2O_2$ through two-electron WOR. Meanwhile, electrons are located on the carbonyl oxygen (atoms 35 and 36) and carbonyl carbon (atoms 27 and 28) of the AQ group (Fig. 3b), pointing to an oxygen reduction reaction at this site. Under visible-light irradiation, AQ sequentially formed two-electron-stored $AQH_2$ via an electron-coupled hydrogenation reaction. Then, adsorption of oxygen was occurred on $AQH_2$ and generated $H_2O_2$. The oxidation and reduction reactions proceed at different sites, allowing the material to efficiently generate $H_2O_2$.

## Supplementary Figures

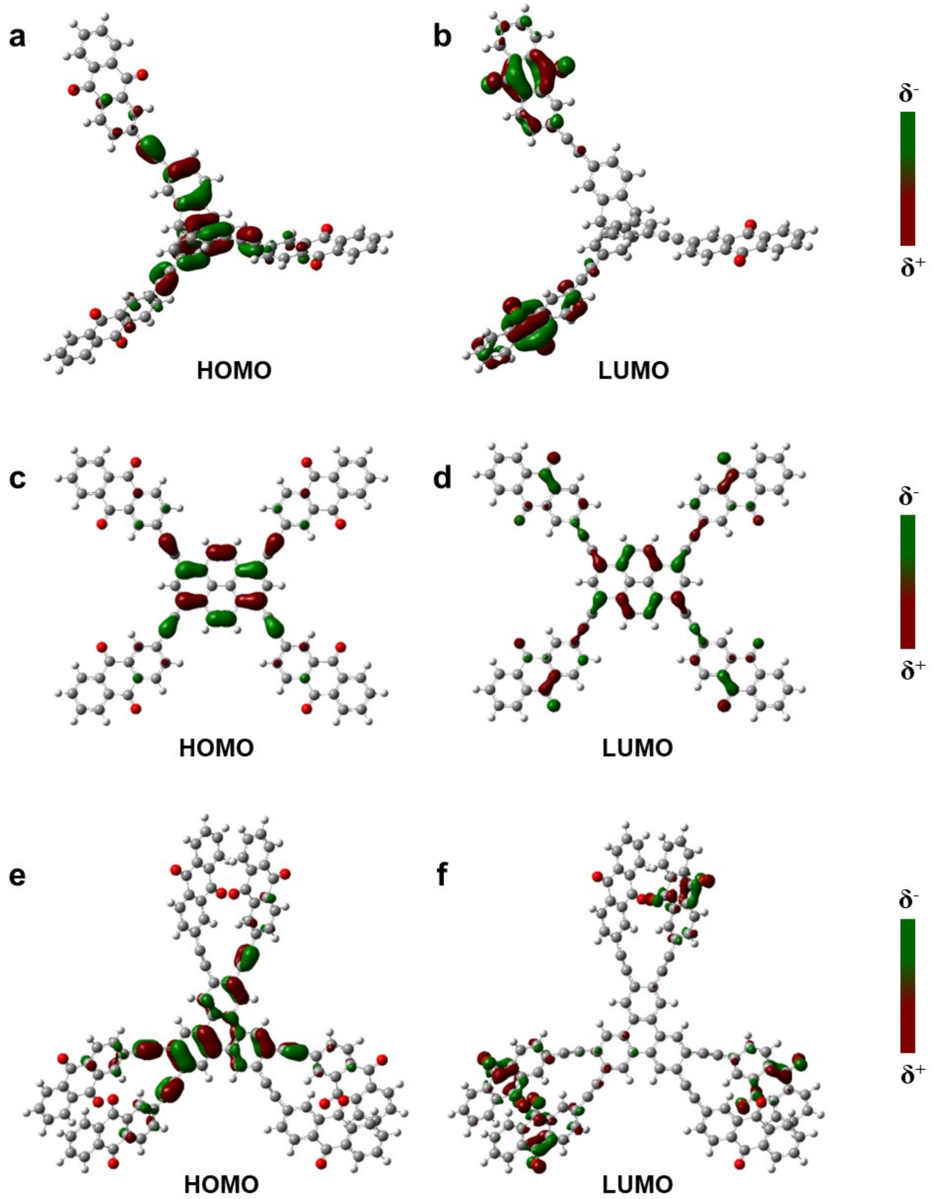


**Supplementary Fig. 2 | Highest Occupied Molecular Orbital (HOMO) and Lowest Unoccupied Molecular Orbital (LUMO) diagrams. a, c, e,** HOMO diagrams of (**a**) TPC-3D, (**c**) PYR-2D, (**e**) TPL-2D. **b, d, f,** LUMO diagrams of (**b**) TPC-3D, (**d**) PYR-2D, (**f**) TPL-2D (Isosurface value = 0.03). HOMO is predominantly located in TPC, PYR and TPL, while LUMO is primarily distributed in the AQ moieties. Thus TPC, PYR, TPL were determined as electron donor, and AQ was defined as electron acceptor. AQ moieties exhibit outstanding electron-accepting capabilities as the LUMO of CPs mainly located in them.

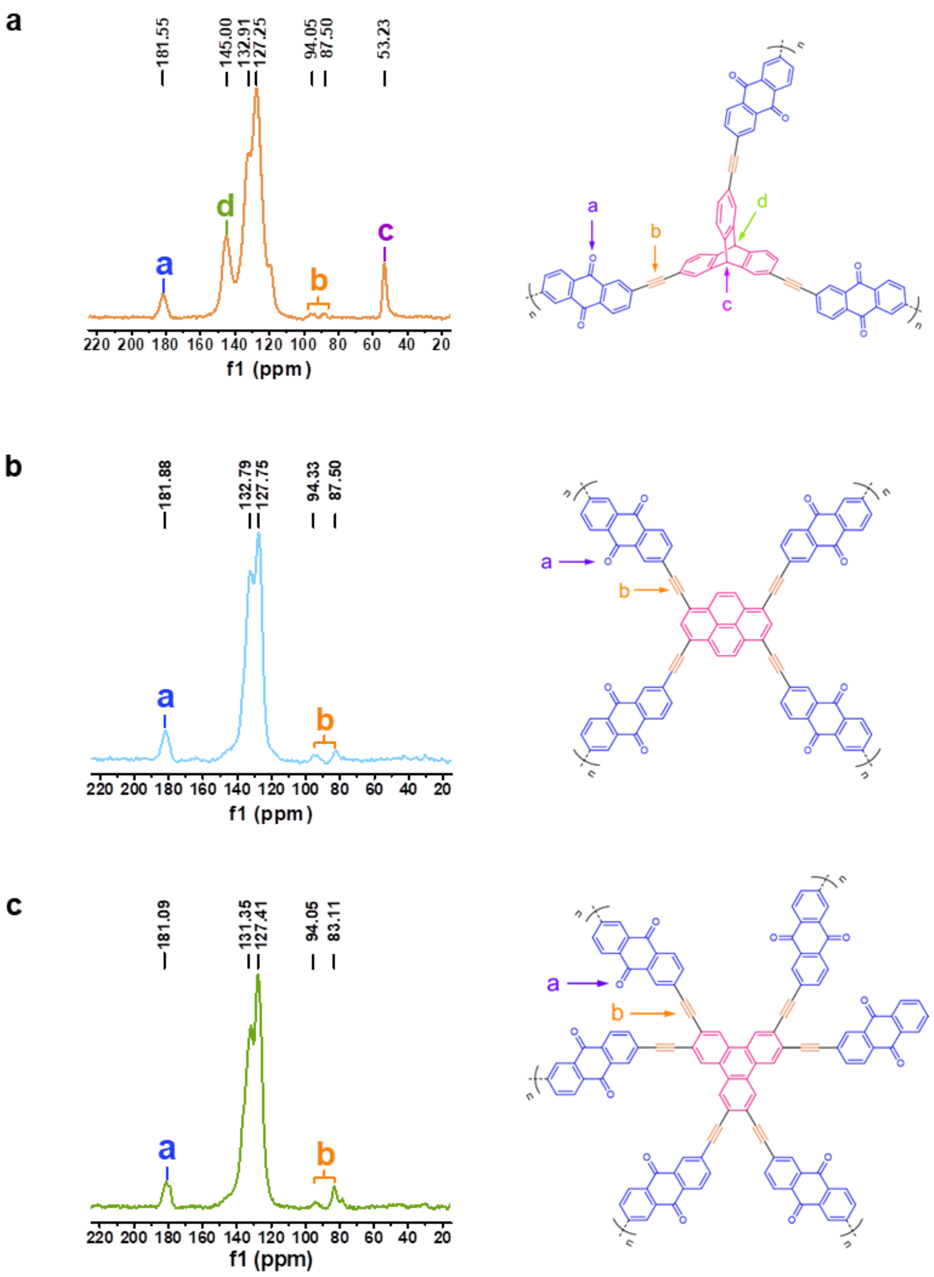


**Supplementary Fig. 3 | Solid-state CP-MAS $^{13}$C nuclear magnetic resonance (NMR) spectra. a–c**, Solid-state CP/MAS $^{13}$C NMR spectra of (**a**) TPC-3D, (**b**) PYR-2D and (**c**) TPL-2D. The strong signals in the range of 119–135 ppm have been assigned to the aromatic carbon skeleton of triptycenes, pyrene and triphenylene. Furthermore, the characteristic signals of the carbonyl group of AQ moieties and alkynyl groups prominently prominently prominently in solid-state CP/MAS $^{13}$C NMR spectra, which demonstrated the successful polymerization of all CPs through the Sonogashira reaction.

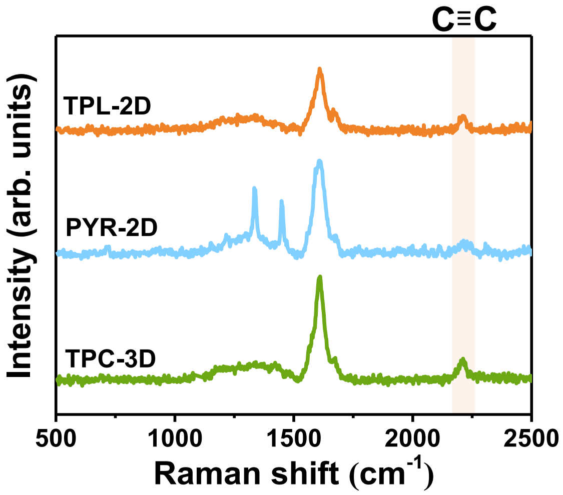


**Supplementary Fig. 4 | Raman spectra of CPs.** The peak at 2214 $cm^{-1}$ proved the existence of alkynyl linkages in CPs. Additionally, the single peak at 1610 $cm^{-1}$ has been attributed to C=C bonds within the aromatic structures. Furthermore, the signals in the range of 1300–1500 $cm^{-1}$ are indicative of the characteristic pyrene vibrations.

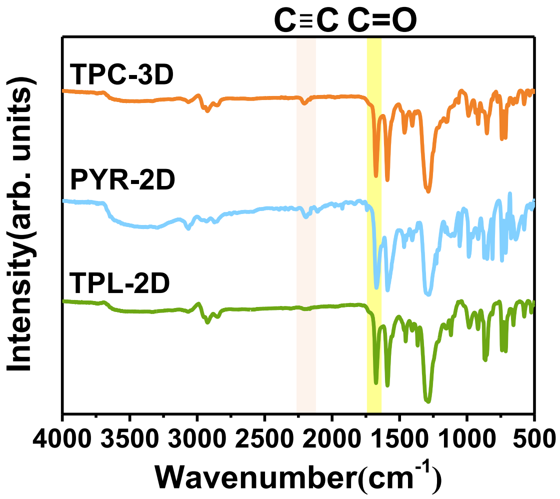


**Supplementary Fig. 5 | Fourier transform infrared (FT-IR) spectra of CPs.** The peak at 1680 $cm^{-1}$ is attributed to the carbonyl group, which evidenced the existence of AQ moieties. Also, the peak at 2200 $cm^{-1}$ proves the existence of alkynyl linkages.

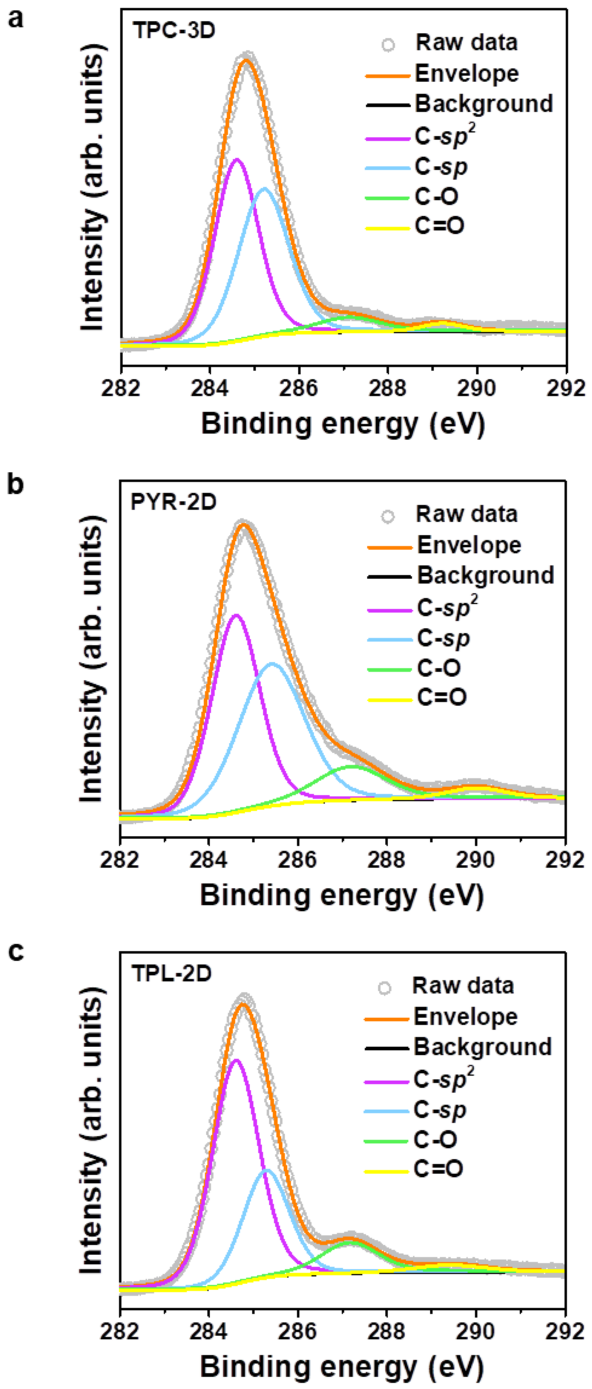


**Supplementary Fig. 6 | The carbon 1*s* X-ray photoelectron spectroscopy (XPS) measurements. a–c**, The C 1*s* XPS of (**a**) TPC-3D, (**b**) PYR-2D and (**c**) TPL-2D can be divided into four contributions: $sp^2$ carbon at 284.6 eV, *sp* carbon at 285.4 eV, C–O at 286.8 eV, and C=O at 288.9 eV, demonstrating the retaining of AQ and alkynyl linkages in CPs.

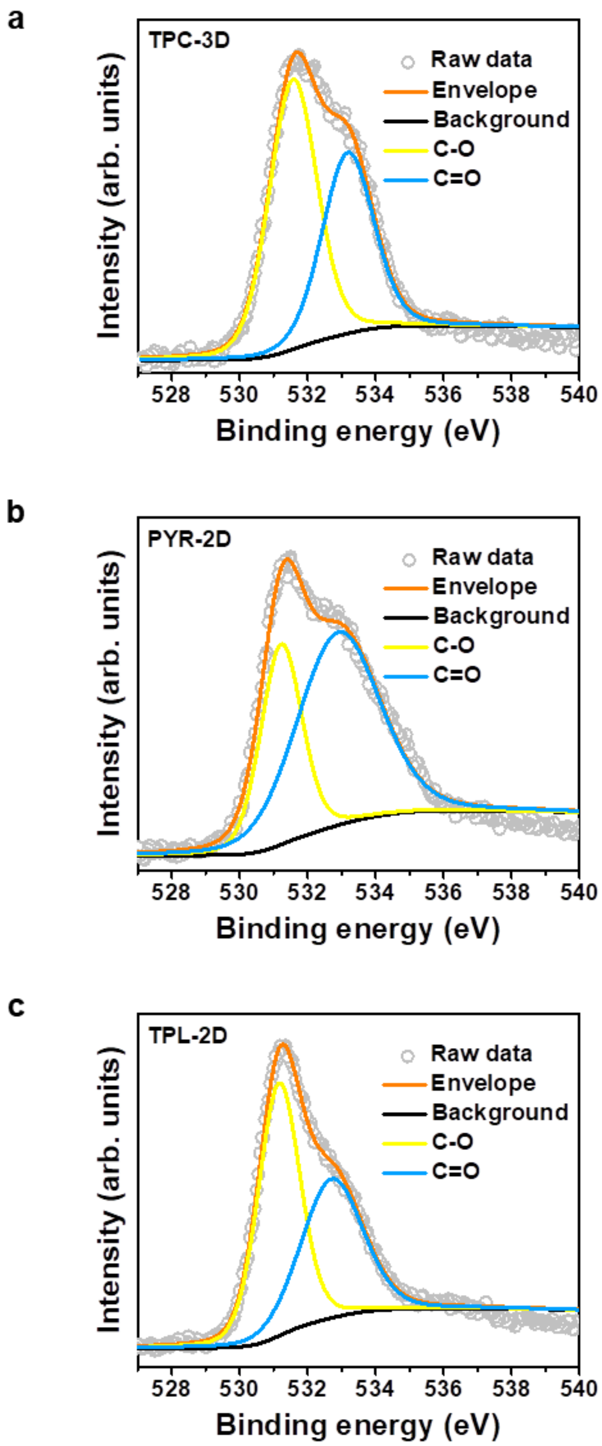


**Supplementary Fig. 7 | The oxygen 1*s* X-ray photoelectron spectroscopy (XPS) measurements. a–c**, The O 1*s* XPS measurements of (**a**) TPC-3D, (**b**) PYR-2D, (**c**) TPL-2D can be divided into two peaks: C-O at 531.7 eV and C=O at 533 eV, indicating the presence of AQ in CPs.

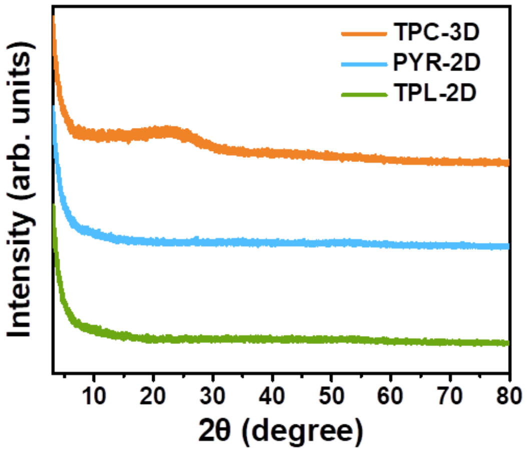


**Supplementary Fig. 8 | Powder X-ray diffraction (PXRD) patterns of CPs.** The PXRD profiles indicate that all the three CPs exhibited the features of amorphous carbon.

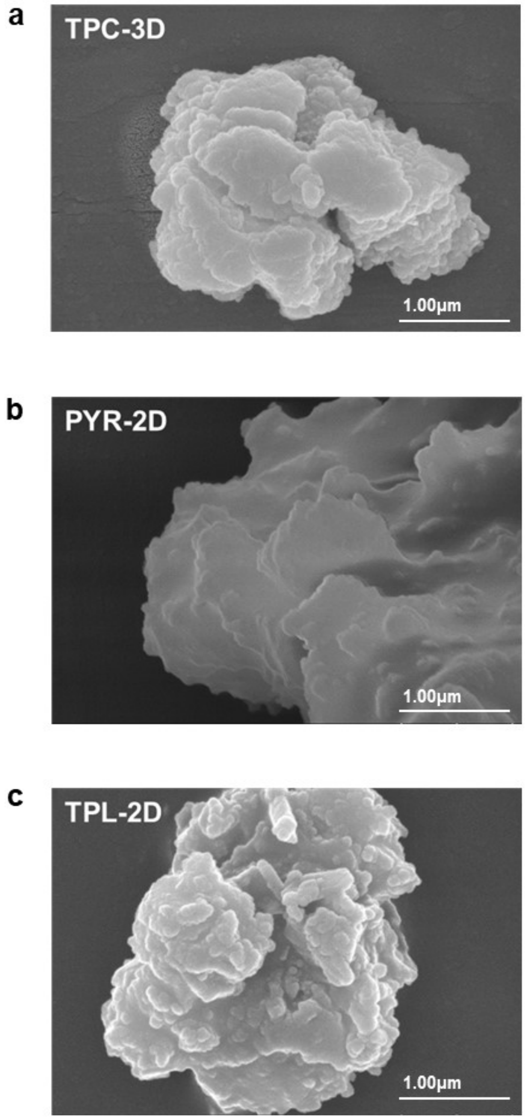


**Supplementary Fig. 9 | Scanning electron microscopy (SEM) images of CPs. a–c**, The SEM images of (**a**) TPC-3D, (**b**) PYR-2D and (**c**) TPL-2D indicate that the CPs possessed rough surfaces and uniform structures.

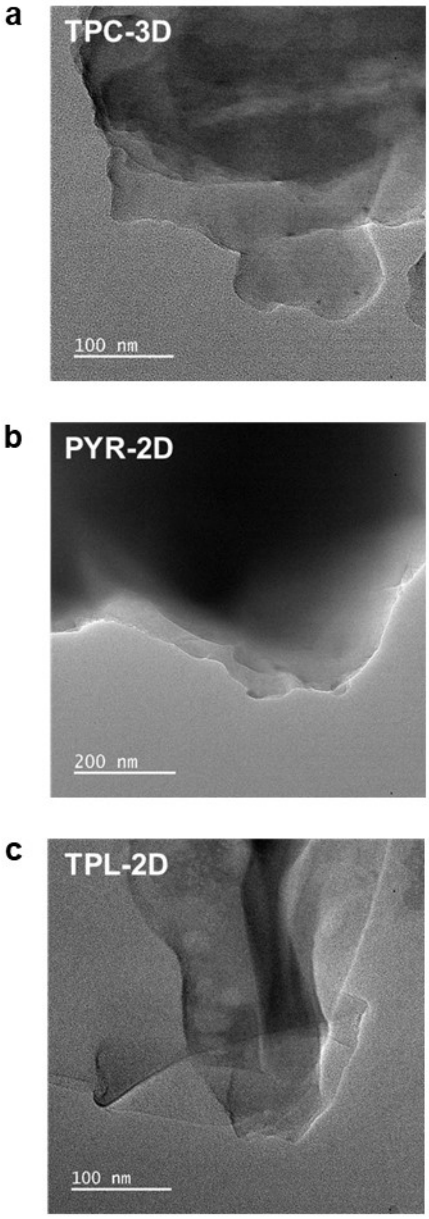


**Supplementary Fig. 10 | Transmission electron microscope (TEM) images of the CPs. a–c**, The TEM images of (**a**) TPC-3D, (**b**) PYR-2D and (**c**) TPL-2D showe that the CPs possessed rough surfaces and uniform structures.

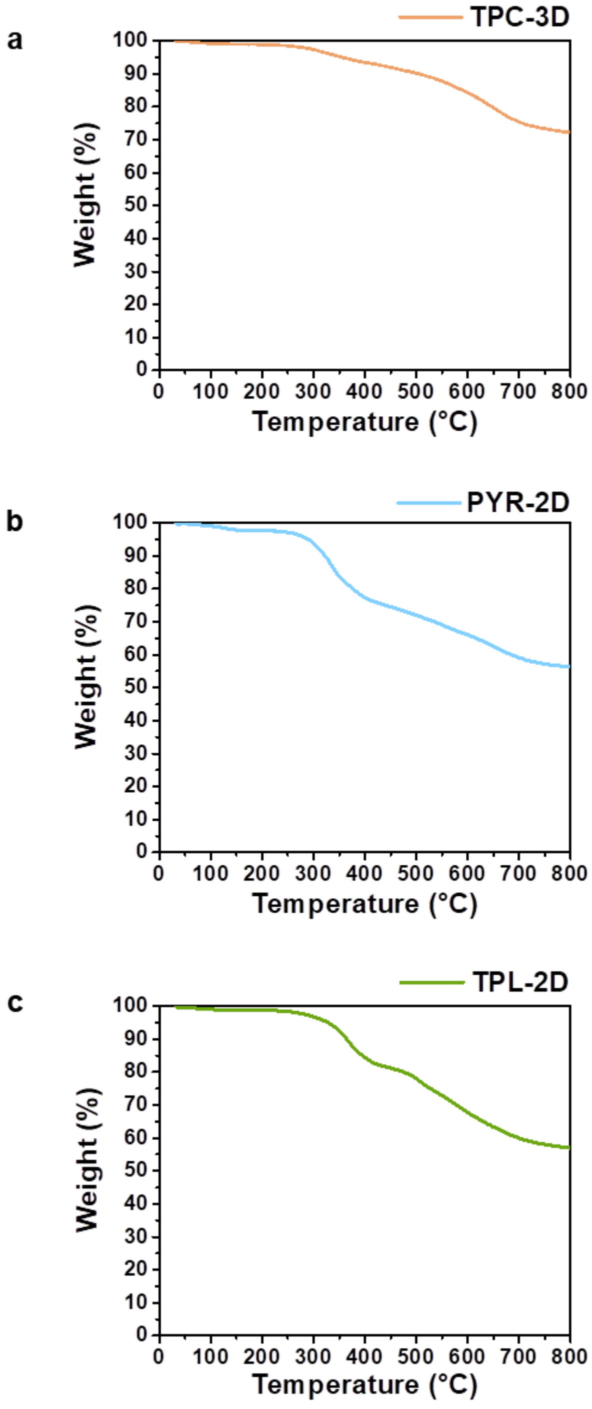


**Supplementary Fig. 11 | Thermogravimetric analysis (TGA) curves. a–c**, TGA curve for (**a**) TPC-3D, (**b**) PYR-2D and (**c**) TPL-2D. All CPs exhibite satisfactory thermal stability.

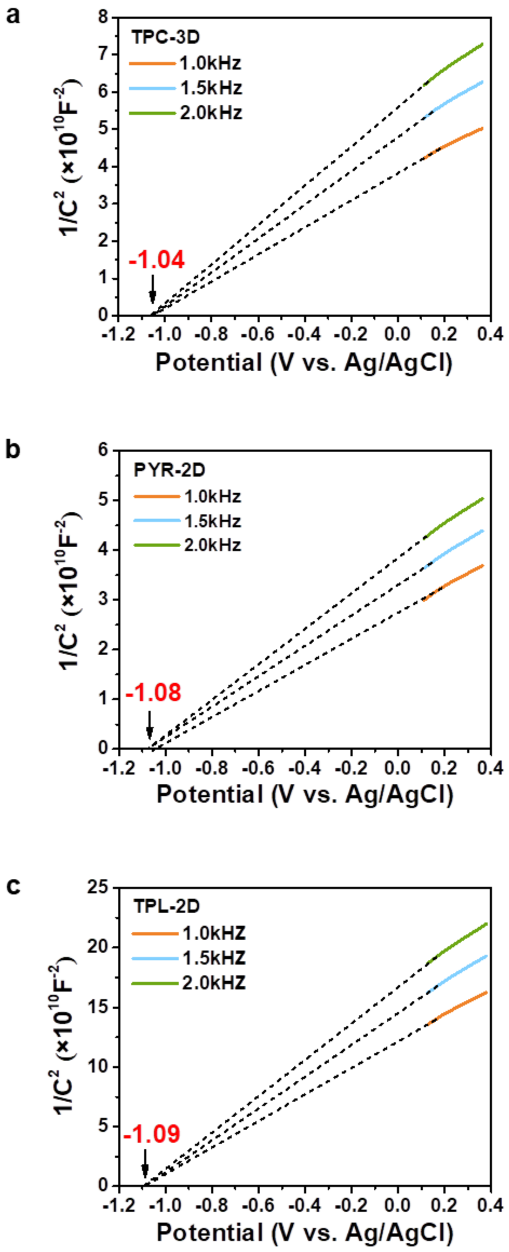


**Supplementary Fig. 12 | Characterization of the conduction band (CB) through Mott Schottky measurements. a–c**, Mott Schottky plots of (**a**) TPC-3D, (**b**) PYR-2D and (**c**) TPL-2D. The conduction band (CB) minima for TPC-3D, PYR-2D and TPL-2D were determined to be -0.43 eV, -0.47 eV, and -0.48 eV versus the reversible hydrogen electrode (RHE) via the Mott-Schottky tests.

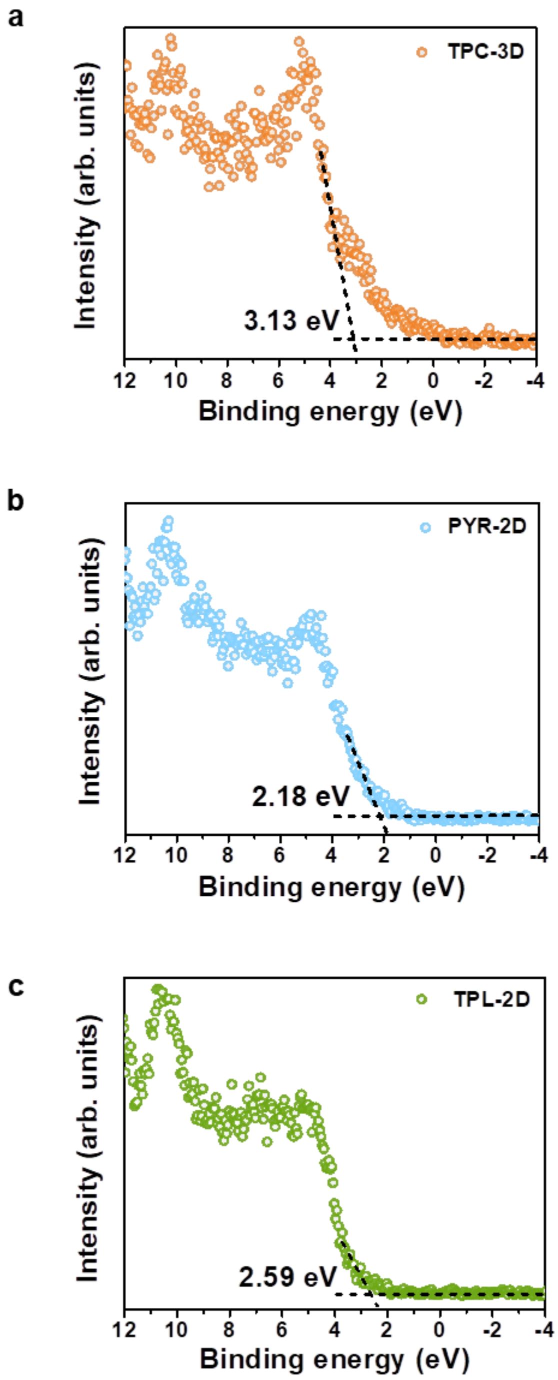


**Supplementary Fig. 13 | Valence band-XPS (VB-XPS) measurements. a–c**, The VB-XPS of (**a**) TPC-3D, (**b**) PYR-2D and (**c**) TPL-2D.

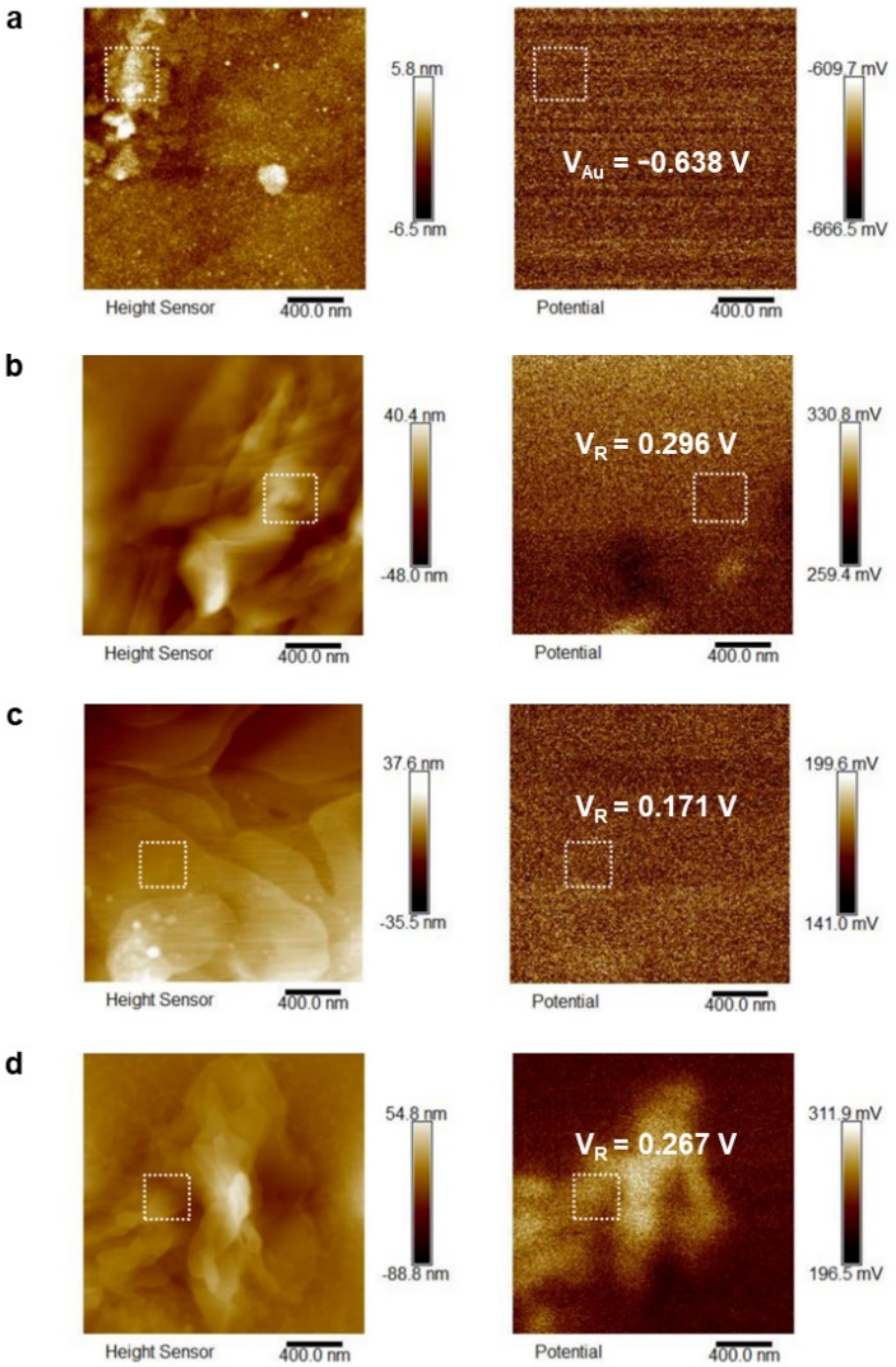


**Supplementary Fig. 14 | Kelvin Probe Force Microscopy (KPFM) surface potential mapping. a–d**, KPFM images of (**a**) Au, (**b**) TPC-3D, (**c**) PYR-2D and (**d**) TPL-2D. The small regions represented the areas used for calculating the work function of the investigated materials.

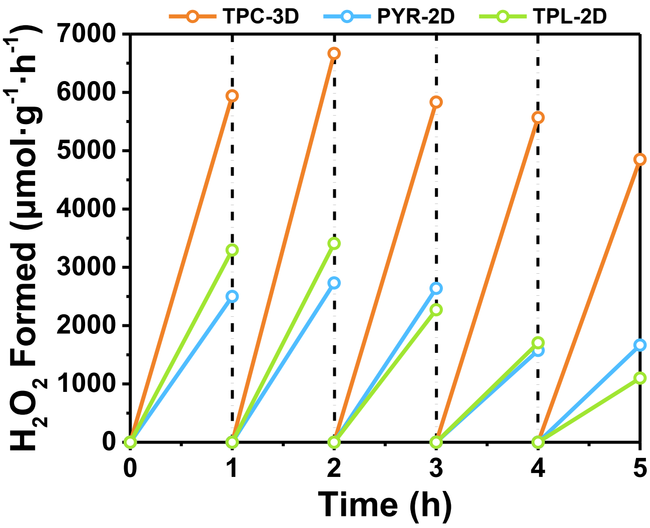


**Supplementary Fig. 15 | Recycle experiments of CPs in pure water.** TPC-3D maintained its high efficiency for 5 cycles in pure water. After 5 cycles, TPC-3D maintained high efficiency of photocatalytic production for hydrogen peroxide in pure water.

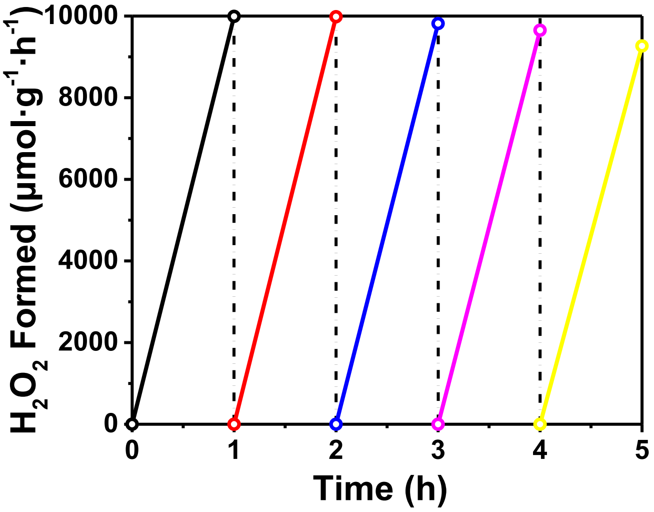


**Supplementary Fig. 16 | Recycle experiments of TPC-3D in lake water.** TPC-3D maintained its high efficiency for 5 cycles in lake water. Even after 5 cycles, TPC-3D maintained high efficiency of photocatalytic production for $H_2O_2$ in lake water.

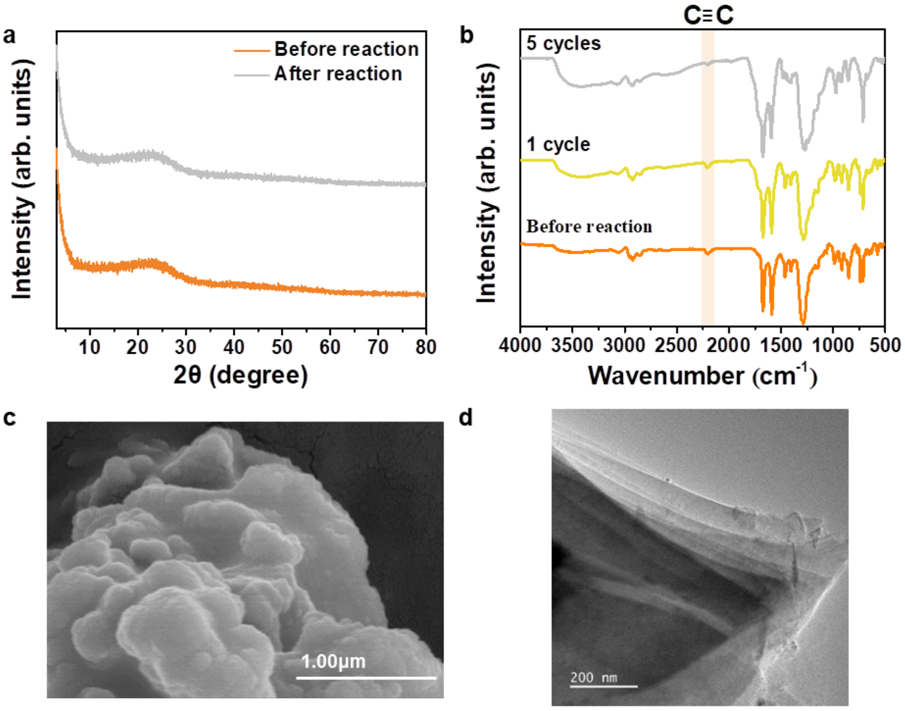


**Supplementary Fig. 17 | Characterizations of TPC-3D after 5 photocatalytic cycles in pure water. a–b**, Comparison between the as-prepared TPC-3D and TPC-3D after 5 cycles of photoreaction: (**a**) PXRD image, (**b**) FT-IR spectra. **c–d**, The (**c**) SEM image and (**d**) TEM image after 5 cycles of recycling.

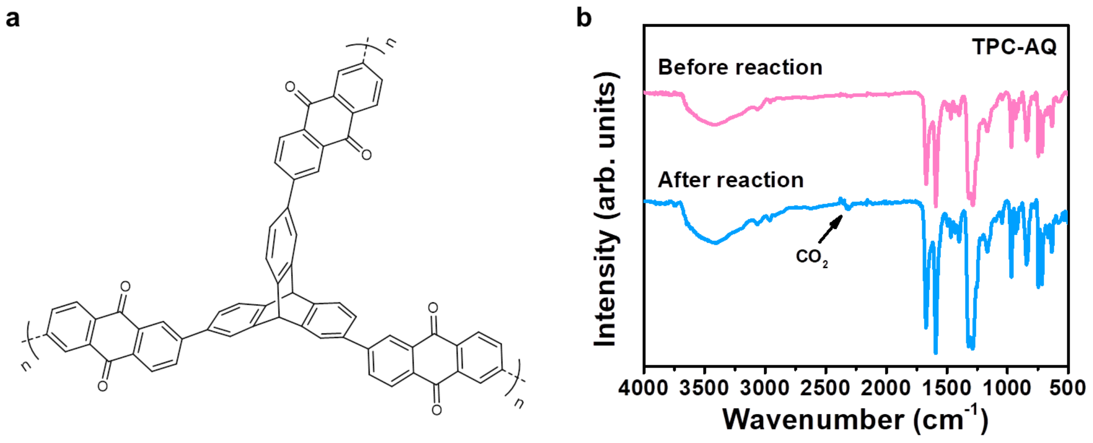


**Supplementary Fig. 18 | Stability of TPC-AQ. a**, Chemical components of TPC-AQ. **b**, The FT-IR spectra before and after 5 cycles of TPC-AQ.

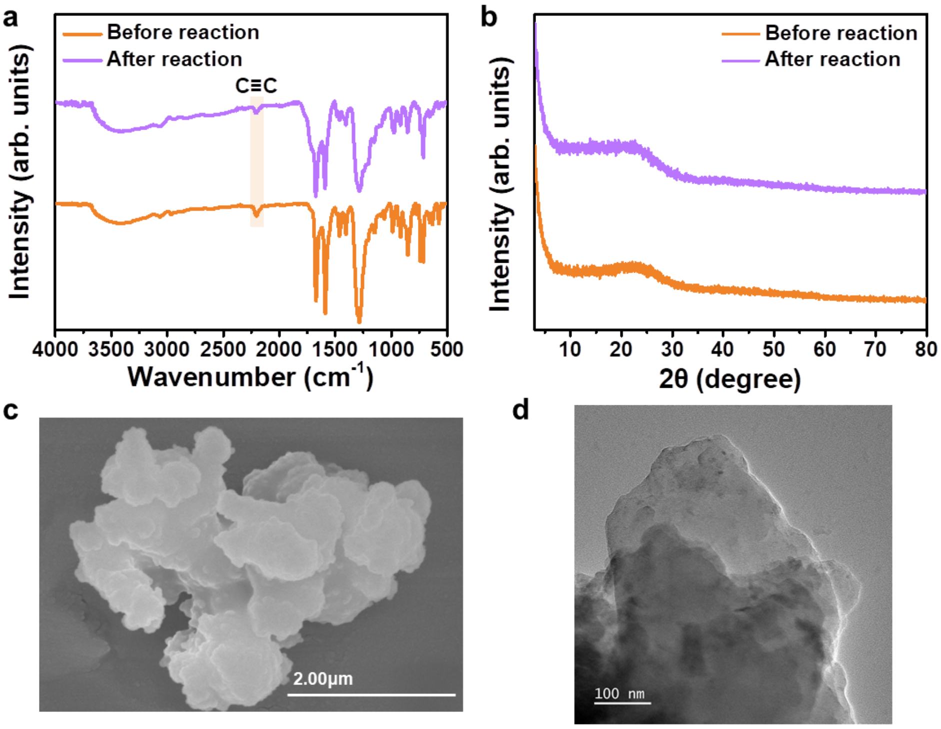


**Supplementary Fig. 19 | Characterizations of TPC-3D immobilized on glass slide after 5 photocatalytic cycles in lake water. a–b**, Comparison between the as-prepared TPC-3D and TPC-3D after 15 cycles of photoreaction: (**a**) PXRD image, (**b**) FT-IR spectra. **c–d**, The (**c**) SEM image and (**d**) TEM image after 5 cycles of recycling.

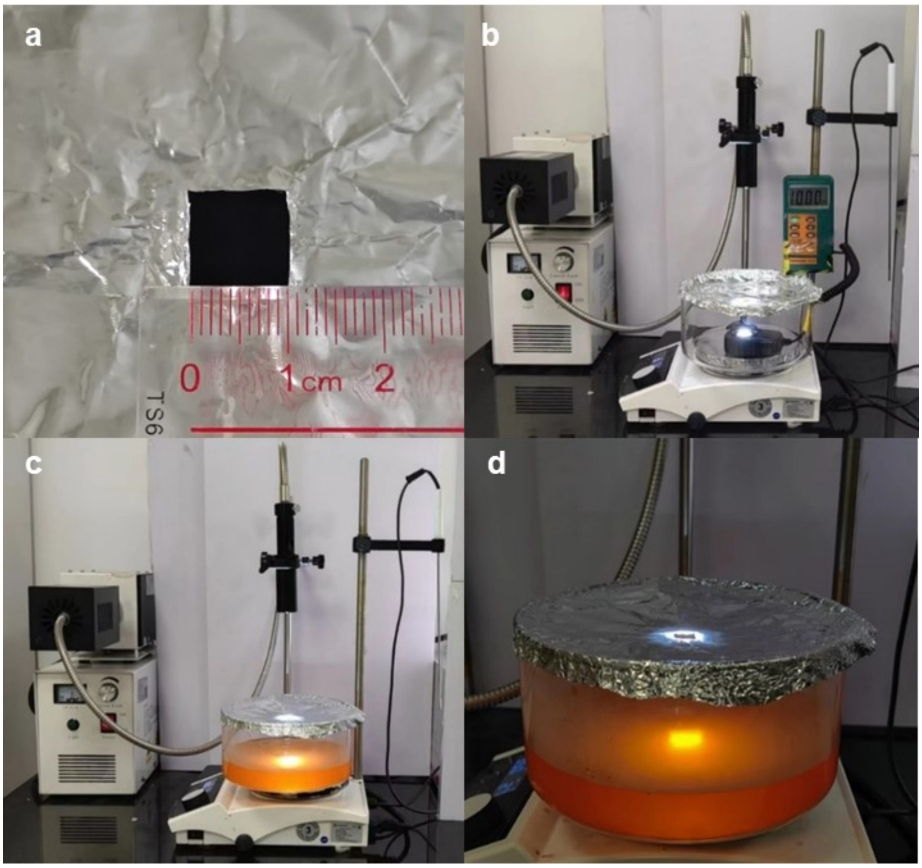


**Supplementary Fig. 20 | The photograph of the device for solar-to-chemical energy conversion (SCC) efficiency measurements.** **a**, Cut out a 1 cm × 1 cm square hole in the middle of an aluminum foil sheet, and covered it on the surface of the container to control the irradiated area to 1 $cm^2$. **b**, Measured the light intensity through the 1 $cm^2$ square hole. **c**, The SCC efficiency measurement of TPC-3D in pure water or lake water. **d**, A light spot on the liquid surface. When illuminating the sample, the light passed through the 1 $cm^2$ hole to form a light spot on the surface of the liquid.

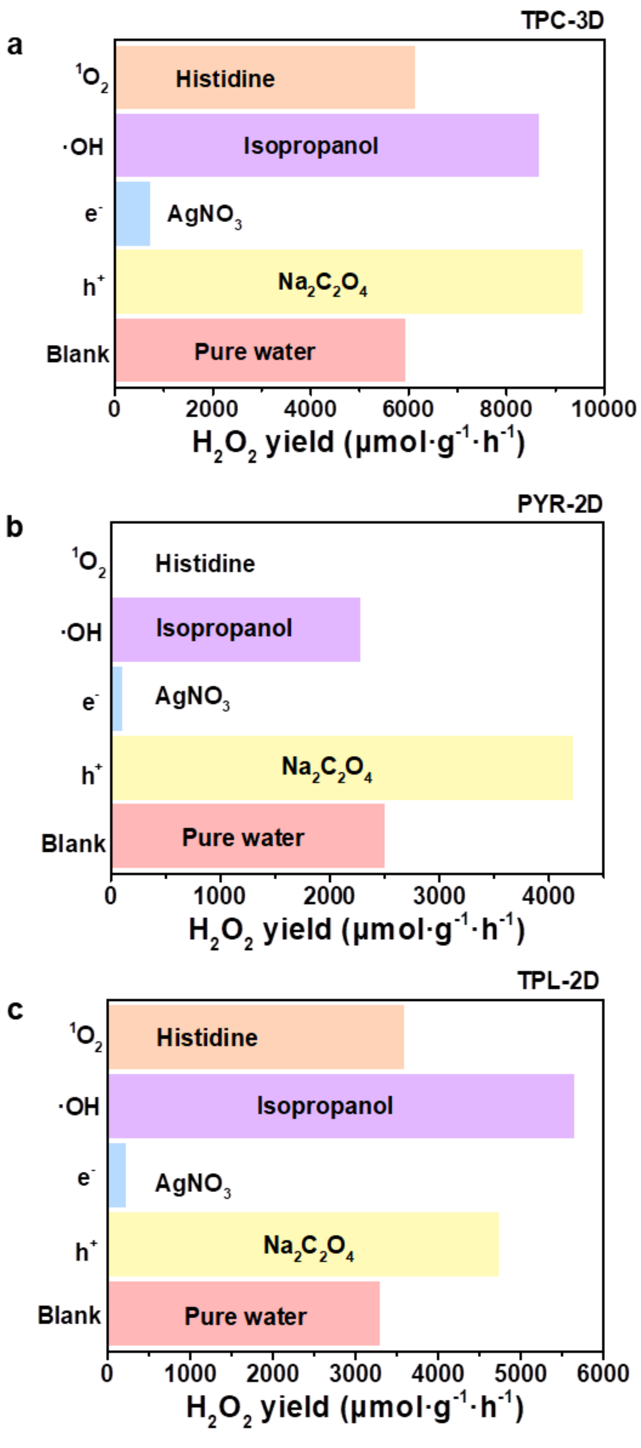


**Supplementary Fig. 21 | The effects of reactive intermediate species on hydrogen peroxide production. a–c**, The $H_2O_2$ production by (**a**) TPC-3D, (**b**) PYR-2D, (**c**) TPL-2D upon the addition of different sacrificial agents.

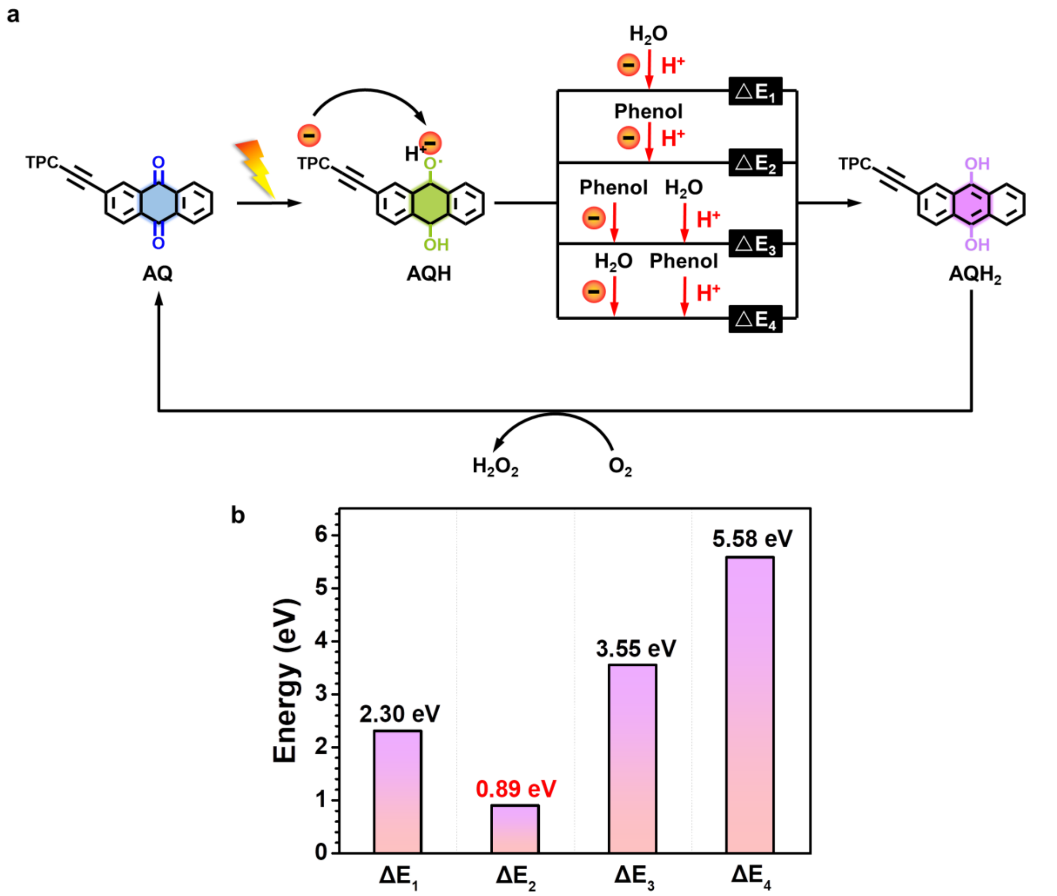


**Supplementary Fig. 22 | Investigation of proton source and electron source. a**, Schematic diagram of proton and electron sources in the ORR reaction of AQ in the presence of phenol. **b**, Reaction energies for the generation of anthraquinones from AQH via four different proton, electron source pathways discussed in Supplementary Fig. 22a.

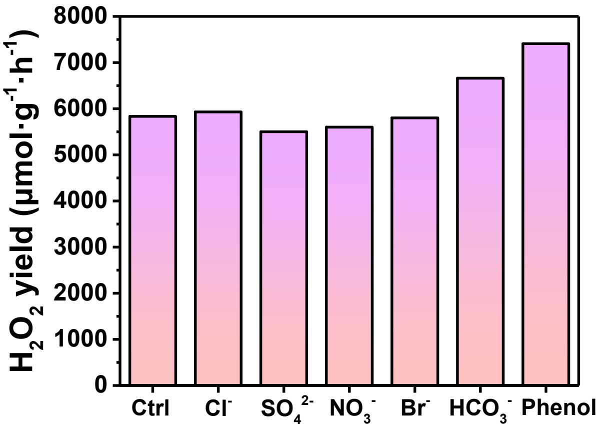


**Supplementary Fig. 23 | The withstand interference ability test.** Effect of water matrix components (coexisting anions) and dissolved organic matters (phenol as an example) on the photocatalytic performance of TPC-3D. Reaction conditions were: [photocatalyst] = 0.02 g·$L^{-1}$, [anions] = 5 mM, [phenol] = 5 ppm.

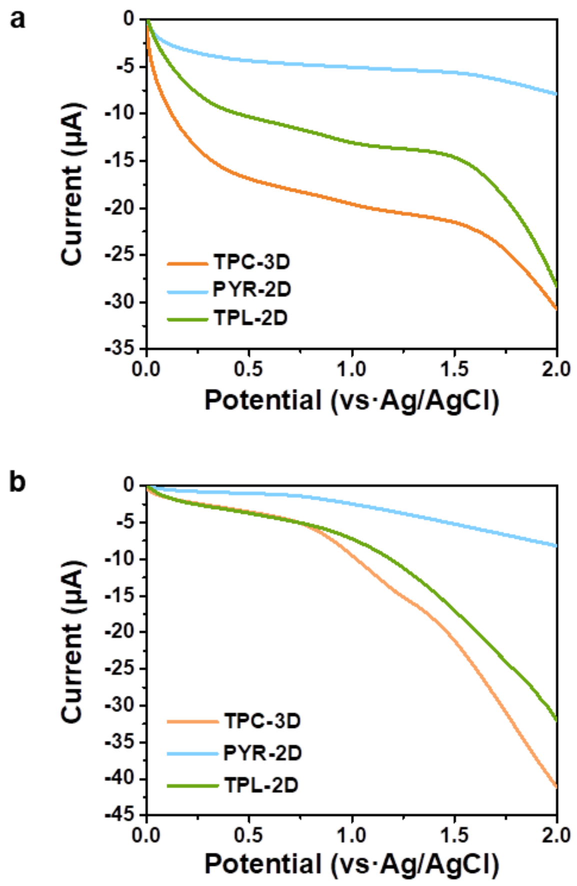


**Supplementary Fig. 24 | Water oxidation half-reaction in the photosynthesis of $H_2O_2$.** The ring currents of Rotating Ring-Disk Electrodes (RRDE) polarization curves at 1600 rpm in 0.1 M phosphate buffer (pH=7) by bubbling Ar gas. **a,** The potential of Pt ring electrodes was set at -0.23 V versus Ag/AgCl to detect $O_2$. **b**, The potential of Pt ring electrodes was set at +0.6 V versus Ag/AgCl to detect $H_2O_2$. TPC-3D, PYR-2D and TPL-2D exhibited significant reduction currents when the applied potential at the Pt ring electrode was -0.23 V and +0.6V, which were attributed to the reduction of $O_2$ and $H_2O_2$, respectively. Both the reduction currents for $O_2$ and $H_2O_2$ were improved in TPC-3D. Moreover, it could be concluded that all the CPs preferred to generate $O_2$ rather than $H_2O_2$ from WOR, as the currents were larger when the applied electrode potential was -0.23 V.

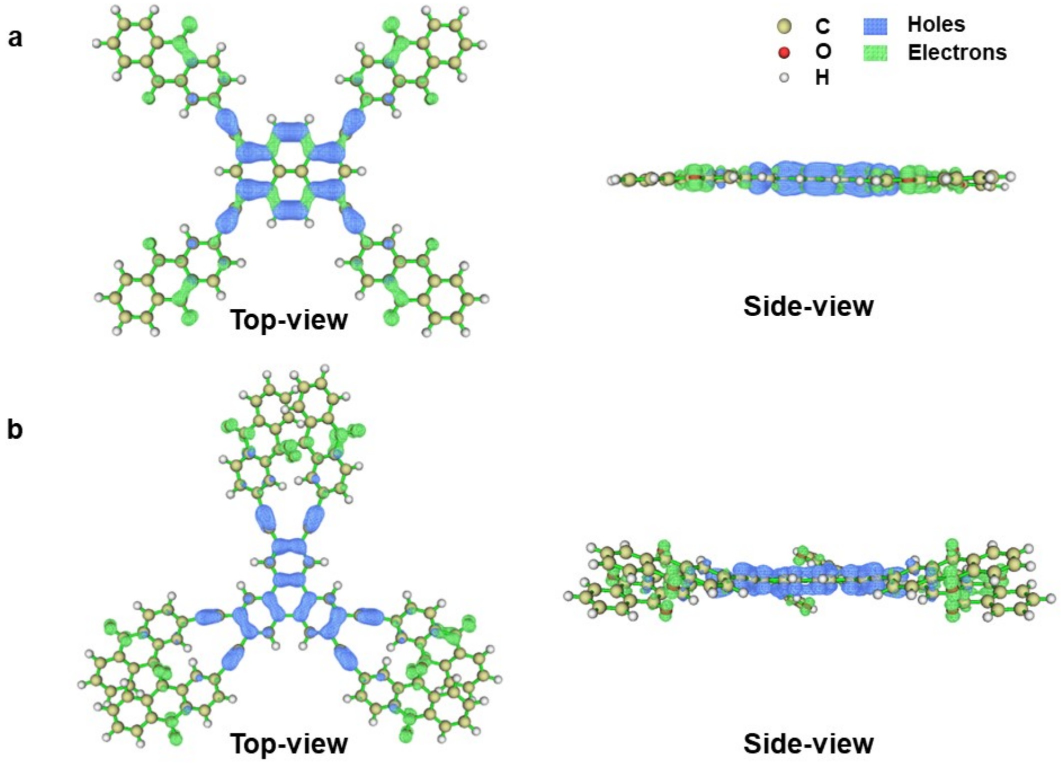


**Supplementary Fig. 25 | Time-dependent density functional theory (TD-DFT) calculation. a–b**, The analysis for the distribution of the holes (blue) and electrons (green) for (**a**) PYR-2D, (**b**) TPL-2D through TD-DFT (Isosurface value=0.001).

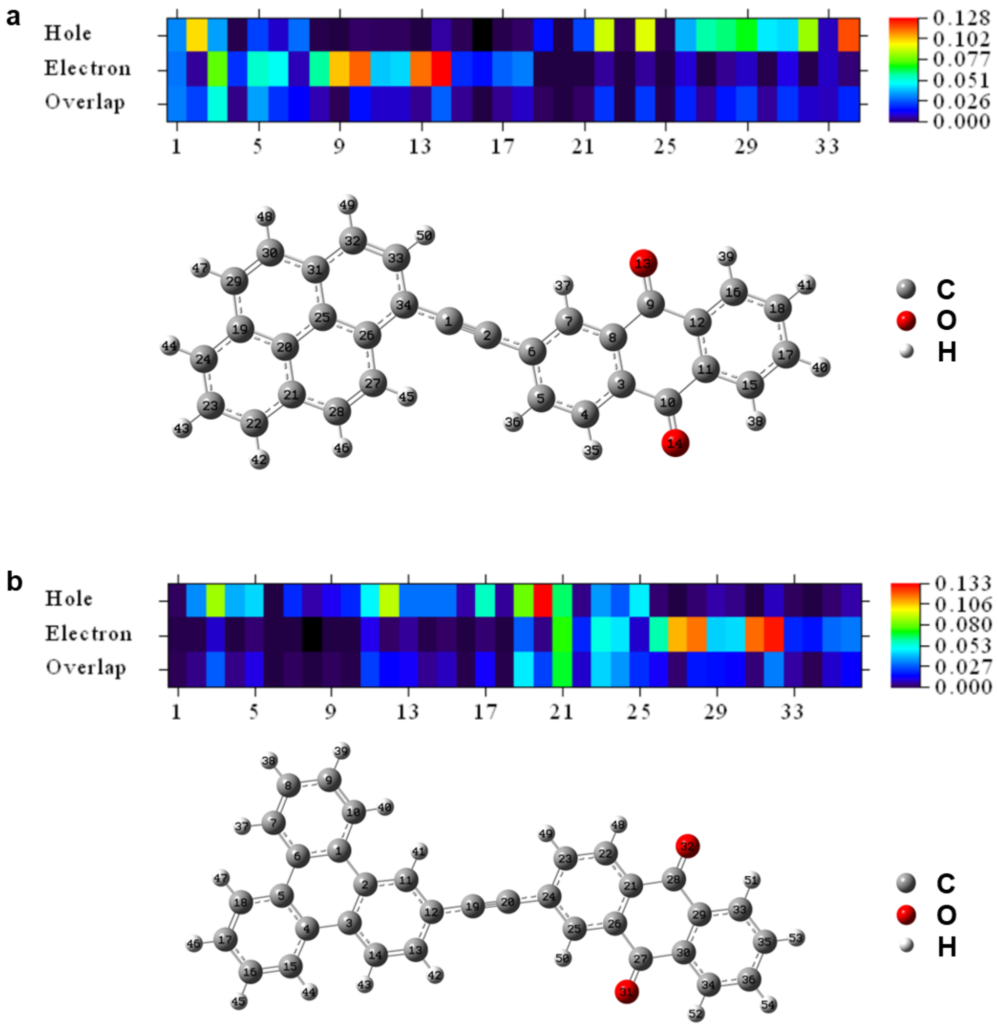


**Supplementary Fig. 26 | The distribution of electrons and holes. a–b** The contribution of non-hydrogen atoms to holes and electrons in excited state and the corresponding atom labels of (**a**) PYR-2D and (**b**) TPL-2D.

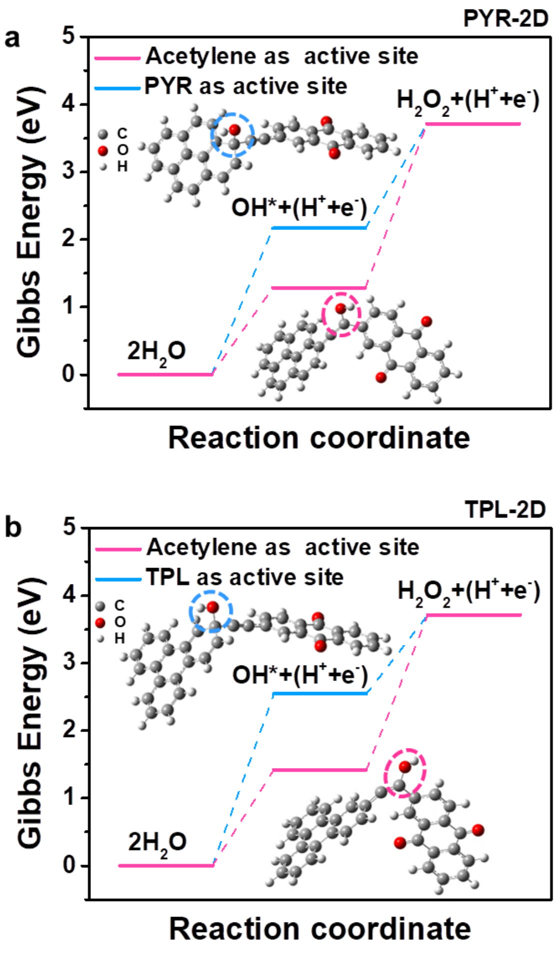


**Supplementary Fig. 27 | Investigation of active sites for water oxidation reaction (WOR) through free energy diagrams.** Calculated free energy diagrams of two-electron water oxidation pathways toward $H_2O_2$ production on acetylene and electron-donor sites in (**a**) PYR-2D and (**b**) TPL-2D. Inset: the adsorption configuration of OH* on different sites.

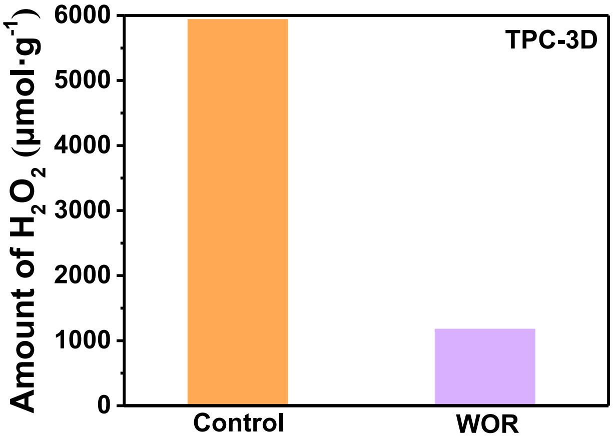


**Supplementary Fig. 28 | $H_2O_2$ production through water oxidation reaction (WOR).** The amount of $H_2O_2$ production under the conditions of bubbling argon gas to exclude $O_2$ from the air and adding electron sacrificial agents to inhibit the generation of $H_2O_2$ from oxygen reduction.

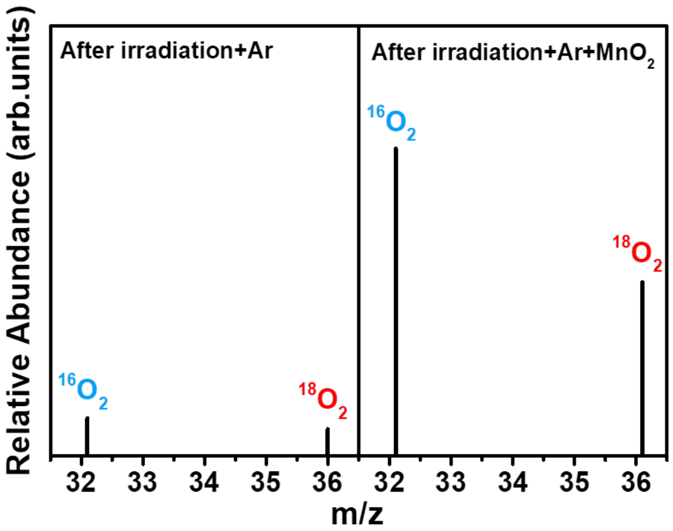


**Supplementary Fig. 29 | $H_2{}^{18}O$ isotope experiment to explore the $H_2O_2$ evolution through water oxidation reaction (WOR) pathway for TPC-3D**. Before the addition of $MnO_2$, there were weak $^{18}O_2$ and $^{16}O_2$ signals in the system were weak, which was attributed to trace of $O_2$ from the reaction environment. It was evident that, when isotopically labeled water ($H_2{}^{18}O$) was employed in the photocatalytic reaction instead of conventional water ($H_2{}^{16}O$), and the subsequently generated hydrogen peroxide was converted to $O_2$ by $MnO_2$, a distinct enhancement of the isotopic signal peak corresponding to $^{18}O_2$ was observed. This observation effectively demonstrated the evolution of $H_2O_2$ by hole-induced WOR pathway.

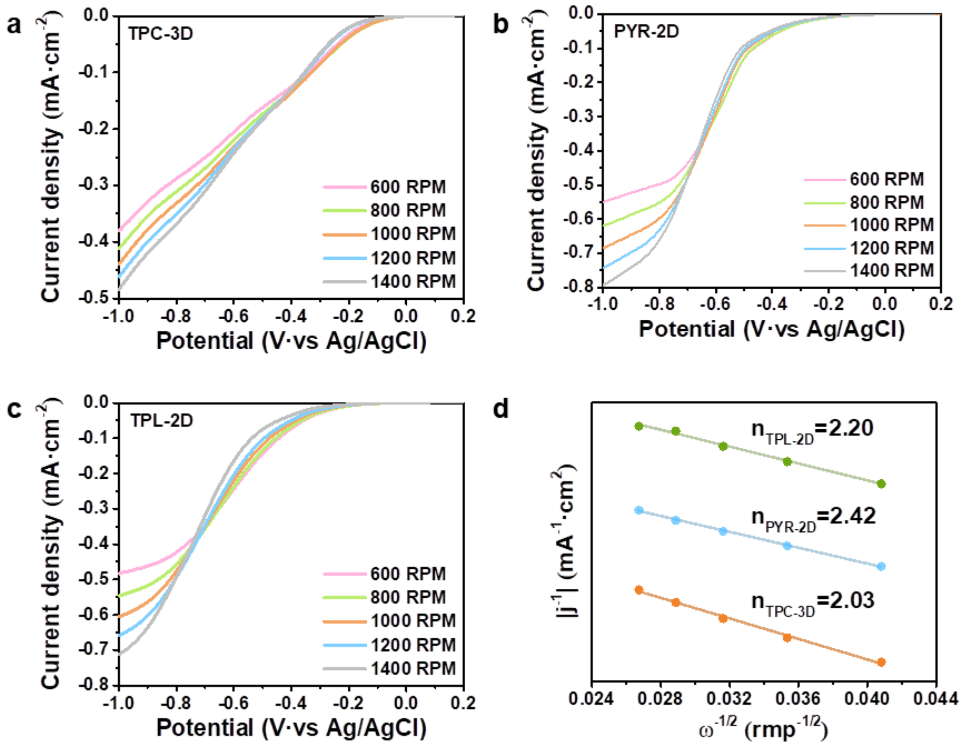


**Supplementary Fig. 30 | Investigation of electron transfer numbers in oxygen reduction reaction (ORR). a–d**, Linear sweep voltammetry (LSV) curves of (**a**) TPC-3D, (**b**) PYR-2D, (**c**) TPL-2D, and (**d**) Koutecky-Levich plots (at -0.6 V vs. Ag/AgCl) for TPC-3D, PYR-2D, TPL-2D respectively. All CPs exhibited effective two-electrons oxygen reduction to produce $H_2O_2$.

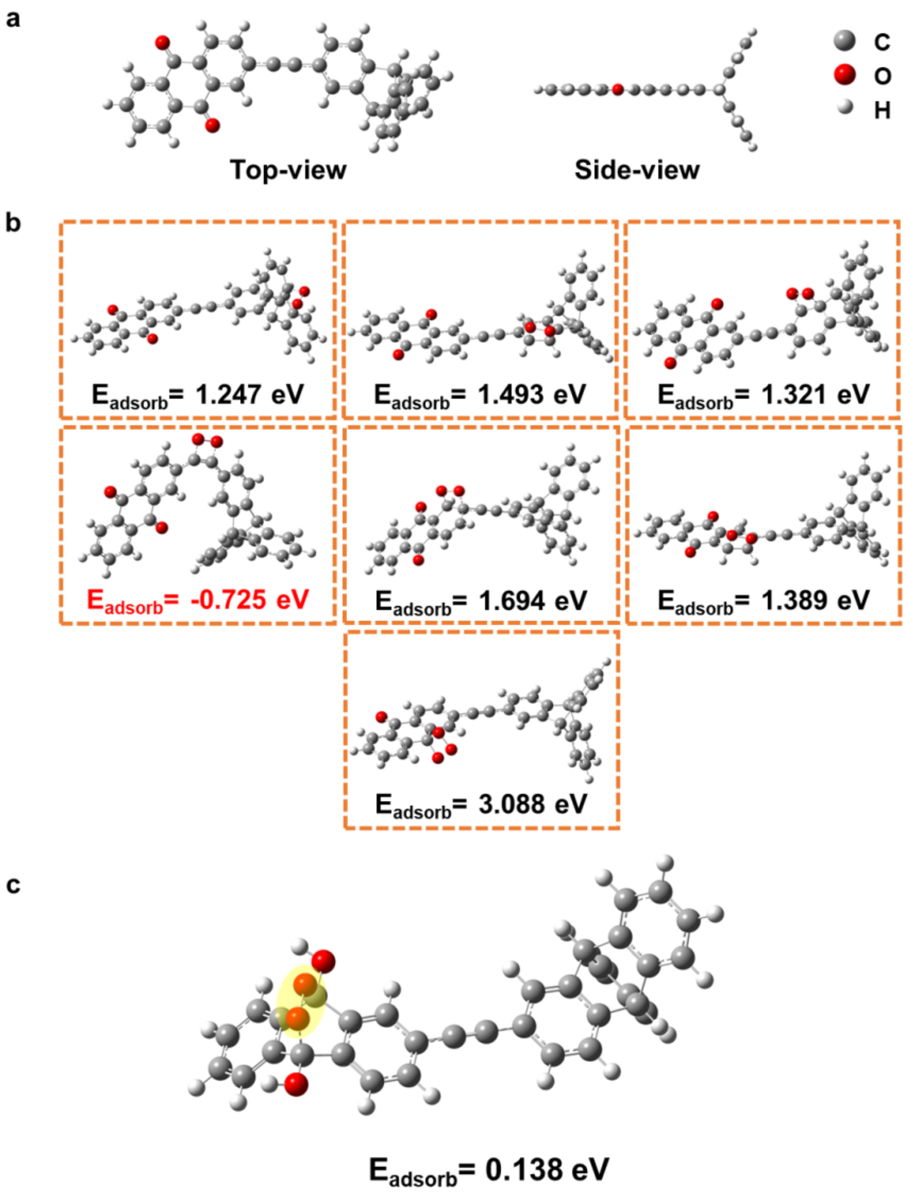


**Supplementary Fig. 31 | $O_2$ adsorption sites and adsorption energy for TPC-3D.** **a**, The molecular model of TPC-3D before oxygen adsorption. **b**, The adsorption energy when oxygen is adsorbed in different positions. An alternative oxygen reduction pathway was proposed through DFT calculations, revealing that $O_2$ predominantly chemisorbs onto the alkynyl moieties to form endoperoxide species in TPC-3D, rather than on the AQ or triptycene (TPC) moieties. **c**, Molecular model and adsorption energy ($E_{adsorb}$) for excited TPC-3D when oxygen is adsorbed at $AQH_2$ moieties.

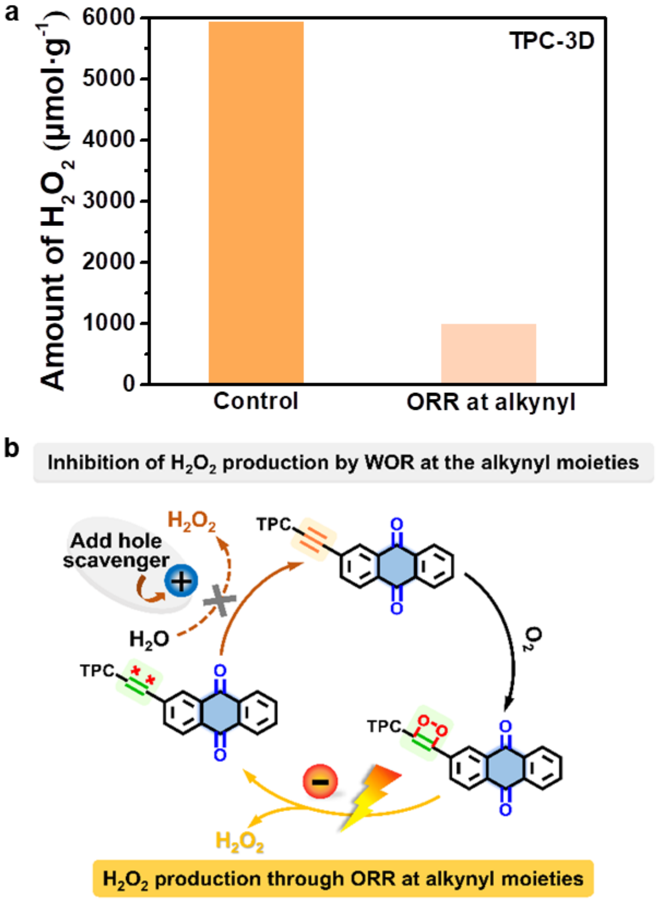


**Supplementary Fig. 32 | $H_2O_2$ production through oxygen reduction reaction (ORR) at alkynyl moieties. a**, The amount of $H_2O_2$ production under the conditions that oxygen was injected first, then bubbled argon gas to exclude $O_2$ from the air and adding hole sacrificial agents to inhibit the generation of $O_2$ from water oxidation. **b**, Elucidation of the mechanistic aspects of the ORR at alkynyl moieties experimental processes. This portion of $O_2$ was proven to be capable of converting into $H_2O_2$, as $H_2O_2$ was successfully produced in Ar atmosphere and with hole sacrificial agents added to exclude $O_2$ from the air and inhibit the generation of $O_2$ from water oxidation.

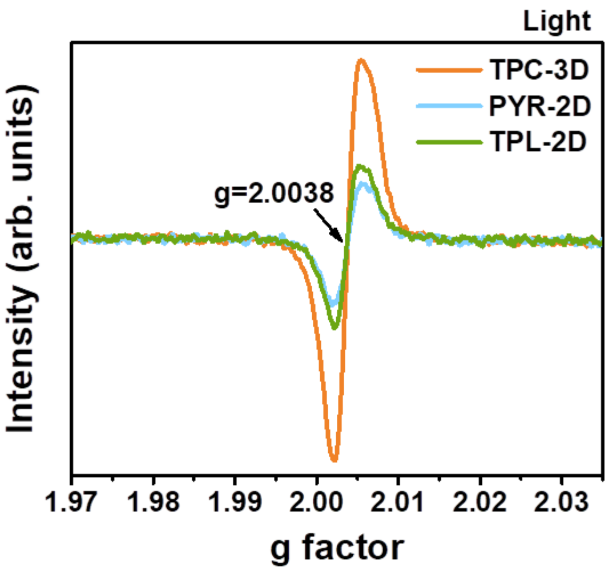


**Supplementary Fig. 33 | Electron paramagnetic resonance (EPR) spectra of CPs** under illumination for 1 h. The EPR signal was at g=2.0038, indicating that the radicals were mainly formed on the O atoms in the quinone groups, further elucidating the role of AQ in electron storage. In addition, the strongest free radical signal of TPC-3D illustrates its electron storage.

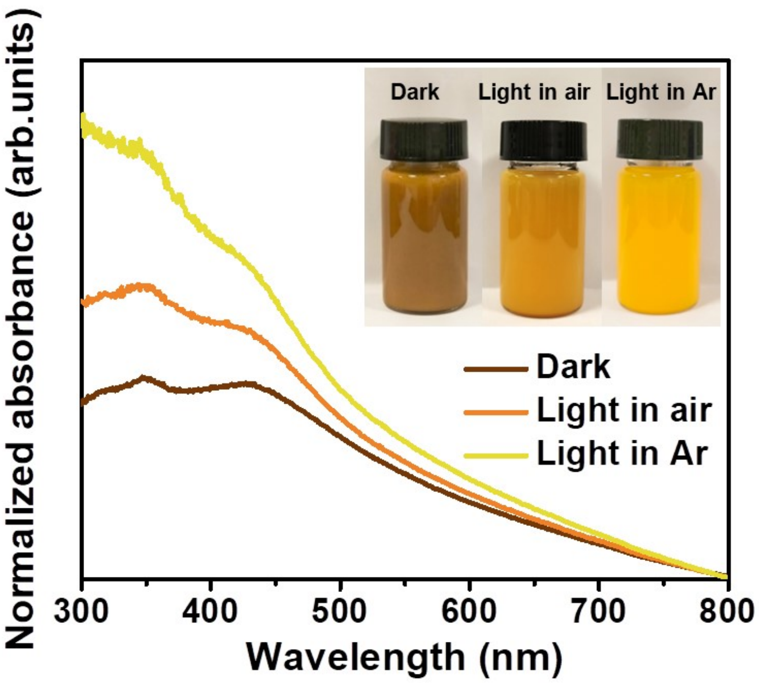


**Supplementary Fig. 34 | Absorption spectra of TPC-3D samples before and after illumination in argon and air.** The corresponding absorption spectra due to light illumination. Inset: a photograph of TPC-3D before illumination and after illumination under air and argon.

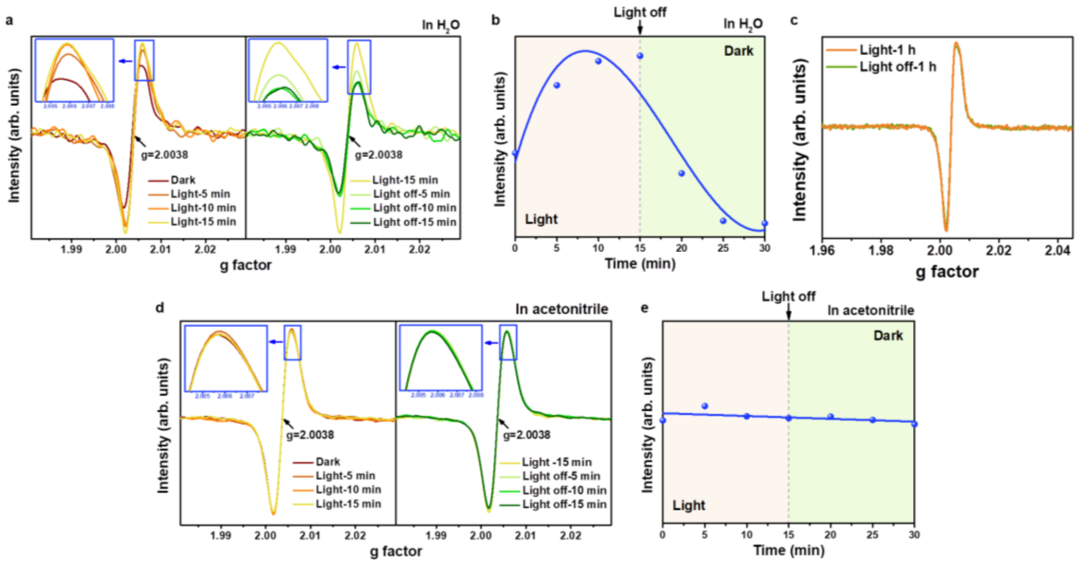


**Supplementary Fig. 35 | Electron paramagnetic resonance (EPR) spectra form light to dark. a**, The time-dependent in-situ EPR spectra of TPC-3D in $H_2O$. Inset: local magnification of the EPR highest point (g = 2.0058) in $H_2O$. **b**, Fitted curve of the highest point of the EPR spectrum in $H_2O$ (g = 2.0058) versus time. **c**, The time-dependent in-situ EPR spectra of TPC-3D in air. **d**, The time-dependent in-situ EPR spectra of TPC-3D in SafeDry acetonitrile. Inset: local magnification of the EPR highest point (g = 2.0058) in SafeDry acetonitrile.**e**, Fitted curve of the highest point of the EPR spectrum in SafeDry acetonitrile (g = 2.0058) versus time.

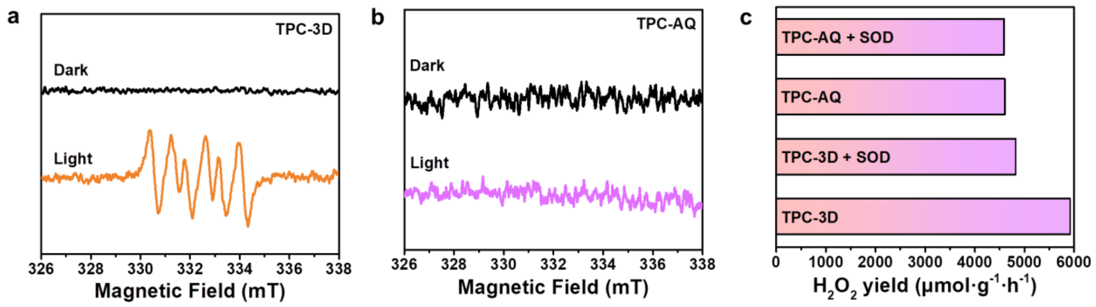


**Supplementary Fig. 36 | Elucidation of the oxygen reduction reaction (ORR) pathway at different sites. a–b**, Electron paramagnetic resonance (EPR) spectra of (**a**) TPC-3D and (**b**) TPC-AQ by using 5,5-dimethyl-1-pyrroline N-oxide (DMPO) as a scavenger in $O_2$ atmosphere under visible light irradiation to examine the existence of $\cdot O_2^-$ during ORR. **c**, $H_2O_2$ yield of TPC-3D and TPC-AQ before and after adding SOD.

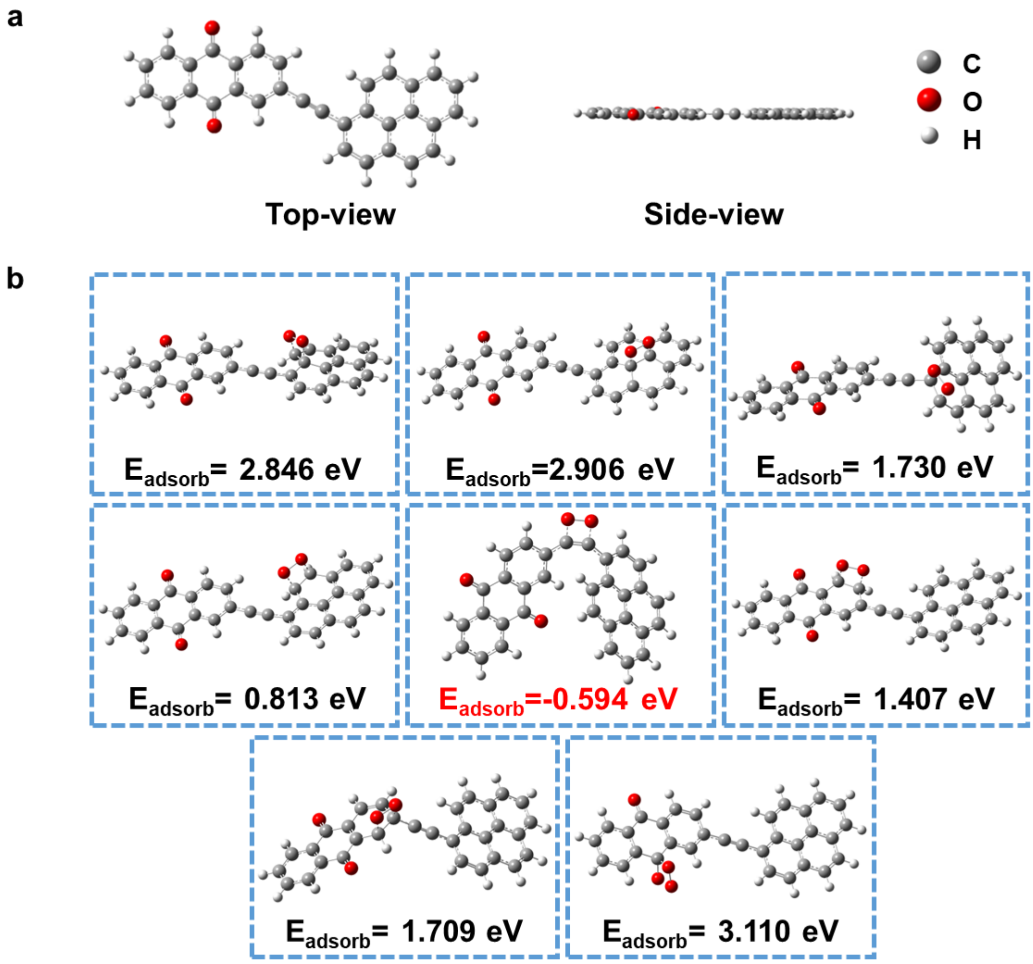


**Supplementary Fig. 37 | $O_2$ adsorption sites and adsorption energy for PYR-2D.** **a**, The molecular model of PYR-2D before oxygen adsorption. **b**, The adsorption energy when oxygen is adsorbed in different positions. DFT calculations revealed that $O_2$ predominantly chemisorbs onto the alkynyl moieties to form the endoperoxide species in PYR-2D.

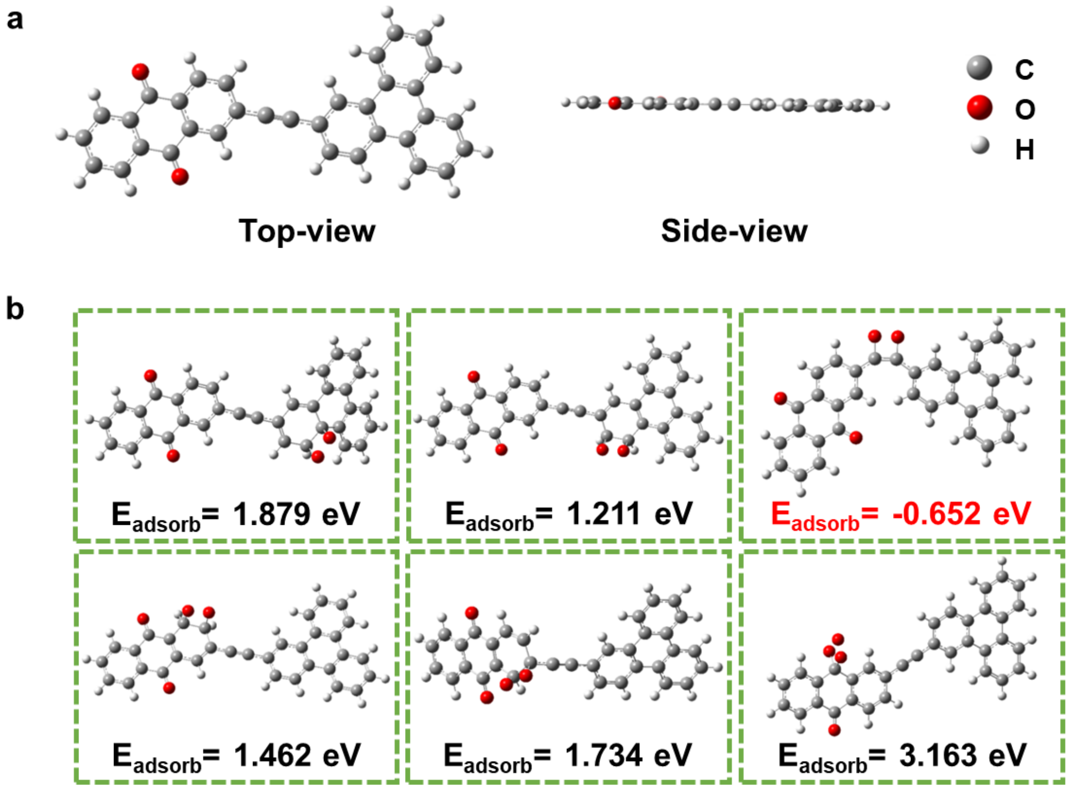


**Supplementary Fig. 38 | $O_2$ adsorption sites and adsorption energy for TPL-2D. a**, The molecular model of TPL-2D before oxygen adsorption. **b**, The adsorption energy when oxygen is adsorbed in different positions. DFT calculations revealed that $O_2$ was dominantly chemisorbed on the alkynyl moieties to form the endoperoxide species in TPL-2D.

a

b

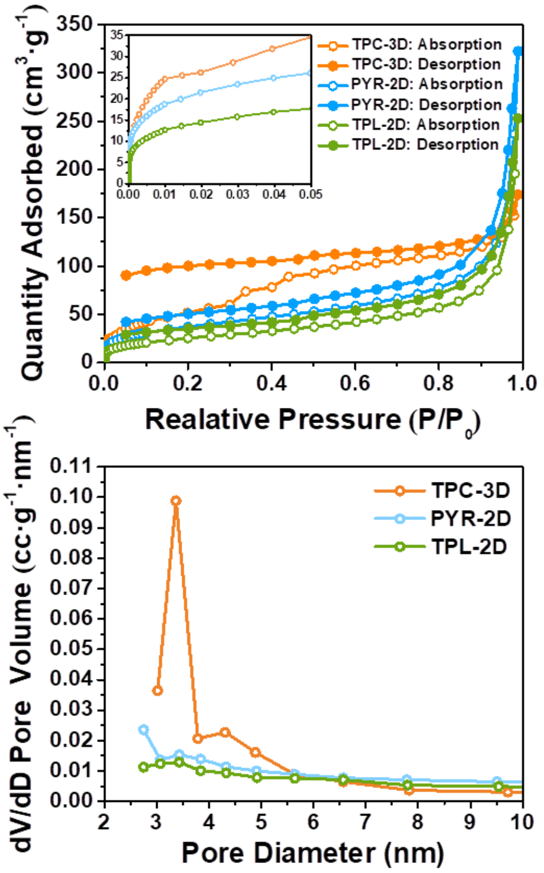


c

| | TPC-3D | PYR-2D | TPL-2D |
|---|---|---|---|
| BET Surface ($m^2 \cdot g^{-1}$) | 193.32 | 136.17 | 92.39 |
| Most Probable Pore Diameter (nm) | 3.37 | 2.75 | 3.43 |

**Supplementary Fig. 39 | Measurements of Brunauer-Emmett-Teller (BET) surface areas. a**, $N_2$ adsorption-desorption isotherms of CPs. **b**, The corresponding pore size distributions of CPs. Inset: local magnification of the $N_2$ adsorption-desorption isotherms (relative pressure range 0–0.05). **c**, The corresponding BET surface and most probable pore diameter of CPs. TPC-3D possessed a specific surface area 40%–50% larger than that of PYR-2D and TPL-2D, owing to its 3D structure.

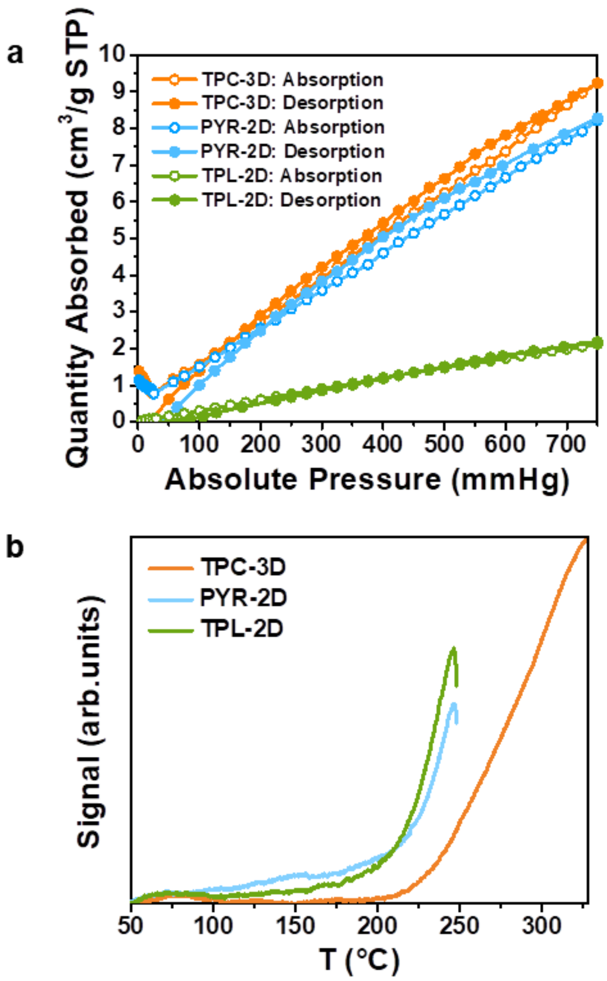


**Supplementary Fig. 40 | Physical and chemical adsorption capacity of CPs for $O_2$.**

**a**, The physical adsorption of $O_2$. **b**, $O_2$-TPD spectra of the CPs.

**a**

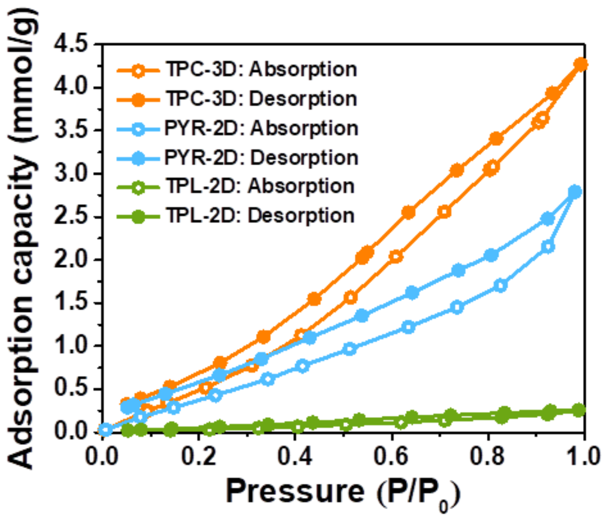


**b**

| | TPC-3D | PYR-2D | TPL-2D |
|---|---|---|---|
| BET Surface ($m^2 \cdot g^{-1}$) | 67.69 | 43.47 | 4.58 |

**Supplementary Fig. 41 | Water vapor adsorption of CPs at room temperature. a**, Water vapor adsorption-desorption isotherms of CPs. TPC-3D exhibits the highest adsorption capacity for water vapor, allowing water to permeate into the pores for water oxidation reactions. **b**, The corresponding BET surface area of CPs. TPC-3D possessed the largest BET surface area for water vapor adsorption. On the other hand, the adsorption curves had no obvious trend of saturation adsorption, indicating irregular pore structure of CPs.

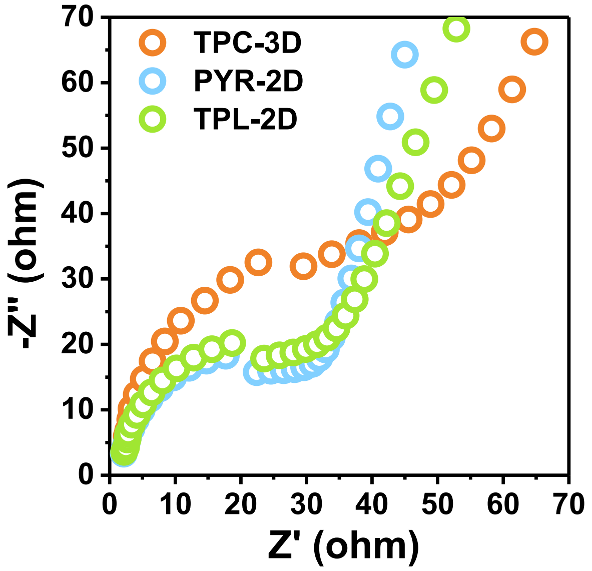


**Supplementary Fig. 42 | Electrochemical impedance spectra (EIS) of CPs.** The electrical resistance of TPC-3D was significantly higher than that of PYR-2D and TPL-2D, indicating that the charges transportation in the two-dimensional catalysts was more fluent.

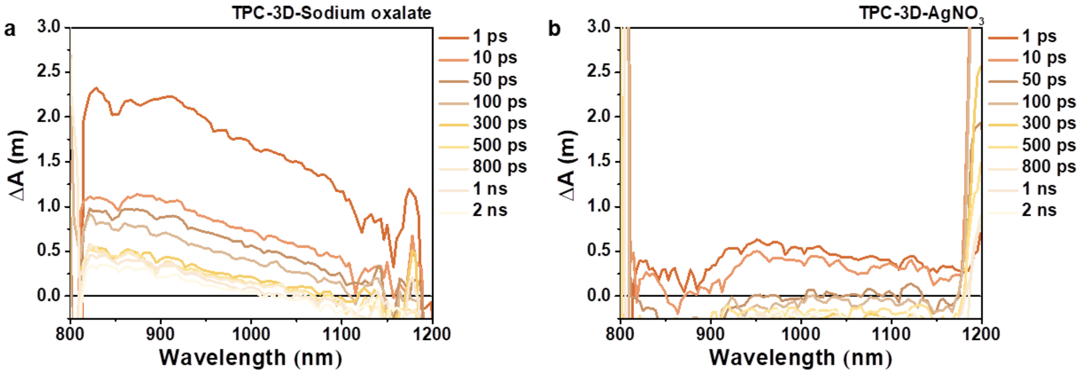


**Supplementary Fig. 43 | Femtosecond time-resolved transient absorption (TA) measurements in the near-infrared region of TPC-3D. a–b**, Femtosecond TA spectra in 800–1200 nm of (**a**) TPC-3D with sodium oxalate (10 mM) and (**b**) TPC-3D with $AgNO_3$ (10 mM). In comparison with the signal strength in Fig. 6a, the hole sacrificial agent (sodium oxalate) had no significant effect on the signal, while the signal decreased substantially after adding the electron sacrificial agent ($AgNO_3$), which proved that the signal may be attributed to the electrons.

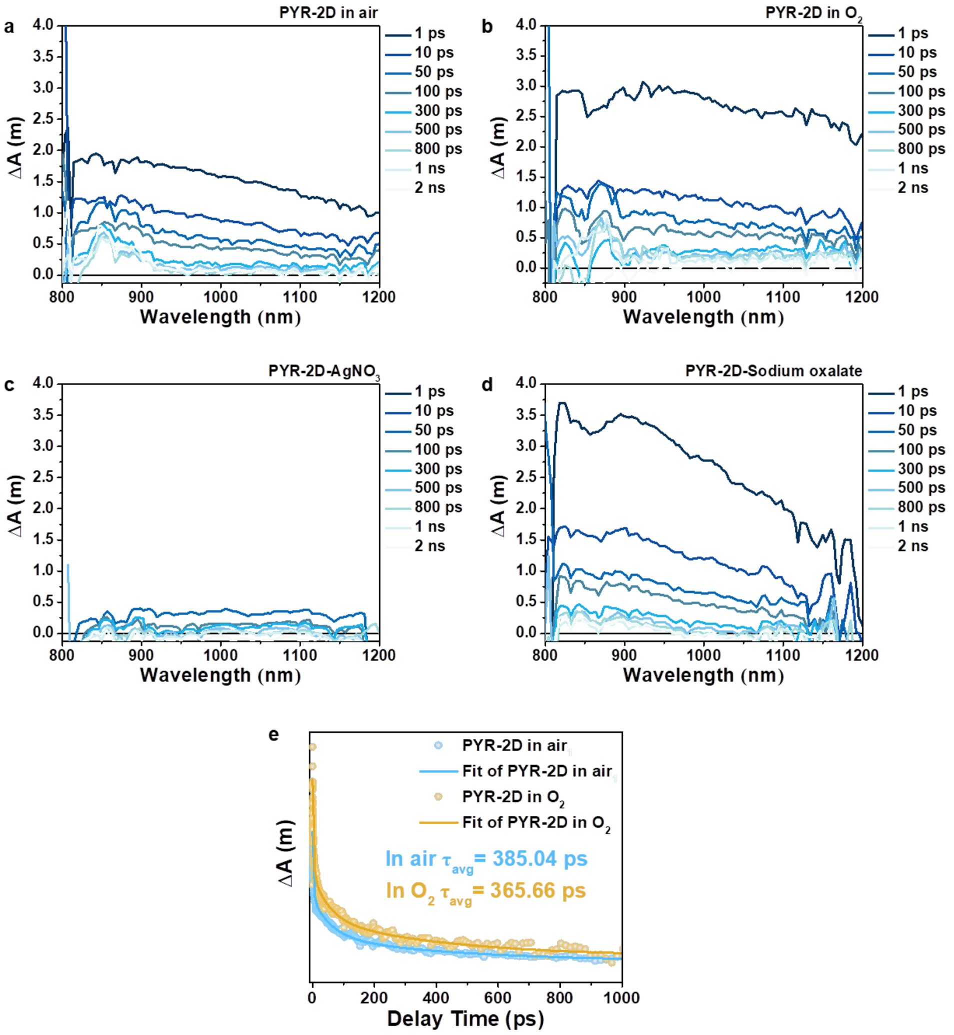


**Supplementary Fig. 44 | Femtosecond time-resolved transient absorption (TA) measurements in the near-infrared region of PYR-2D. a–d**, Femtosecond TA spectra in the 800–1200 nm range of (**a**) PYR-2D in air, (**b**) PYR-2D in $O_2$, (**c**) PYR-2D with sodium oxalate (10 mM), (**d**) PYR-2D with $AgNO_3$ (10 mM). The signal increased when the hole sacrificial agent (sodium oxalate) was added and decreased substantially after adding the electron sacrificial agent ($AgNO_3$), which proved that the signal may be attributed to the electrons. Furthermore, it could be presumed that the 2-fold increase in signal was due to the addition of the hole sacrificial agent that reduced the recombination of photogenerated charge carriers. **e**, Comparison of TA kinetic profiles at 1000 nm under air and $O_2$ conditions, respectively. The specific values of decay lifetimes fitted by the tri-exponential function are shown in Fig. 6d.

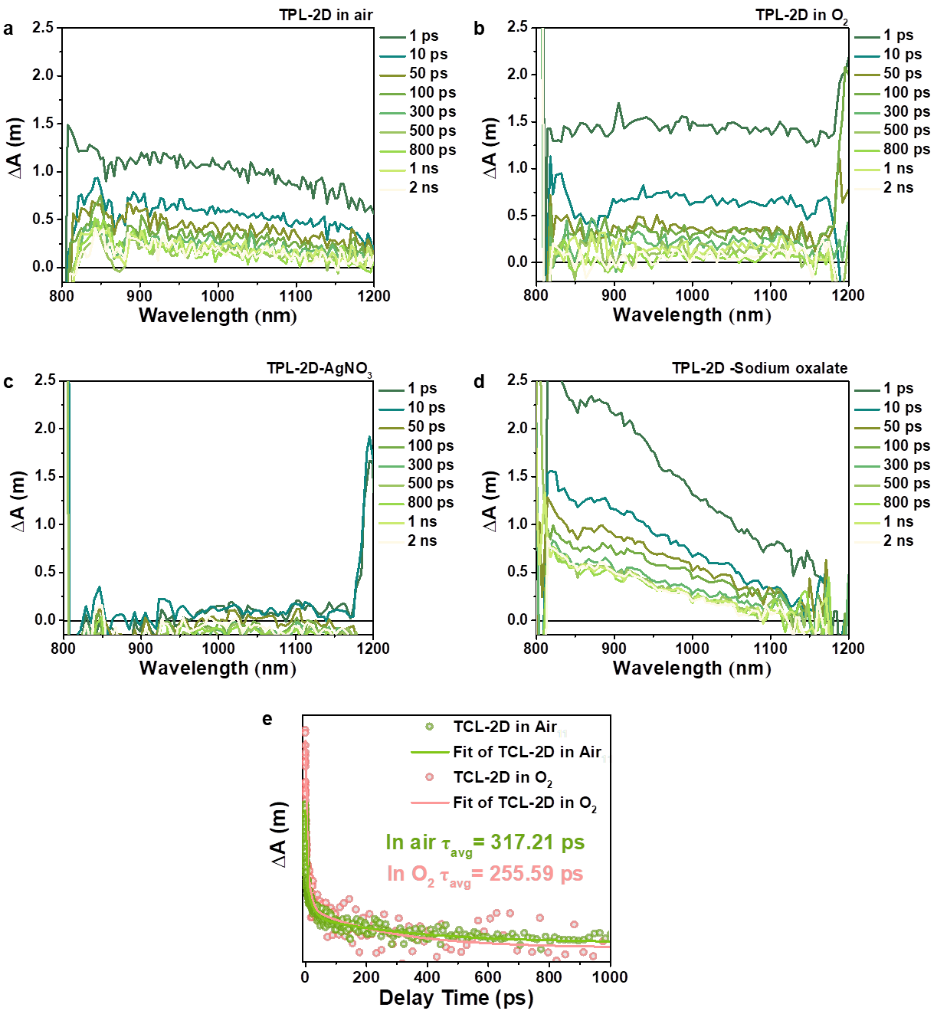


**Supplementary Fig. 45 | Femtosecond time-resolved transient absorption (TA) measurements in the near-infrared region of TPL-2D. a–d**, Femtosecond TA spectra in the 800–1200 nm range of (**a**) TPL-2D in air, (**b**) TPL-2D in $O_2$, (**c**) TPL-2D with $AgNO_3$ (10 mM), (**d**) TPL-2D with $AgNO_3$ (10 mM). The signal obviously increased when the hole sacrificial agent (sodium oxalate) was added and decreased substantially after adding the electron sacrificial agent ($AgNO_3$), which proved that the signal may be attributed to the electrons. In addition, it could be speculated that the 2-fold increase of signal was due to the reduction of photogenerated charge carriers recombination by the addition of the hole sacrifice agent. **e**, Comparison of TA kinetic profiles at 1000 nm under air and $O_2$ conditions, respectively. The specific values of decay lifetimes fitted by the tri-exponential function are shown in Fig. 6d.

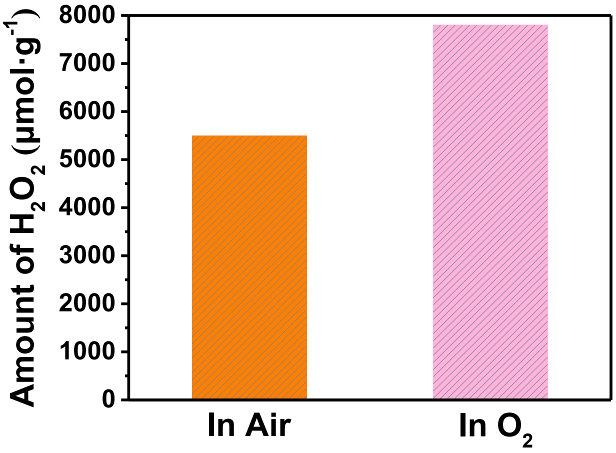


**Supplementary Fig. 46 | The photosynthetic performance of TPC-3D for $H_2O_2$ production in different atmospheres.** There was a nearly 50% increase in the photosynthetic rate of $H_2O_2$ in the presence of $O_2$ compared to that in air. Additionally, when considering the average decay lifetimes for TPC-3D, they were accelerated from 191.6 ps in air to 79.5 ps in the presence of $O_2$ (as shown in Figure 6c). This acceleration could be attributed, at least in part, to the injection of photoinduced electrons into $O_2$, which were utilized to generate $H_2O_2$.

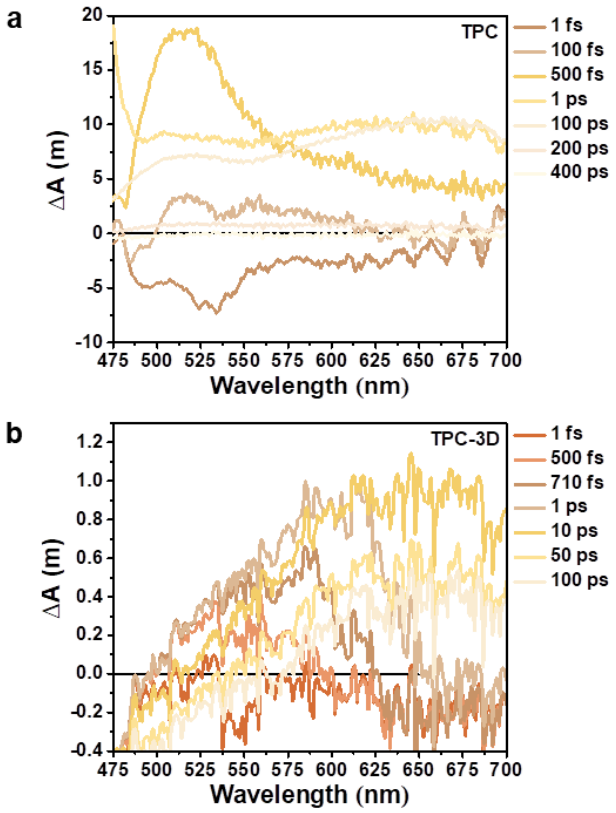


**Supplementary Fig. 47 | Femtosecond time-resolved transient absorption (TA) measurements of TPC-3D in the visible region on femtosecond (fs) and sub-femtosecond (sub-fs) scales. a–b**, The TA spectra of (**a**) pure TPC monomer and (**b**) TPC-3D on fs and sub-fs scales. TPC-3D exhibited a positive peak consistent with their electron donor monomers (TPC) at ~515 nm, which was defined as the signal of excitons. For TPC-3D, the excitons peak at ~515 nm was followed by a positive peak at 645 nm, which was attributed to polarons, and the time delay between them represented the time of excitons transferring to polarons, i.e. 630 fs (TA kinetic profiles of TPC-3D at 515 nm and 645 nm are showed in Fig. 8c).

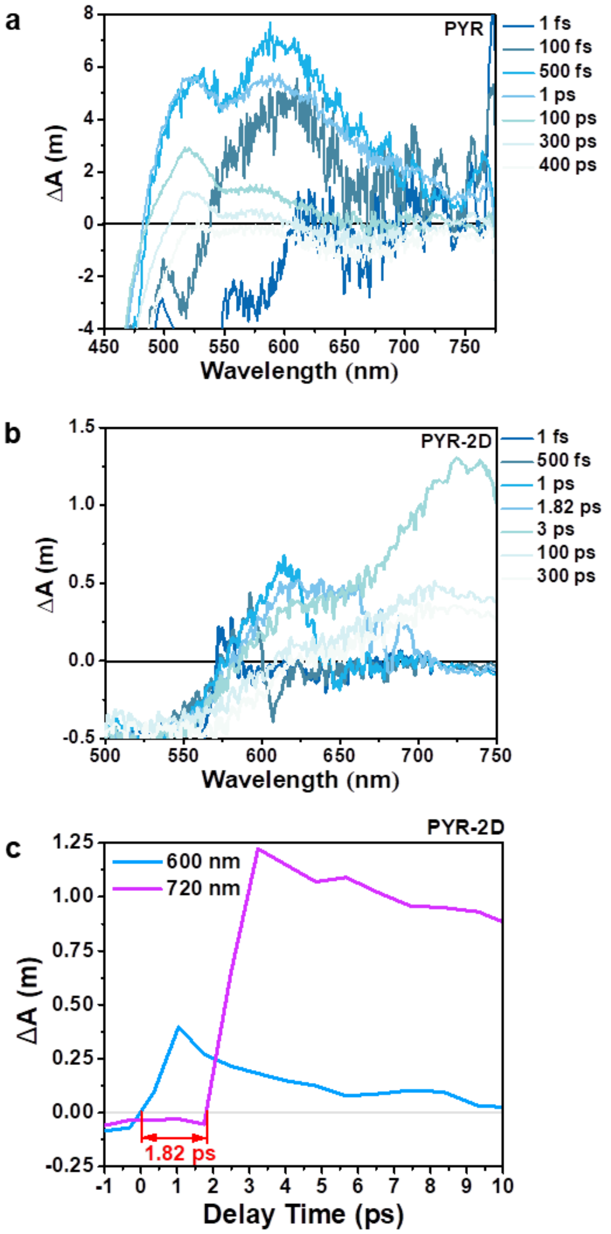


**Supplementary Fig. 48 | Femtosecond time-resolved transient absorption (TA) measurements of PYR-2D in the visible region on fs and sub-fs scales. a–b**, The TA spectra of (**a**) pure PYR monomer (**b**) and PYR-2D on fs and sub-fs scales. **c**, TA kinetic profiles of PYR-2D at 600 nm and 720 nm. PYR-2D exhibited a positive peak consistent with their electron donor monomers (PYR) at ~600 nm, which was defined as the signal of excitons. For PYR-2D, the excitons peak at ~600 nm was followed by a positive peak attributed to the polarons at 720 nm, and the time delay between them represented the time of excitons transferring to polarons, which was 1.82 ps.

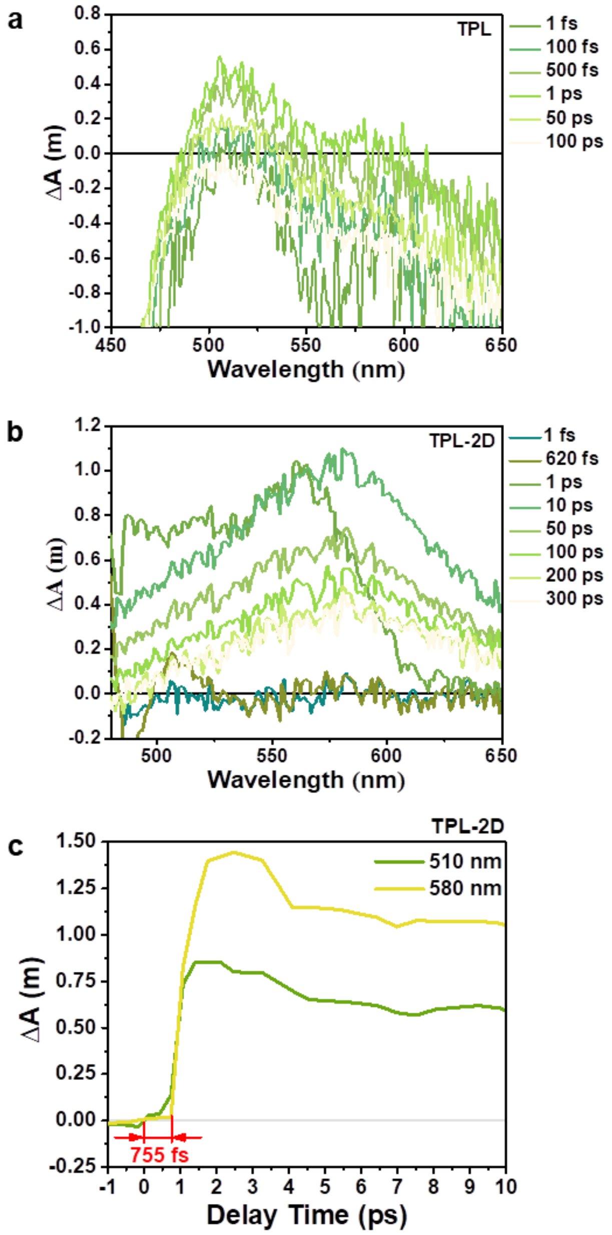


**Supplementary Fig. 49 | Femtosecond time-resolved transient absorption (TA) measurements of TPL-2D in the visible region on fs and sub-fs scales. a–b**, The TA spectra of (**a**) pure TPL monomer (**b**) and TPL-2D on fs and sub-fs scales. **c**, TA kinetic profiles of TPL-2D in 510 nm and 580 nm. TPL-2D exhibited a positive peak consistent with their pure electron donor monomers (TPL) at ~510 nm, which was defined as the signal of excitons. For TPL-2D, the excitons peak at ~510 nm was followed by a positive peak attributed to the polarons at 580 nm, and the time delay between them represented the time of excitons transferring to polarons, i.e. 755 fs.

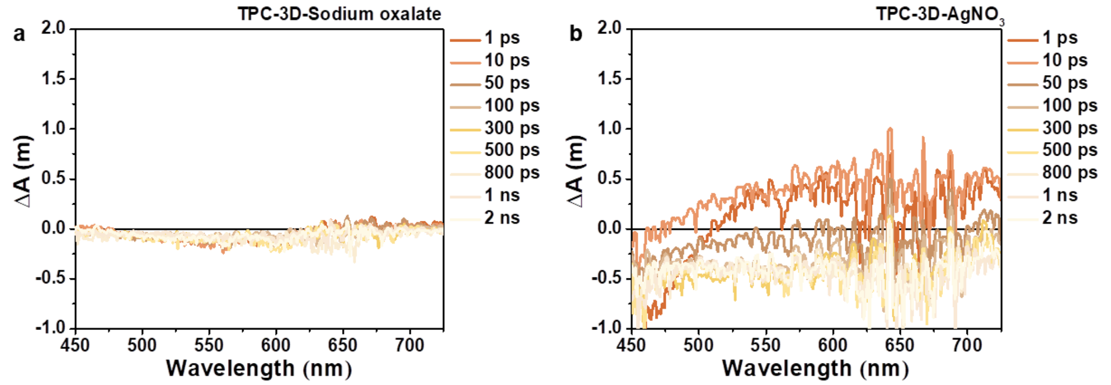


**Supplementary Fig. 50 | Femtosecond time-resolved transient absorption (TA) measurements of TPC-3D in the visible region on picosecond (ps) and nanosecond (ns) scales. a–b**, The TA spectra in the 450–780 nm range of (**a**) TPC-3D with sodium oxalate (10 mM), (**b**) TPC-3D with $AgNO_3$ (10 mM) on ps and ns scales. Compare with the signal strength in Fig. 8a, signals in Supplementary Fig. 50 were drastically reduced regardless of adding hole sacrificial agents (sodium oxalate) or electron sacrificial agents ($AgNO_3$), indicating proved that the signal might be attributed to polarons (the pairs of more loosely bound charges by Coulomb attraction forces).

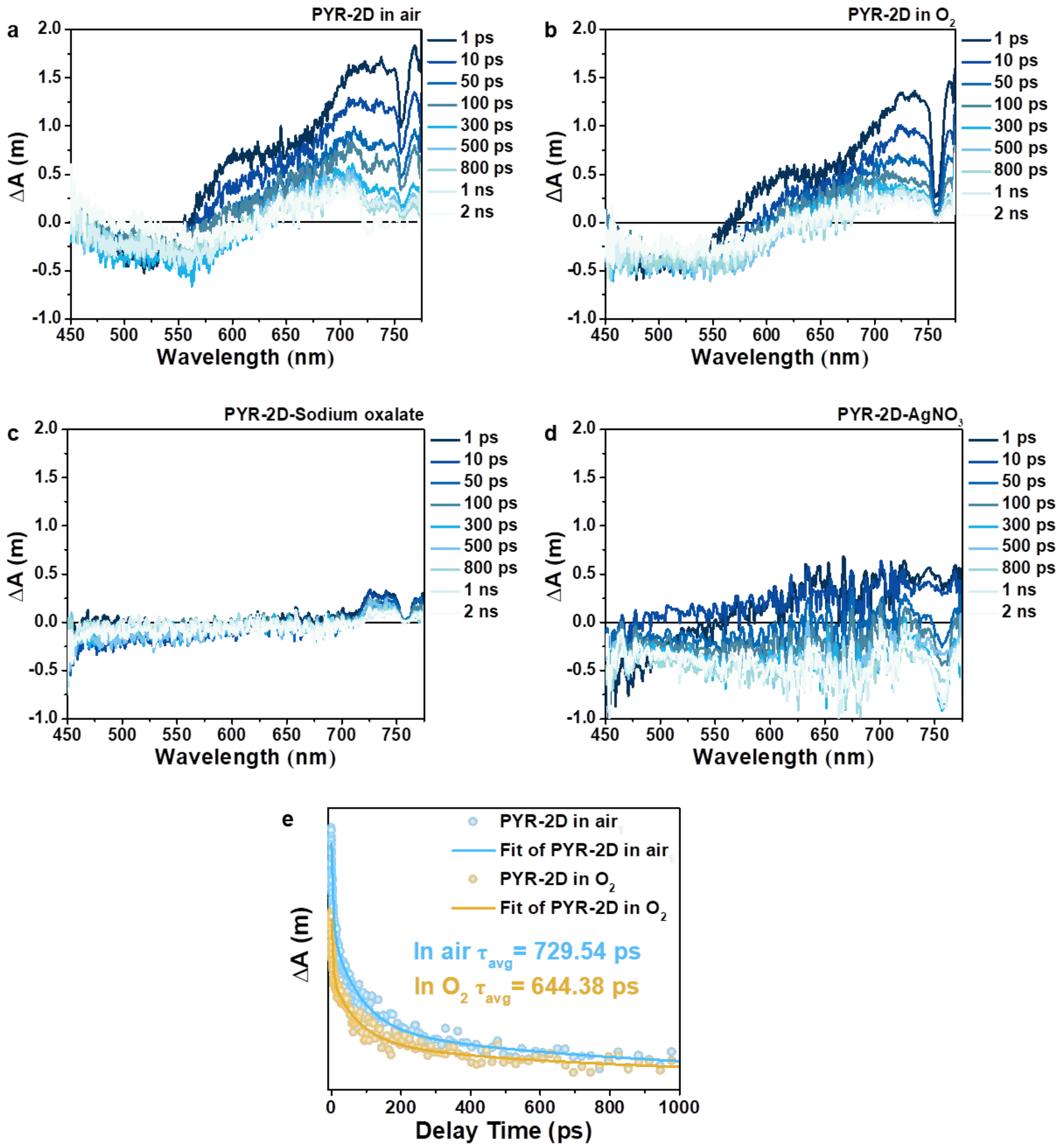


**Supplementary Fig. 51 | Femtosecond time-resolved transient absorption (TA) measurements of PYR-2D in the visible region on ps and ns scales. a–d**, The TA spectra in the 450–780 nm range of (**a**) PYR-2D in air, (**b**) PYR-2D in $O_2$, (**c**) PYR-2D with sodium oxalate (10 mM), (**d**) PYR-2D with $AgNO_3$ (10 mM) on ps and ns scales. Signals were drastically reduced regardless of adding hole sacrificial agents (sodium oxalate) or electron sacrificial agents ($AgNO_3$), which proved that the signal might be attributed to polarons. **e**, Comparison of TA kinetic profiles at 720 nm under air and $O_2$ conditions, respectively. The specific values of decay lifetimes fitted by the tri-exponential function are shown in Fig. 8e.

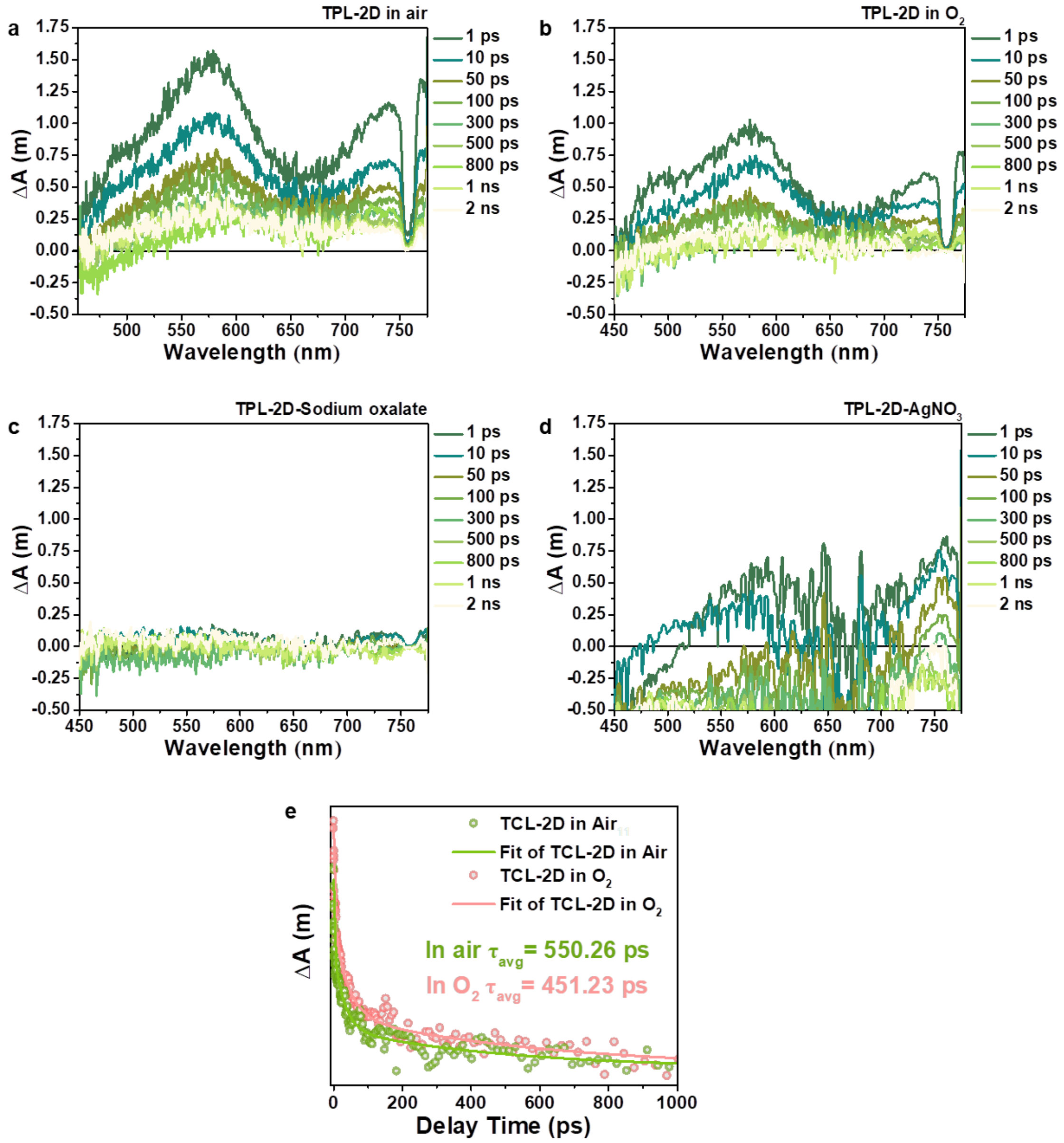


**Supplementary Fig. 52 | Femtosecond time-resolved transient absorption (TA) measurements of TPL-2D in the visible region on ps and ns scales. a–d**, The TA spectra in the 450–780 nm range of (**a**) TPL-2D in air, (**b**) TPL-2D in $O_2$, (**c**) TPL-2D with sodium oxalate (10 mM), (**d**) TPL-2D with $AgNO_3$ (10 mM) on picosecond and nanosecond scales. Signals were drastically reduced regardless of adding hole sacrificial agents (sodium oxalate) or electron sacrificial agents ($AgNO_3$), which proved that the signal might be attributed to polarons. **e**, Comparison of TA kinetic profiles at 580 nm under air and $O_2$ conditions, respectively. The specific values of decay lifetimes fitted by the tri-exponential function are shown in Fig. 8e.

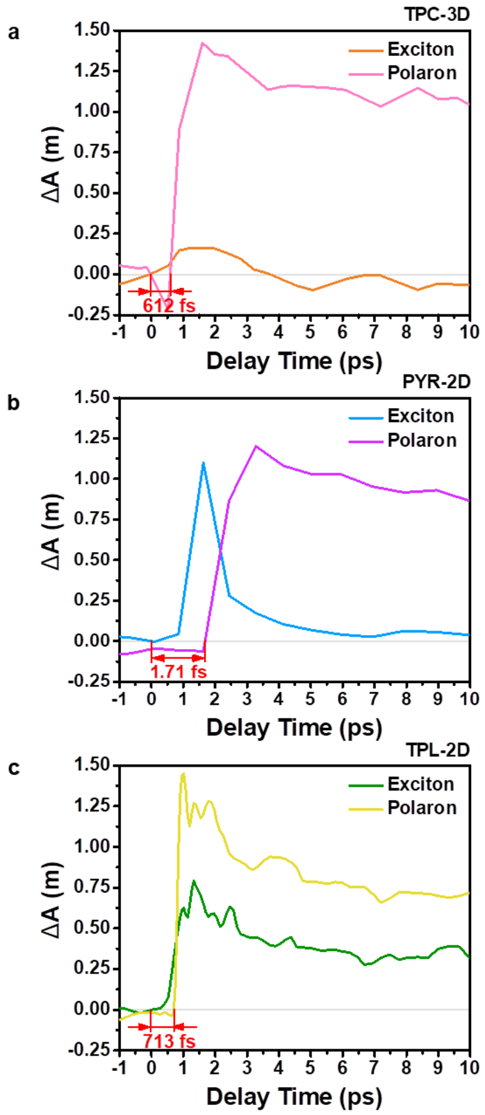


**Supplementary Fig. 53 | Femtosecond time-resolved transient absorption (TA) kinetic profiles in the visible light region under oxygen atmosphere at specific wavelength. a–c,** TA kinetic profiles of signal peaks which attributed to excitons and polarons in TA spectra of (**a**) TPC-3D, (**b**) PYR-2D, (**c**) TPL-2D under $O_2$ atmosphere. The time delay between excitons and polarons was the time of excitons transferring to polarons. By comparing the generation rates of polarons in air (Fig. 8c and Supplementary Figs. 48c, 49c), we verified that the generation rates of polarons did not increase in the presence of oxygen, thereby eliminating the possibility of accelerated polarons separation contributing to the acceleration of polarons generation.

a

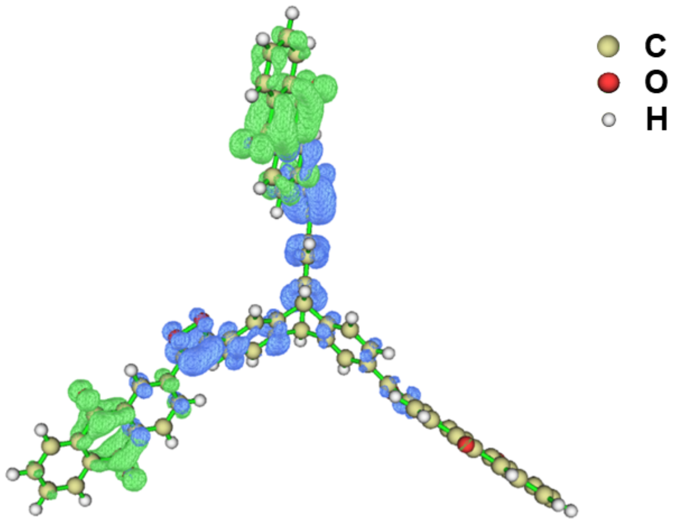


b

| | D (Å) | $S_m$ | $S_r$ | H (Å) | t (Å) | E (eV) | $E_{coul}$ (eV) | HDI | EDI |
|---|---|---|---|---|---|---|---|---|---|
| TPC-3D | 4.53 | 0.23 | 0.48 | 7.57 | 1.84 | 2.72 | 1.96 | 4.44 | 5.66 |
| TPC-3D-$O_2$ | 3.58 | 0.24 | 0.51 | 6.98 | 0.73 | 2.48 | 2.03 | 4.95 | 5.80 |

**Supplementary Fig. 54 | Time-dependent density functional theory (TD-DFT) calculations after oxygen adsorption. a**, Analysis for the distribution of the holes (blue) and electrons (green) for TPC-3D after absorbing oxygen. **b**, various indices of the hole and electron functions for TPC-3D after absorbing oxygen. TD-DFT calculations proved that the excitonic binding energy of TPC-3D was slightly increased after $O_2$ adsorption, excluding the reason explanation that the absorbed $O_2$ could provide an additional built-in electric field to accelerate polaron dissociation.

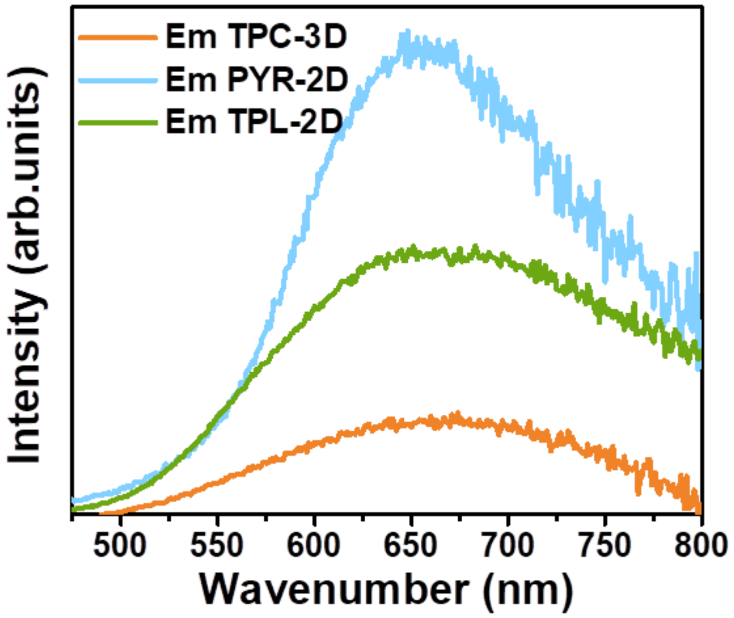


**Supplementary Fig. 55 | Photoluminescence spectra of CPs.** The photoluminescence of TPC-3D appeared weaker than that of PYR-2D and TPL-2D.

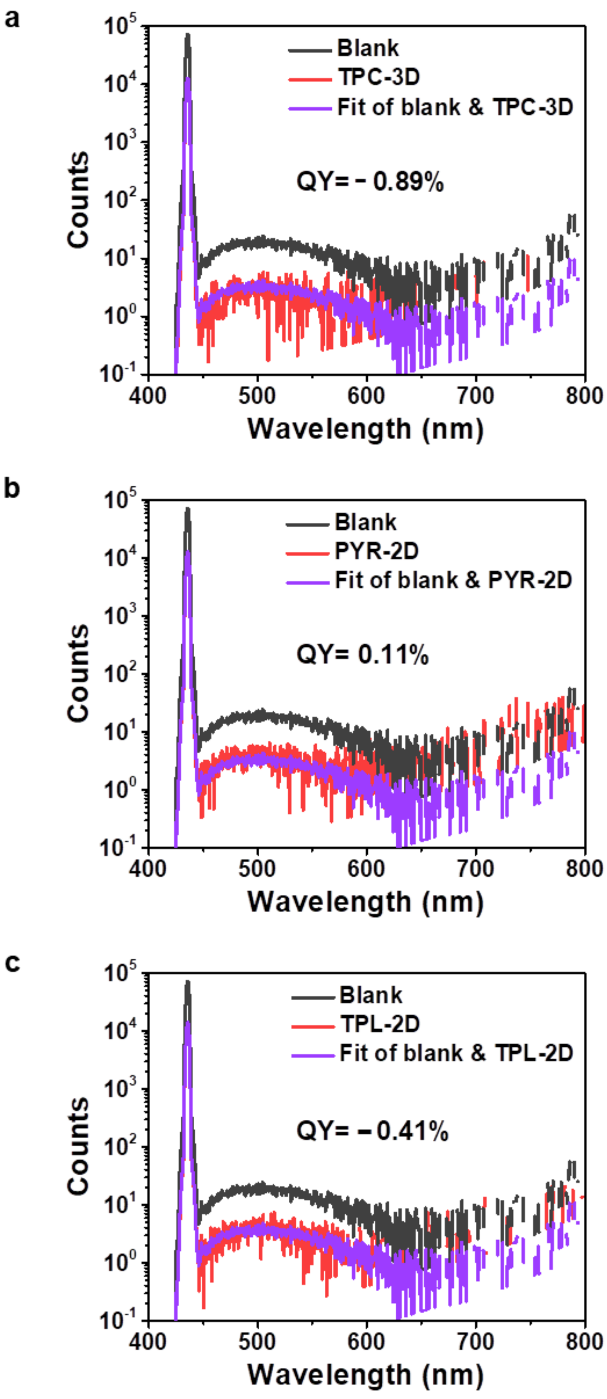


**Supplementary Fig. 56 | Photo-luminescence quenching yields (PLQYs) measurements. a–c**, PLQYs measurements of (**a**) TPC-3D, (**b**) PYR-2D, (**c**) TPL-2D. The PLQYs of PYR-2D was 0.11%, while the PLQYs of TPC-3D and TPL-2D were even below the detectable limit, which indicated that the radiative recombination of excitons was almost completely suppressed in the CPs.

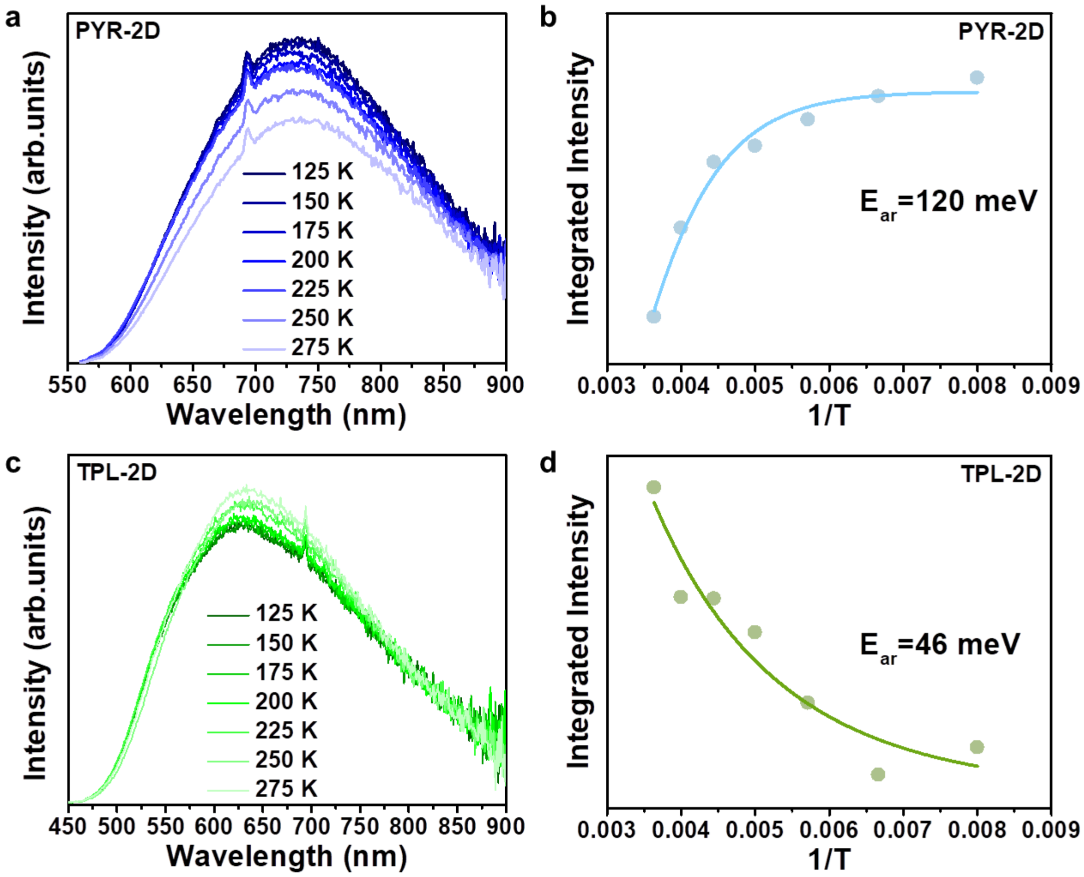


**Supplementary Fig. 57 | Temperature-dependent photoluminescence (TD-PL) measurement to obtain the activation energy of exciton dissociation ($E_a$) or activation energy of charge recombination ($E_{ar}$). a**, **c**, TD-PL spectra of (**a**) PYR-2D and (**c**) TPL-2D. **b**, **d**, The $E_a$ or $E_{ar}$ were calculated based on the evolution of PL intensity as a function of temperature for (**b**) PYR-2D and (**d**) TPL-2D. The $E_{ar}$ for TPC-3D (as shown in Fig. 9a, b) and TPL-2D could be estimated to be 23 meV and 46 meV, respectively, which indicated that the excitons in TPC-3D and TPL-2D could spontaneously dissociated. In contrast, the $E_a$ for PYR-2D was 120 meV.

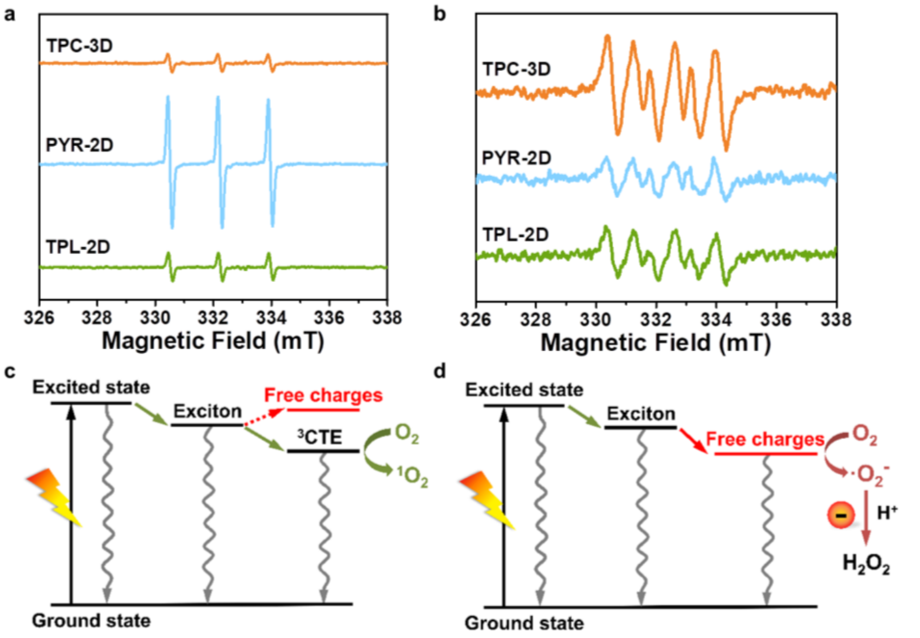


**Supplementary Fig. 58 | Electron paramagnetic resonance (EPR) spectra of different CPs to clarify the reduction medium of $O_2$. a**, EPR spectra of CPs in the presence of TEMP as a radical spin-trapping agent in $O_2$ atmosphere under visible light irradiation to examine the existence of singlet oxygen. **b**, EPR spectra of CPs by using DMPO as a radical spin-trapping agent in $O_2$ atmosphere under visible light irradiation to examine the existence of superoxide radicals during oxygen reduction. A significant amount of singlet oxygen were detected in the PYR-2D, and the signal intensity of superoxide radical was high in TPC-3D with the light irradiation, which indicated that PYR-2D was dominated by the generation of singlet oxygen and TPC-3D was dominated by the generation of superoxide radical during the oxygen reduction. **c**, Steps for PYR-2D to generate spin-triplet charge-transfer exciton ($^3$CTE) by spin flip, which would react with $^1O_2$. **d**, Steps for TPC-3D and TPL-2D to generate $\cdot O_2^-$ through electrons transfer. The much more production of $^1O_2$ in PYR-2D also confirmed that TPC-3D and TPL-2D exhibited higher excitonic dissociation efficiency. This is because when the excitons dissociated into electrons, $O_2$ tended to generate $\cdot O_2^-$ through electron transfer. On the other hand, when the excitons could not dissociate efficiently, they would go through spin-flip and transform to triplet excitons, which would react with $O_2$ to generate $^1O_2$ through energy transfer.

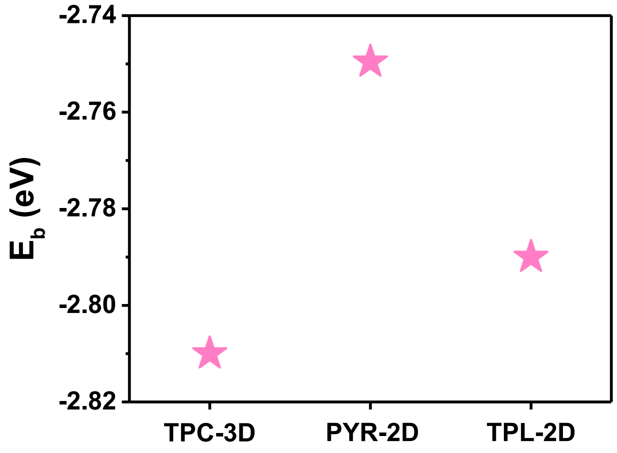


**Supplementary Fig. 59 | Calculated excitonic binding energy ($E_b$) for the TPC-3D, PYR-2D, TPL-2D molecules.** For simplicity, individual D-A molecules were constructed for calculation. The trend of simulated excitonic binding energies ($E_b$) of the CPs was consistent with the experimentally observed exciton dissociation efficiencies. Notably, the simulated $E_b$ were two orders of magnitude higher than the experimental values of $E_a$ and $E_{ar}$, which indicated that polymerization effectively reduced the $E_b$ by extending the conjugation structures.

a

b

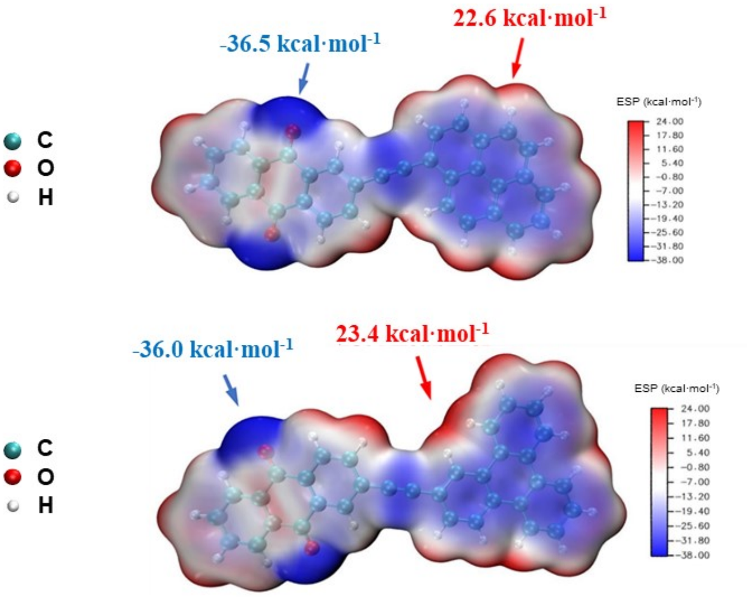


c

| | TPC-3D | RYR-2D | TPL-2D |
|---|---|---|---|
| Internal charge separation (Pi)/kcal·mol$^{-1}$ | 10.2 | 10.1 | 9.9 |
| Molecular polarity index (MPI)/kcal·mol$^{-1}$ | 10.2 | 10.1 | 10.0 |

**Supplementary Fig. 60 | The electrostatic potential (ESP). a–b**, The ESP of (**a**) PYR-2D, (**b**) TPL-2D. **c**, The surface analysis of CPs. The larger the molecular polarity index (MPI), the larger the overall polarity of the molecule. Since the uneven charge distribution of the system reflects molecular polarity, a more uneven distribution results in more positive or negative areas of electrostatic potential on the molecule's surface, leading to an increased MPI. It showed that the promoted charge separation in excited TPC-3D was attributed to the stronger delocalization.

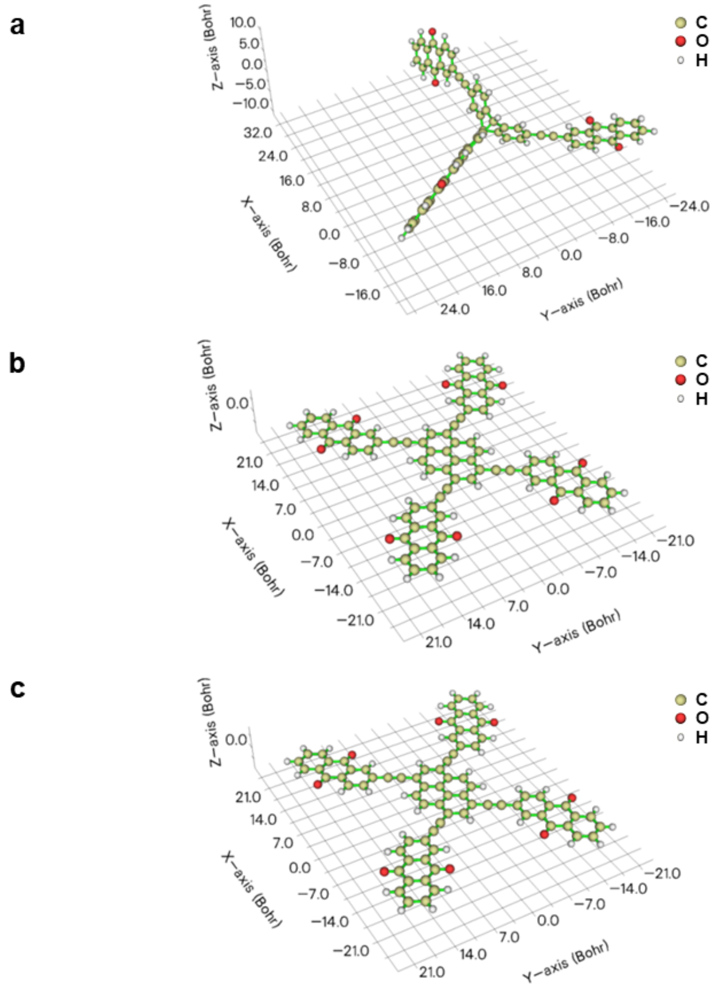


**d**

| Dipole moment | TPC-3D | PYR-2D | TPL-2D |
|---|---|---|---|
| X (Debye) | 0.4303 | 0.0000 | 0.4189 |
| Y (Debye) | 0.2210 | 0.0001 | 0.0000 |
| Z (Debye) | -0.7511 | -0.0001 | 0.0000 |
| Tot (Debye) | 0.8934 | 0.0001 | 0.4189 |

**Supplementary Fig. 61 | The dipole moment of CPs. a–c**, Computational model of the dipole moment of (**a**) TPC-3D, (**b**) PYR-2D, (**c**) TPL-2D. **d**, Specific values of the dipole moment in each direction of CPs. The dipole moment of TPC-3D was much higher than that of PYR-2D and TPL-2D, owing to the addition of dipole moment from the other two dimensions caused by the asymmetry.

## Supplementary Tables

**Supplementary Table 1 | The comparison of the hydrogen peroxide yield between TPC-3D and other reported polymer semiconductor photocatalysts.**

| Catalyst | Water body | Atmosphere | Light intensity | $H_2O_2$ yield /μmol·g$^{-1}$·h$^{-1}$ | Ref. |
|---|---|---|---|---|---|
| Ag@U-g-$C_3N_4$-NS-1.0 | Acidic aqueous solution | $O_2$ | 100 (AM 1.5 G) | ~70 | 7 |
| Ni4%/O0.2tCN | Water: ethanol=9:1 | Air | 100 (λ > 400 nm) | 2464 | 8 |
| $NiIn_2S_4$-$C_3N_4$ | Water: ethanol= 9:1 | $O_2$ | 100 (λ > 400 nm) | 2700 | 9 |
| g-$C_3N_4$/$PDI_{51}$ | pure water | $O_2$ | 26.9 (λ > 420 nm) | 21 | 10 |
| $Co_1$/AQ/$C_3N_4$ | pure water | $O_2$ | 100 (AM 1.5 G) | 124 | 11 |
| PEI/$C_3N_4$ | pure water | $O_2$ | 100 (AM 1.5G) | 208 | 12 |
| g-$C_3N_4$/PDI/r$GO_{0.05}$ | pure water | $O_2$ | 43.3 (λ = 420-500 nm) | 21 | 13 |
| CTF-BDDBN | pure water | $O_2$ | 44.5 (λ > 420 nm) | 97 | 14 |
| RF523 | pure water | $O_2$ | 100 (AM 1.5G) | 200 | 15 |
| RF/P3HT-1.0 | pure water | $O_2$ | 100 (AM 1.5G) | 615 | 16 |
| Sb-SAPC | pure water | $O_2$ | 100 (AM 1.5G) | 1179 | 17 |
| PQTEE-COP | pure water | $O_2$ | λ > 400 nm | 3009 | 18 |
| Bpt-CTF | pure water | $O_2$ | λ > 400 nm | 3268 | 19 |
| AQTEE-COP | pure water | $O_2$ | λ > 400 nm | 3204 | 20 |
| ZnPPc-NBCN | pure water | Air | 100 (λ > 400 nm) | 114 | 21 |
| TPE-AQ | pure water | Air | 100 (λ > 400 nm) | 909 | 22 |
| TPT-3 | pure water | Air | 100 (λ > 400 nm) | 1351 | 23 |

| | | | | | |
|---|---|---|---|---|---|
| KDBT | pure water | Air | 149 | ~145 | 24 |
| TPT-alkynyl-AQ | pure water | Air | 100 ($\lambda > 400$ nm) | 2368 | 25 |
| | pure water | Air | ambient conditions | 2676 | |
| | river water | Air | ambient conditions | 2464 | |
| | seawater | Air | ambient conditions | 2746 | |
| LBOB | seawater | Air | $\lambda = 427$ nm | 497 | 26 |
| TPC-3D | pure water | Air | 100 ($\lambda > 400$ nm) | 5940 | This work |
| | Lake water | | | 9991 | |
| | River water | | | 9615 | |
| | Seawater | | | 9257 | |

**Supplementary Table 2 | Comparison of reported apparent quantum yield (AQY) values for photocatalytic $H_2O_2$ production at specific wavelengths.**

| Photocatalysts | AQY | | Ref. |
|---|---|---|---|
| | ~420 nm | ~600 nm | |
| g-$C_3N_4$/$PDI_{51}$ | 2.5 % | / | 10 |
| g-$C_3N_4$/PDI/$rGO_{0.05}$ | 6.1 % | / | 13 |
| g-$C_3N_4$/$BDI_{50}$ | 4.6 % | / | 27 |
| g-$C_3N_4$/$MTI_{49}$ | 6.1 % | / | 28 |
| KPD-CN-7.5 | 8% | ~1.5 % | 29 |
| g-$C_3N_4$/PDI-BN0.2-$rGO_{0.05}$ | 7.3% | / | 30 |
| $R_{370}$-CN | 4.3% | / | 31 |
| RF523 resin | 8.0% | 2.0% | 15 |
| RF-$(COOH)_2$-523 | 8.9% | 4.0% | 32 |
| 5Cv@g-$C_3N_4$ | 9.6% | / | 33 |
| Sb-SAPC15 | 17.6% | ~0.1% | 17 |
| RF/P3HT-1.0 | 10.5% | ~7% | 16 |
| PM-CDs-30 | 1.0% (Seawater) | 0.54% (Seawater) | 34 |
| CTF-DPDA | 16.0% | ~5.6% | 35 |
| Bpt-CTF | 8.6% | 0.4% | 19 |
| TPT-alkynyl-AQ | 18% | / | 25 |
| $Ni4_{\%}/O_{0.2}tCN$ | 14.9% ($H_2O$:EtOH =9:1) | 0.7% ($H_2O$:EtOH =9:1) | 8 |
| CoPc-BTM-COF | ~8.9% ($H_2O$:EtOH =9:1) | 7.2% ($H_2O$:EtOH =9:1) | 36 |
| $CoO_x$/Mo:$BiVO_4$/(Ag/Pd) | 13.1% | ~0% at 590nm | 37 |
| HEP-TAPT-COF | 15.35% | 1.92% | 38 |
| RF-DHAQ-2 | 11.6% | / | 39 |
| TTF-BT-COF | 11.2% | / | 40 |
| SA-TCPP | 14.90% | / | 41 |
| CNIO-GaSA | 7.1% | / | 42 |
| TPC-3D | 30.1% (Pure water)<br>44.8% (Lake water) | 10.6% (Pure water)<br>11.9% (Lake water) | This work |

**Supplementary Table 3 | Solar-to-chemical conversion (SCC) efficiencies for various photocatalytic reactions in recent literature.**

| Photocatalysts | Conditions | | Concentration | SCC | Ref. |
|---|---|---|---|---|---|
| | Atmosphere | Water body | | | |
| g-$C_3N_4$/PDI/rGO$_{0.05}$ | $O_2$ | Pure water | 5 g/L | 0.2% | 13 |
| g-$C_3N_4$/BDI$_{50}$ | $O_2$ | Pure water | 5 g/L | 0.13% | 27 |
| g-$C_3N_4$/MTI$_{49}$ | $O_2$ | Pure water | 5 g/L | 0.18% | 28 |
| g-$C_3N_4$/PDI-BN0.2-rGO$_{0.05}$ | $O_2$ | Pure water | 5 g/L | 0.27 % | 30 |
| $R_{370}$-CN | $O_2$ | Pure water | 4 g/L | 0.26% | 31 |
| RF523 resin | $O_2$ | Pure water | 5 g/L | 0.2% | 15 |
| RF-$(COOH)_2$-523 | $O_2$ | Pure water (60 °C) | 5 g/L | 0.7% | 32 |
| Sb-SAPC15 | $O_2$ | Pure water | 5 g/L | 0.61% | 17 |
| RF/P3HT-1.0 | $O_2$ | Pure water | 3 g/L | 1.0% | 16 |
| CTF-DPDA | $O_2$ | Pure water | 5 g/L | 0.78% | 35 |
| TPE-AQ | Air | Pure water | 1.25 g/L | 0.26% | 22 |
| TPT-alkynyl-AQ | Air | Pure water | 1 g/L | 0.35% | 25 |
| $CoO_x$/Mo:$BiVO_4$/(Ag/Pd) | $O_2$ | Water with $H_3BO_3$, $ScCl_3$ | 1 g/L | 0.73% | 37 |
| HEP-TAPT-COF | $O_2$ | Pure water | 5 g/L | 0.65% | 38 |
| RF-DHAQ-2 | $O_2$ | Pure water (50 °C) | 2.67 g/L | 1.2% | 39 |
| TTF-BT-COF | $O_2$ | Pure water | 0.5 g/L | 0.49% | 40 |
| SA-TCPP | $O_2$ | Pure water (55 °C) | 3 g/L | 1.2% | 41 |

| | | | | | |
|---|---|---|---|---|---|
| CNIO-GaSA | $O_2$ | Pure water | 1 g/L | 0.40% | 42 |
| TPC-3D | Air | Pure water | 0.4 g/L | 2.4% | This work |
| | | Lake water | | 3.6% | |

**Supplementary Table 4 | Specific parameters of water samples.** The photosynthetic rate of $H_2O_2$ by TPC-3D in different water bodies: in lake water > in river water > in seawater > in pure water. It was observed that the increasing rate was positively correlated with the content of dissolved organic matters (DOMs) in real water.

| | | Pure water | Lake water | River water | Seawater |
|---|---|---|---|---|---|
| Dissolved oxygen (mg/L) | | 7.09 | 8.11 | 6.51 | 8.49 |
| pH | | 6.13 | 7.53 | 6.52 | 8.39 |
| Total Carbon (mg/L) | | / | 22.21 | 15.42 | 26.61 |
| Inorganic carbon (mg/L) | | / | 14.60 | 8.49 | 24.97 |
| Non-purgeable organic carbon (mg/L) | | / | 7.50 | 5.95 | 2.24 |
| Total Nitrogen (mg/L) | | / | 2.13 | 1.00 | 0.53 |
| Ion concentration (mM) | $Cl^-$ | / | 3.9430 | 0.2152 | 548.1175 |
| | $SO_4^{2-}$ | / | 0.5680 | 0.0448 | 22.6973 |
| | $NO_3^-$ | / | 0.0008 | 0.0016 | 0.1213 |
| | $Br^-$ | / | 0.0045 | 0.0003 | 0.7719 |

**Supplementary Table 5 | Various indices of the hole and electron functions calculated by time-dependent density functional theory (TD-DFT) calculations.** *D*, the distance between the center of mass of holes and electrons. $S_m$ and $S_r$, degree of overlap of holes and electrons, the larger the value, the higher the overlap of holes and electrons. *H*, overall average distribution breadth of holes and electrons. *t*, separation degree of holes and electrons. *E*, excitation energy. $E_{coul}$, the Coulomb attractive energy. *HDI*, hole delocalization index, the smaller the value, the higher the hole delocalization degree. *EDI*, electron delocalization index, the smaller the value, the higher the electron delocalization degree.

| | ***D* (Å)** | $S_m$ | $S_r$ | ***H* (Å)** | ***t* (Å)** | ***E* (eV)** | $E_{coul}$ **(eV)** | ***HDI*** | ***EDI*** |
|---|---|---|---|---|---|---|---|---|---|
| TPC-3D | 4.53 | 0.23 | 0.48 | 7.57 | 1.84 | 2.72 | 1.96 | 4.44 | 5.66 |
| PYR-2D | 0.00 | 0.46 | 0.74 | 6.71 | -2.79 | 2.07 | 2.44 | 4.47 | 3.31 |
| TPL-2D | 0.52 | 0.36 | 0.65 | 8.38 | -5.29 | 2.63 | 1.98 | 3.27 | 2.93 |

**Supplementary Table 6 | Atomic coordinates of the TPC-3D optimized computational model.**

| Atom | x | y | z |
|---|---|---|---|
| C | -3.463596 | -0.548444 | 0.342213 |
| C | -2.089502 | -0.453373 | 0.446805 |
| C | -1.27768 | -0.528177 | -0.695641 |
| C | -4.047063 | -0.724473 | -0.929654 |
| C | -3.227909 | -0.803193 | -2.067429 |
| C | -1.843809 | -0.704089 | -1.949954 |
| C | -0.150339 | -2.325945 | 2.806209 |
| C | 0.815557 | -3.323125 | 2.695411 |
| C | 1.631781 | -3.40099 | 1.555629 |
| C | -0.302226 | -1.40708 | 1.77757 |
| C | 0.508556 | -1.479903 | 0.634416 |
| C | 1.470384 | -2.463759 | 0.514042 |
| C | -0.456751 | 2.130976 | 2.303952 |
| C | -0.465635 | 0.994036 | 1.508664 |
| C | 0.343542 | 0.92208 | 0.364426 |
| C | 0.359895 | 3.204061 | 1.955018 |
| C | 1.170335 | 3.139837 | 0.810232 |
| C | 1.158079 | 1.979143 | 0.009137 |
| C | 0.203228 | -0.393855 | -0.38491 |
| C | -1.297042 | -0.258943 | 1.731147 |
| C | -5.458917 | -0.816287 | -1.058037 |
| C | 2.620414 | -4.41554 | 1.4487 |
| C | 3.472237 | -5.277805 | 1.349653 |
| C | 5.150631 | 8.423156 | -1.013179 |
| C | 5.178663 | 7.230379 | -1.739448 |
| C | 4.381192 | 6.16159 | -1.368413 |
| C | 3.529703 | 6.263411 | -0.250019 |
| C | 3.504017 | 7.460721 | 0.479749 |
| C | 4.305055 | 8.532577 | 0.103681 |
| C | 4.243738 | 9.785218 | 0.903474 |
| C | 6.014891 | 9.549733 | -1.439116 |
| C | 5.950315 | 10.802228 | -0.643981 |
| C | 5.105023 | 10.914335 | 0.473276 |
| O | 3.509282 | 9.878001 | 1.877422 |
| O | 6.753206 | 9.450519 | -2.411047 |
| C | -10.866472 | -1.127828 | -1.570525 |
| C | -10.039766 | -1.311014 | -2.681379 |
| C | -8.66358 | -1.234246 | -2.554636 |
| C | -8.078186 | -0.970695 | -1.300095 |
| C | -8.909186 | -0.787224 | -0.185318 |

| | | | |
|---|---|---|---|
| C | -10.290909 | -0.863835 | -0.316133 |
| C | -11.13526 | -0.659081 | 0.891389 |
| C | -12.336371 | -1.21568 | -1.740838 |
| C | -13.179737 | -1.01013 | -0.535967 |
| C | -12.606713 | -0.744024 | 0.719522 |
| C | -13.429554 | -0.555255 | 1.830649 |
| C | -14.567342 | -1.084037 | -0.661064 |
| C | -14.810835 | -0.629732 | 1.69729 |
| C | -15.380064 | -0.894459 | 0.450342 |
| O | -10.632397 | -0.429 | 1.982635 |
| O | -12.835552 | -1.448548 | -2.834556 |
| C | 1.997711 | 4.237453 | 0.451187 |
| C | 2.7068 | 5.171897 | 0.130305 |
| C | 6.458585 | -8.240254 | 0.952995 |
| C | 5.605752 | -8.24564 | 2.059219 |
| C | 4.624498 | -7.278682 | 2.196958 |
| C | 4.473091 | -6.275057 | 1.219519 |
| C | 5.329616 | -6.271051 | 0.108972 |
| C | 6.314409 | -7.242214 | -0.025611 |
| C | 7.201935 | -7.197915 | -1.218207 |
| C | 7.49862 | -9.290088 | 0.835084 |
| C | 8.386751 | -9.244968 | -0.35435 |
| C | 8.244956 | -8.246704 | -1.33359 |
| C | 9.097653 | -8.233246 | -2.438013 |
| C | 9.37979 | -10.214901 | -0.494834 |
| C | 10.08444 | -9.20273 | -2.570112 |
| C | 10.225502 | -10.194383 | -1.597618 |
| O | 7.074461 | -6.328187 | -2.069085 |
| O | 7.620461 | -10.16096 | 1.687323 |
| C | 6.746491 | 11.884429 | -1.020183 |
| C | 5.068367 | 12.106702 | 1.197204 |
| C | 6.704052 | 13.068778 | -0.294083 |
| C | 5.864136 | 13.180103 | 0.815482 |
| C | -6.667974 | -0.888967 | -1.166715 |
| H | -4.095289 | -0.487954 | 1.223115 |
| H | -3.685374 | -0.939444 | -3.041934 |
| H | -1.214492 | -0.762471 | -2.833176 |
| H | -0.77796 | -2.269245 | 3.690712 |
| H | 0.945876 | -4.048294 | 3.492056 |
| H | 2.100259 | -2.522411 | -0.368329 |
| H | -1.082462 | 2.186505 | 3.189875 |
| H | 0.374852 | 4.100514 | 2.566284 |
| H | 1.783758 | 1.927031 | -0.876572 |
| H | 0.834065 | -0.450659 | -1.273536 |

| | | | |
|---|---|---|---|
| H | -1.928477 | -0.202386 | 2.619487 |
| H | 5.837286 | 7.162181 | -2.598969 |
| H | 4.404341 | 5.236964 | -1.935568 |
| H | 2.858345 | 7.56678 | 1.344773 |
| H | -10.500447 | -1.513533 | -3.64258 |
| H | -8.02414 | -1.376364 | -3.419521 |
| H | -8.485189 | -0.581697 | 0.791647 |
| H | -12.966143 | -0.351267 | 2.79037 |
| H | -14.989168 | -1.291192 | -1.639105 |
| H | -15.447151 | -0.481938 | 2.564261 |
| H | -16.459217 | -0.952583 | 0.347816 |
| H | 5.730164 | -9.022928 | 2.805963 |
| H | 3.964458 | -7.285878 | 3.058028 |
| H | 5.235253 | -5.510134 | -0.658195 |
| H | 8.970388 | -7.453629 | -3.181985 |
| H | 9.472579 | -10.976296 | 0.272696 |
| H | 10.745882 | -9.188306 | -3.430737 |
| H | 10.99687 | -10.951069 | -1.702025 |
| H | 7.39175 | 11.77647 | -1.88585 |
| H | 4.408662 | 12.171606 | 2.056288 |
| H | 7.325156 | 13.908149 | -0.590997 |
| H | 5.832034 | 14.105928 | 1.381348 |

**Supplementary Table 7 | Atomic coordinates of the PYR-2D optimized computational model.**

| Atom | x | y | z |
|---|---|---|---|
| C | 3.590934 | 2.43283 | -0.008292 |
| C | -3.59087 | 2.432841 | 0.008053 |
| C | -3.590873 | -2.432803 | -0.008444 |
| C | 3.590922 | -2.432814 | 0.008178 |
| C | 4.223183 | 3.472835 | -0.015984 |
| C | -4.223112 | 3.472851 | 0.015779 |
| C | 4.223147 | -3.472833 | 0.015888 |
| C | -4.223126 | -3.472806 | -0.016134 |
| C | 6.406549 | 7.100402 | -0.055773 |
| C | 5.007157 | 7.116529 | 0.010232 |
| C | 4.286431 | 5.931613 | 0.023664 |
| C | 4.957453 | 4.688362 | -0.027643 |
| C | 6.362391 | 4.672723 | -0.092911 |
| C | 7.08403 | 5.864928 | -0.107896 |
| C | 8.573701 | 5.803291 | -0.182187 |
| C | 7.14882 | 8.389329 | -0.070007 |
| C | 8.635182 | 8.328266 | -0.146415 |
| C | 9.315247 | 7.093787 | -0.199918 |
| O | 9.162045 | 4.725374 | -0.227643 |
| O | 6.555117 | 9.46565 | -0.02104 |
| C | 9.365307 | 9.521859 | -0.164959 |
| C | 10.712833 | 7.074627 | -0.271352 |
| C | 10.756273 | 9.493121 | -0.236336 |
| C | 11.431005 | 8.268039 | -0.289524 |
| C | -7.083938 | 5.864954 | 0.107917 |
| C | -6.362306 | 4.672746 | 0.092872 |
| C | -4.957372 | 4.688378 | 0.027492 |
| C | -4.28635 | 5.931628 | -0.023864 |
| C | -5.007069 | 7.116547 | -0.010367 |
| C | -6.406457 | 7.100426 | 0.055752 |
| C | -7.148722 | 8.389354 | 0.070061 |
| C | -8.573604 | 5.803321 | 0.182319 |
| C | -9.315145 | 7.093819 | 0.200105 |
| C | -8.63508 | 8.328296 | 0.146562 |
| O | -6.555022 | 9.465675 | 0.021033 |
| O | -9.161943 | 4.725406 | 0.22786 |
| C | -10.712726 | 7.074663 | 0.271633 |
| C | -9.365199 | 9.521891 | 0.165157 |
| C | -11.430893 | 8.268078 | 0.289854 |
| C | -10.75616 | 9.493158 | 0.236626 |

| | | | |
|---|---|---|---|
| C | -6.406528 | -7.100351 | -0.055833 |
| C | -5.007131 | -7.116489 | 0.010109 |
| C | -4.286397 | -5.93158 | 0.023507 |
| C | -4.957413 | -4.688322 | -0.027769 |
| C | -6.362352 | -4.672673 | -0.092976 |
| C | -7.084002 | -5.864874 | -0.107928 |
| C | -8.573676 | -5.803225 | -0.182147 |
| C | -7.148809 | -8.389273 | -0.070026 |
| C | -8.635174 | -8.328199 | -0.146368 |
| C | -9.315232 | -7.093715 | -0.199843 |
| O | -9.162013 | -4.725304 | -0.22757 |
| O | -6.555112 | -9.465598 | -0.021067 |
| C | -9.365308 | -9.521787 | -0.164878 |
| C | -10.712821 | -7.074546 | -0.271214 |
| C | -10.756277 | -9.49304 | -0.236194 |
| C | -11.431002 | -8.267952 | -0.289353 |
| C | 8.634994 | -8.328397 | 0.146499 |
| C | 9.365084 | -9.522012 | 0.165029 |
| C | 10.756043 | -9.493318 | 0.236566 |
| C | 11.430802 | -8.268259 | 0.289929 |
| C | 10.712665 | -7.074826 | 0.271775 |
| C | 9.315086 | -7.093941 | 0.200181 |
| C | 8.573577 | -5.803424 | 0.182476 |
| C | 7.148639 | -8.389413 | 0.069912 |
| C | 6.406405 | -7.100465 | 0.055685 |
| C | 7.083914 | -5.865015 | 0.107985 |
| O | 9.161944 | -4.725527 | 0.228089 |
| O | 6.554912 | -9.465715 | 0.020811 |
| C | 5.00702 | -7.116547 | -0.010498 |
| C | 6.362311 | -4.672788 | 0.092995 |
| C | 4.28633 | -5.93161 | -0.023934 |
| C | 4.957381 | -4.688382 | 0.027548 |
| C | 1.423623 | 1.239362 | -0.000867 |
| C | 0.716177 | 0.000015 | -0.000122 |
| C | 1.423617 | -1.239334 | 0.000654 |
| C | 2.850273 | -1.222463 | 0.002412 |
| C | 3.533924 | 0.000008 | -0.00006 |
| C | 2.850277 | 1.222484 | -0.002561 |
| C | -0.716123 | 0.000017 | -0.000152 |
| C | -1.423567 | -1.23933 | -0.000931 |
| C | -0.682647 | -2.459596 | -0.000782 |
| C | 0.682693 | -2.459598 | 0.000504 |
| C | 0.682702 | 2.45963 | -0.000744 |
| C | -0.682637 | 2.459632 | 0.000488 |

| | | | |
|---|---|---|---|
| C | -1.423564 | 1.239367 | 0.000601 |
| C | -2.850218 | 1.222497 | 0.002302 |
| C | -3.533869 | 0.000021 | -0.000203 |
| C | -2.850223 | -1.222452 | -0.002685 |
| H | 4.50101 | 8.074429 | 0.04995 |
| H | 3.203106 | 5.949382 | 0.073552 |
| H | 6.900477 | 3.732941 | -0.133944 |
| H | 8.825013 | 10.460965 | -0.122796 |
| H | 11.217193 | 6.115638 | -0.311959 |
| H | 11.316467 | 10.422555 | -0.250568 |
| H | 12.51464 | 8.246669 | -0.344982 |
| H | -6.900393 | 3.732967 | 0.133943 |
| H | -3.203028 | 5.949393 | -0.073839 |
| H | -4.500922 | 8.074444 | -0.050119 |
| H | -11.217087 | 6.115676 | 0.312275 |
| H | -8.824904 | 10.460995 | 0.122963 |
| H | -12.514525 | 8.246711 | 0.345382 |
| H | -11.316351 | 10.422593 | 0.250896 |
| H | -4.500992 | -8.074393 | 0.049806 |
| H | -3.203069 | -5.949355 | 0.073347 |
| H | -6.900435 | -3.732888 | -0.133981 |
| H | -8.825019 | -10.460897 | -0.122735 |
| H | -11.217177 | -6.115553 | -0.311798 |
| H | -11.316478 | -10.42247 | -0.250402 |
| H | -12.51464 | -8.246574 | -0.344762 |
| H | 8.824769 | -10.461099 | 0.122728 |
| H | 11.316209 | -10.422769 | 0.250784 |
| H | 12.514432 | -8.246923 | 0.345509 |
| H | 11.217047 | -6.115854 | 0.312522 |
| H | 4.500852 | -8.07443 | -0.050351 |
| H | 6.900419 | -3.733025 | 0.134167 |
| H | 3.20301 | -5.949344 | -0.073962 |
| H | 4.617842 | 0.000007 | -0.000037 |
| H | -1.227321 | -3.397414 | -0.001311 |
| H | 1.227364 | -3.397418 | 0.001035 |
| H | 1.227377 | 3.397447 | -0.001249 |
| H | -1.227309 | 3.397451 | 0.001001 |
| H | -4.617787 | 0.000025 | -0.000222 |

**Supplementary Table 8 | Atomic coordinates of the TPL-2D optimized computational model.**

| Atom | x | y | z |
|---|---|---|---|
| C | 0.083756 | 1.443303 | 0.037383 |
| C | 1.351025 | 0.711161 | 0.029981 |
| C | 1.351006 | -0.711202 | -0.029977 |
| C | 0.083717 | -1.443311 | -0.037379 |
| C | -1.148567 | -0.731685 | -0.004953 |
| C | -1.148548 | 0.73171 | 0.004959 |
| C | -2.351676 | 1.46378 | -0.026898 |
| C | -2.379499 | 2.853997 | -0.010998 |
| C | -1.139648 | 3.569819 | 0.051448 |
| C | 0.050822 | 2.851522 | 0.066095 |
| C | 2.586827 | 1.38608 | 0.073552 |
| C | 3.804329 | 0.714946 | 0.045605 |
| C | 3.80431 | -0.715053 | -0.045602 |
| C | 2.58679 | -1.386155 | -0.073549 |
| C | 0.050746 | -2.851529 | -0.066091 |
| C | -1.139743 | -3.569794 | -0.051444 |
| C | -2.379575 | -2.853939 | 0.011004 |
| C | -2.351715 | -1.463723 | 0.026905 |
| C | -3.600649 | 3.577335 | -0.07738 |
| C | -1.164645 | 4.98902 | 0.106175 |
| C | -3.600745 | -3.577244 | 0.077386 |
| C | -1.164779 | -4.988994 | -0.106172 |
| C | 5.04375 | 1.405295 | 0.11876 |
| C | 5.043713 | -1.405434 | -0.118759 |
| C | -4.585851 | 4.287305 | -0.143725 |
| C | -1.323661 | 6.193652 | 0.155492 |
| C | -4.585966 | -4.287188 | 0.143731 |
| C | -1.323828 | -6.193622 | -0.15549 |
| C | 6.158881 | -1.88615 | -0.183353 |
| C | 6.15893 | 1.885982 | 0.183354 |
| C | -7.71382 | 7.139479 | -0.235499 |
| C | -7.941611 | 5.87095 | 0.312191 |
| C | -6.934519 | 4.91467 | 0.328723 |
| C | -5.665501 | 5.211634 | -0.212895 |
| C | -5.448288 | 6.474178 | -0.793834 |
| C | -6.456051 | 7.435234 | -0.798286 |
| C | -6.160177 | 8.782529 | -1.363182 |
| C | -8.796077 | 8.160222 | -0.190478 |
| C | -8.492431 | 9.511951 | -0.738177 |
| C | -7.236647 | 9.805448 | -1.308156 |

| | | | |
|---|---|---|---|
| O | -5.051531 | 9.045951 | -1.826231 |
| O | -9.897343 | 7.898277 | 0.290734 |
| C | -9.471607 | 10.509569 | -0.674987 |
| C | -6.980862 | 11.08731 | -1.806742 |
| C | -9.207874 | 11.783872 | -1.172042 |
| C | -7.961388 | 12.07287 | -1.739106 |
| C | -6.456253 | -7.435065 | 0.79829 |
| C | -5.448463 | -6.474037 | 0.793838 |
| C | -5.665642 | -5.211487 | 0.212901 |
| C | -6.934651 | -4.914488 | -0.328716 |
| C | -7.94177 | -5.870739 | -0.312184 |
| C | -7.714014 | -7.139275 | 0.235504 |
| C | -8.796299 | -8.159988 | 0.190483 |
| C | -6.160415 | -8.78237 | 1.363184 |
| C | -7.236914 | -9.805258 | 1.308158 |
| C | -8.492691 | -9.511726 | 0.738181 |
| O | -9.897558 | -7.898012 | -0.290728 |
| O | -5.051777 | -9.045823 | 1.826233 |
| C | -6.981164 | -11.087128 | 1.806743 |
| C | -9.471894 | -10.509317 | 0.674991 |
| C | -7.961717 | -12.072661 | 1.739107 |
| C | -9.208196 | -11.783627 | 1.172045 |
| C | -3.325632 | 9.257988 | 0.781311 |
| C | -2.889727 | 7.936487 | 0.807671 |
| C | -1.670698 | 7.572536 | 0.207399 |
| C | -0.866961 | 8.574512 | -0.376319 |
| C | -1.299289 | 9.894794 | -0.39025 |
| C | -2.533857 | 10.248925 | 0.167907 |
| C | -3.004924 | 11.657082 | 0.07042 |
| C | -4.657465 | 9.583222 | 1.365583 |
| C | -5.145531 | 10.980691 | 1.231674 |
| C | -4.359447 | 11.97015 | 0.606731 |
| O | -2.30719 | 12.526568 | -0.449293 |
| O | -5.340731 | 8.7162 | 1.907166 |
| C | -6.41891 | 11.304572 | 1.712988 |
| C | -4.862069 | 13.269632 | 0.475832 |
| C | -6.911437 | 12.599005 | 1.574745 |
| C | -6.131539 | 13.582751 | 0.956063 |
| C | -5.145832 | -10.980552 | -1.231676 |
| C | -6.419221 | -11.304397 | -1.712989 |
| C | -6.911783 | -12.598816 | -1.574748 |
| C | -6.131914 | -13.582585 | -0.956068 |
| C | -4.862434 | -13.269502 | -0.475838 |
| C | -4.359776 | -11.970035 | -0.606736 |

| | | | |
|---|---|---|---|
| C | -3.005243 | -11.657005 | -0.070425 |
| C | -4.657726 | -9.583097 | -1.365584 |
| C | -3.325884 | -9.257901 | -0.781312 |
| C | -2.534137 | -10.248861 | -0.16791 |
| O | -2.307533 | -12.526512 | 0.449286 |
| O | -5.340968 | -8.716055 | -1.907165 |
| C | -2.889942 | -7.936412 | -0.807671 |
| C | -1.299558 | -9.894765 | 0.390246 |
| C | -1.670902 | -7.572496 | -0.207399 |
| C | -0.867193 | -8.574496 | 0.376318 |
| C | 10.231376 | 3.039 | 0.229959 |
| C | 9.268235 | 3.904571 | -0.303068 |
| C | 7.923557 | 3.556684 | -0.30073 |
| C | 7.511965 | 2.321837 | 0.244524 |
| C | 8.475564 | 1.470547 | 0.815123 |
| C | 9.823935 | 1.817414 | 0.802182 |
| C | 10.816835 | 0.854555 | 1.357997 |
| C | 11.670477 | 3.409813 | 0.15291 |
| C | 12.664189 | 2.428876 | 0.672643 |
| C | 12.255389 | 1.214196 | 1.260173 |
| O | 10.457096 | -0.214378 | 1.848278 |
| O | 12.025793 | 4.481923 | -0.334242 |
| C | 14.029045 | 2.719109 | 0.567502 |
| C | 13.214355 | 0.312965 | 1.735338 |
| C | 14.977141 | 1.814773 | 1.040277 |
| C | 14.569546 | 0.610787 | 1.626138 |
| C | 12.664126 | -2.429202 | -0.672649 |
| C | 14.028975 | -2.719468 | -0.567509 |
| C | 14.977092 | -1.815155 | -1.040287 |
| C | 14.569525 | -0.611161 | -1.62615 |
| C | 13.214342 | -0.313306 | -1.735348 |
| C | 12.255354 | -1.214513 | -1.260181 |
| C | 10.816808 | -0.854837 | -1.358003 |
| C | 11.670391 | -3.410114 | -0.152913 |
| C | 10.231299 | -3.039267 | -0.229961 |
| C | 9.823886 | -1.817672 | -0.802186 |
| O | 10.457094 | 0.214103 | -1.848286 |
| O | 12.025682 | -4.482232 | 0.334241 |
| C | 9.268137 | -3.904814 | 0.303069 |
| C | 8.475523 | -1.470773 | -0.815126 |
| C | 7.923467 | -3.556894 | 0.300732 |
| C | 7.511905 | -2.322038 | -0.244524 |
| H | -3.303725 | 0.953859 | -0.081231 |
| H | 0.968566 | 3.422329 | 0.102116 |

| | | | |
|---|---|---|---|
| H | 2.621776 | 2.464956 | 0.137478 |
| H | 2.621711 | -2.465031 | -0.137474 |
| H | 0.968474 | -3.42236 | -0.102113 |
| H | -3.303751 | -0.953776 | 0.081238 |
| H | -8.915658 | 5.657437 | 0.737645 |
| H | -7.110941 | 3.940687 | 0.771854 |
| H | -4.484823 | 6.721688 | -1.222212 |
| H | -10.430411 | 10.268129 | -0.230026 |
| H | -6.003537 | 11.295291 | -2.226481 |
| H | -9.969635 | 12.554719 | -1.114888 |
| H | -7.754015 | 13.068549 | -2.116881 |
| H | -4.485005 | -6.721575 | 1.222215 |
| H | -7.111047 | -3.940499 | -0.771846 |
| H | -8.915811 | -5.657199 | -0.737637 |
| H | -6.003844 | -11.295136 | 2.226481 |
| H | -10.430692 | -10.26785 | 0.230031 |
| H | -7.754372 | -13.068346 | 2.116881 |
| H | -9.969979 | -12.554453 | 1.114891 |
| H | -3.519212 | 7.185204 | 1.268751 |
| H | 0.078426 | 8.302586 | -0.832832 |
| H | -0.698187 | 10.669328 | -0.853052 |
| H | -7.011591 | 10.52517 | 2.177915 |
| H | -4.245459 | 14.018093 | -0.009147 |
| H | -7.904763 | 12.84136 | 1.937392 |
| H | -6.5183 | 14.590499 | 0.844124 |
| H | -7.011879 | -10.524978 | -2.177915 |
| H | -7.905117 | -12.841142 | -1.937394 |
| H | -6.518702 | -14.590322 | -0.844129 |
| H | -4.245845 | -14.017981 | 0.00914 |
| H | -3.519406 | -7.185111 | -1.268749 |
| H | -0.698477 | -10.669317 | 0.853048 |
| H | 0.078202 | -8.302596 | 0.83283 |
| H | 9.597138 | 4.842884 | -0.735189 |
| H | 7.184741 | 4.221292 | -0.734978 |
| H | 8.181868 | 0.52115 | 1.2459 |
| H | 14.326746 | 3.655954 | 0.110055 |
| H | 12.877591 | -0.621196 | 2.169676 |
| H | 16.034157 | 2.043429 | 0.949714 |
| H | 15.309409 | -0.096922 | 1.984929 |
| H | 14.326653 | -3.656319 | -0.11006 |
| H | 16.034102 | -2.043837 | -0.949725 |
| H | 15.309405 | 0.09653 | -1.984943 |
| H | 12.8776 | 0.620862 | -2.169687 |
| H | 9.597018 | -4.843133 | 0.735192 |

| | | | |
|---|---|---|---|
| H | 8.181849 | -0.521369 | -1.245905 |
| H | 7.184636 | -4.221484 | 0.734981 |

## Supplementary References


1 Liu, J. *et al.* Aggregation-induced emission enhancement based on 11,11,12,12,-tetracyano-9,10-anthraquinodimethane. *Chem. Commun.* **49**, 1199–1201 (2013).

2 Gouloumis, A. *et al.* Control over charge separation in phthalocyanine-anthraquinone conjugates as a function of the aggregation status. *J. Am. Chem. Soc.* **128**, 12674–12684 (2006).

3 Choi, J. *et al.* Bicarbonate-enhanced generation of hydroxyl radical by visible light-induced photocatalysis of $H_2O_2$ over $WO_3$: Alteration of electron transfer mechanism. *Chem. Eng. J.* **432**, 134401 (2022).

4 Chen, J., Wang, Y., Wang, F. & Li, Y. Photo-induced switching of $CO_2$ hydrogenation pathway towards $CH_3OH$ production over Pt@UiO-66-$NH_2$(Co). *Angew. Chem. Int. Ed.* **62**, e202218115 (2023).

5 Zhang, X. *et al.* Keto-anthraquinone covalent organic framework for $H_2O_2$ photosynthesis with oxygen and alkaline water. *Nat. Commun.* **15**, 2649 (2024).

6 Zhang, W. *et al.* Reconstructed covalent organic frameworks. *Nature* **604**, 72-79 (2022).

7 Cai, J. *et al.* Crafting mussel-inspired metal nanoparticle-decorated ultrathin graphitic carbon nitride for the degradation of chemical pollutants and production of chemical resources. *Adv. Mater.* **31**, 1806314 (2019).

8 Du, R. *et al.* Controlled oxygen doping in highly dispersed Ni-loaded g-$C_3N_4$ nanotubes for efficient photocatalytic $H_2O_2$ production. *Chem. Eng. J.* **441**, 135999 (2022).

9 Wang, A. *et al.* Facile synthesis of $C_3N_4$/$NiIn_2S_4$ heterostructure with novel solar steam evaporation efficiency and photocatalytic $H_2O_2$ production performance. *Appl. Catal. B-Environ.* **310**, 121336 (2022).

10 Shiraishi, Y. *et al.* Sunlight-driven hydrogen peroxide production from water and molecular oxygen by metal-free photocatalysts. *Angew. Chem. Int. Ed.* **53**, 13454–13459 (2014).

11 Chu, C. *et al.* Spatially separating redox centers on 2D carbon nitride with cobalt single atom for photocatalytic $H_2O_2$ production. *Proc. Natl. Acad. Sci. U. S. A.* **117**, 6376–6382 (2020).

12 Zeng, X. *et al.* Simultaneously tuning charge separation and oxygen reduction pathway on graphitic carbon nitride by polyethylenimine for boosted photocatalytic hydrogen peroxide production. *ACS Catal.* **10**, 3697–3706 (2020).

13 Kofuji, Y. *et al.* Carbon nitride–aromatic diimide–graphene nanohybrids: metal-free photocatalysts for solar-to-hydrogen peroxide energy conversion with 0.2% efficiency. *J. Am. Chem. Soc.* **138**, 10019–10025 (2016).

14 Chen, L. *et al.* Acetylene and diacetylene functionalized covalent triazine frameworks as metal-free photocatalysts for hydrogen peroxide production: a new two-electron water oxidation pathway. *Adv. Mater.* **32**, 1904433 (2020).

15 Shiraishi, Y. *et al.* Resorcinol–formaldehyde resins as metal-free semiconductor photocatalysts for solar-to-hydrogen peroxide energy conversion. *Nat. Mater.* **18**, 985–993 (2019).

16 Shiraishi, Y., Matsumoto, M., Ichikawa, S., Tanaka, S. & Hirai, T. Polythiophene-doped resorcinol–formaldehyde resin photocatalysts for solar-to-hydrogen peroxide energy conversion. *J. Am. Chem. Soc.* **143**, 12590–12599 (2021).

17 Teng, Z. *et al.* Atomically dispersed antimony on carbon nitride for the artificial photosynthesis of hydrogen peroxide. *Nat. Catal.* **4**, 374–384 (2021).

18 Xu, X. *et al.* The construction of conjugated organic polymers containing phenanthrenequinone redox centers for visible-light-driven $H_2O_2$ production from $H_2O$ and $O_2$ without any additives. *Chem. Eng. J.* **454**, 139929 (2023).

19 Wu, C. *et al.* Polarization engineering of covalent triazine frameworks for highly efficient photosynthesis of hydrogen peroxide from molecular oxygen and water. *Adv. Mater.* **34**, 2110266 (2022).

20 Xu, X. *et al.* Conjugated organic polymers with anthraquinone redox centers for efficient photocatalytic hydrogen peroxide production from water and oxygen under visible light irradiation without any additives. *ACS Catal.* **12**, 12954–12963 (2022).

21 Ye, Y.-X. *et al.* Highly efficient photosynthesis of hydrogen peroxide in ambient conditions. *Proc. Natl. Acad. Sci. U. S. A.* **118**, e2103964118 (2021).

22 Ye, Y.-X. *et al.* A solar-to-chemical conversion efficiency up to 0.26% achieved in ambient conditions. *Proc. Natl. Acad. Sci. U. S. A.* **118**, e2202913119 (2021).

23 Yan, H. *et al.* Regulation the reactive oxygen species on conjugated polymers for highly efficient photocatalysis. *Appl. Catal. B-Environ.* **314**, 121488 (2022).

24 Cheng, C. *et al.* In-situ formatting donor-acceptor polymer with giant dipole moment and ultrafast exciton separation. *Nat. Commun.* **15**, 1313 (2024).

25 Yan, H. *et al.* Spontaneous exciton dissociation in organic photocatalyst under ambient conditions for highly efficient synthesis of hydrogen peroxide. *Proc. Natl. Acad. Sci. U. S. A.* **119**, e2202913119 (2022).

26 Gopakumar, A. *et al.* Lignin-supported heterogeneous photocatalyst for the direct generation of $H_2O_2$ from seawater. *J. Am. Chem. Soc.* **144**, 2603–2613 (2022).

27 Kofuji, Y. *et al.* Graphitic carbon nitride doped with biphenyl diimide: efficient photocatalyst for hydrogen peroxide production from water and molecular oxygen by sunlight. *ACS Catal.* **6**, 7021–7029 (2016).

28 Kofuji, Y. *et al.* Mellitic triimide-doped carbon nitride as sunlight-driven photocatalysts for hydrogen peroxide production. *ACS Sustain. Chem. Eng.* **5**, 6478–6485 (2017).

29 Moon, G.-h. *et al.* Eco-friendly photochemical production of $H_2O_2$ through $O_2$ reduction over carbon nitride frameworks incorporated with multiple heteroelements. *ACS Catal.* **7**, 2886–2895 (2017).

30 Kofuji, Y. *et al.* Hydrogen peroxide production on a carbon nitride–boron nitride-reduced graphene oxide hybrid photocatalyst under visible light. *ChemCatChem* **10**, 2070–2077 (2018).
31 Zhu, Z., Pan, H., Murugananthan, M., Gong, J. & Zhang, Y. Visible light-driven photocatalytically active g-$C_3N_4$ material for enhanced generation of $H_2O_2$. *Appl. Catal. B-Environ.* **232**, 19–25 (2018).
32 Shiraishi, Y. *et al.* Solar-to-hydrogen peroxide energy conversion on resorcinol-formaldehyde resin photocatalysts prepared by acid-catalysed polycondensation. *Commun. Chem.* **3**, 169 (2020).
33 Chen, L. *et al.* Simultaneously tuning band structure and oxygen reduction pathway toward high-efficient photocatalytic hydrogen peroxide production using cyano-rich graphitic carbon nitride. *Adv. Funct. Mater.* **31**, 2105731 (2021).
34 Wu, Q. *et al.* A metal-free photocatalyst for highly efficient hydrogen peroxide photoproduction in real seawater. *Nat. Commun.* **12**, 483 (2021).
35 Cheng, H. *et al.* Rational design of covalent heptazine frameworks with spatially separated redox centers for high-efficiency photocatalytic hydrogen peroxide production. *Adv. Mater.* **34**, 2107480 (2022).
36 Zhi, Q. *et al.* Piperazine-linked metalphthalocyanine frameworks for highly efficient visible-light-driven $H_2O_2$ photosynthesis. *J. Am. Chem. Soc.* **144**, 21328–21336 (2022).
37 Liu, T. *et al.* A general interfacial-energetics-tuning strategy for enhanced artificial photosynthesis. *Nat. Commun.* **13**, 7783 (2022).
38 Chen, D. *et al.* Covalent organic frameworks containing dual $O_2$ reduction centers for overall photosynthetic hydrogen peroxide production. *Angew. Chem. Int. Ed.* **62**, e202217479 (2023).
39 Zhao, C. *et al.* Molecular level modulation of anthraquinone-containing resorcinol-formaldehyde resin photocatalysts for $H_2O_2$ production with exceeding 1.2 % efficiency. *Angew. Chem. Int. Ed.* **62**, e202218318 (2023).
40 Chang, J.-N. *et al.* Oxidation-reduction molecular junction covalent organic frameworks for full reaction photosynthesis of $H_2O_2$. *Angew. Chem. Int. Ed.* **62**, e202218868 (2023).
41 Zhang, Y. *et al.* $H_2O_2$ generation from $O_2$ and $H_2O$ on a near-infrared absorbing porphyrin supramolecular photocatalyst. *Nat. Energy* **8**, 361–371 (2023).
42 Tan, H. *et al.* Photocatalysis of water into hydrogen peroxide over an atomic Ga-N5 site. *Nat. Synth.* **2**, 557–563 (2023).
