# Supplementary material for: Achieving a solar-to-chemical efficiency of 3.6% in ambient conditions by inhibiting interlayer charges transport
Source: Nat Commun. 2024 Jun 26;15:5406. doi: 10.1038/s41467-024-49373-z (PMC11208529; doi:10.1038/s41467-024-49373-z)
Supplement: Supplementary file 3 — Description of Additional Supplementary Files [file 41467_2024_49373_MOESM3_ESM.pdf]

Description of Additional Supplementary Files

Supplementary Movie 1. Determination of light intensity in SCC measurement

Supplementary Movie 2. The process of SCC measurement
